# A Writhed Möbius Nanobelt Derived from [7]Helicene

**DOI:** 10.1021/jacs.5c01323

**Published:** 2025-05-16

**Authors:** Liping Ye, Chenyu Hu, Daiyue Yang, Li Zhang, Xiao Chen, Lulin Qiao, Zhifeng Huang, Jun Yang, Qian Miao

**Affiliations:** † Department of Chemistry, 26451 The Chinese University of Hong Kong, Shatin, New Territories, Hong Kong, China; ‡ Department of Chemistry, 25809 The University of Hong Kong, Hong Kong, China; § State Key Laboratory of Synthetic Chemistry, The Chinese University of Hong Kong, Shatin, New Territories, Hong Kong, China; ∥ Shanghai-Hong Kong Joint Laboratory in Chemical Synthesis, Shanghai Institute of Organic Chemistry, 145309 Chinese Academy of Sciences, Shanghai 200032, China; ⊥ State Key Laboratory of Synthetic Chemistry, The University of Hong Kong, Shatin, New Territories, Hong Kong, China; # Shanghai-Hong Kong Joint Laboratory in Chemical Synthesis, The Chinese University of Hong Kong, Shatin, New Territories, Hong Kong, China

## Abstract

A novel writhed Möbius nanobelt was synthesized using a helical building block derived from [7]­helicene and a C-shaped building block derived from pyrene. These two building blocks were connected through nucleophilic aromatic substitution to form an oxanorbornene-containing macrocycle, which was then converted to the nanobelt by reductive aromatization and subsequent oxidation. The structure of the Möbius nanobelt was confirmed with X-ray crystallography. Both the nanobelt and its macrocyclic precursor exhibit *C*_2_ symmetry, but this symmetry is only reflected by the ^1^H NMR signals for the tetra­(4-*t*-butylphenyl)­dinaphthopyrene moiety in the nanobelt, not in its precursor. This difference is attributed to the distinct arrangements of the pendent 4-*t*-butylphenyl groups, caused by the crowdedness and restricted rotation of the C–C single bonds in the nanobelt. Theoretical calculations suggest that the nanobelt does not exhibit global ring currents but has localized aromatic ring currents. Additionally, when an enantiopure form of the [7]­helicene derivative was used, the nanobelt was obtained in an enantiopure form, showing an absorption dissymmetry factor of 4 × 10^–3^.

## Graphical abstract

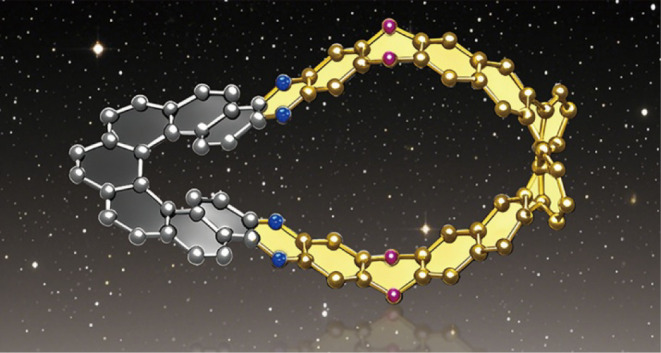

## Introduction

Möbius strips have long captivated mathematicians, artists, designers, and architects with their unique one-sided, nonorientable surface.^1^ In the field of chemistry, interest in Möbius topology was sparked in 1964 when Heilbronner predicted that [4*n*]­annulenes with Möbius topology exhibit aromaticity,^2^ contrary to the conventional expectations of Hückel’s rule. The concept of Möbius aromaticity and antiaromaticity was later applied by Zimmerman to monocyclic arrays of orbitals having one or an odd number of overlaps between adjacent orbitals of different signs.^3^ For instance, the Hückel and Möbius aromaticities in the transition states of pericyclic reactions offer an alternative perspective to the Woodward–Hoffmann rule.^4^ π-Conjugated molecules of Möbius topology are appealing yet challenging targets of organic synthesis, as they suffer considerable destabilization caused by ring strains and contortion, which hinder the overlap of *p* orbitals and counteract the stabilization resulting from Möbius aromaticity.

The Möbius strip is a closed twisted ribbon having a linking number (*L*_k_) of 1. According to the Călugăreanu theorem, *L*_k_ = *T*_w_ + *W*_r_, where *T*_w_ and *W*_r_ are topological parameters denoting twist and writhe, respectively.^5^ *T*_w_ is the number of 180° rotations along the axis of the ribbon, while *W*_r_ is the sum of self-crossings when the object is viewed parallel to the three Cartesian axes.^6^ A Möbius strip is typically created by connecting the two ends of a strip with a 180° twist, resulting in *T*_w_ = 1 and *W*_r_ = 0, as shown in Figure 1a. An alternative way to form a Möbius strip is connecting the two ends of a strip through a full-turn helix as shown in Figure 1a, resulting in a self-crossing. In this case, *T*_w_ = 0 and *W*_r_ = 1. Herein, we propose to name a Möbius strip with *T*_w_ = 1 and *W*_r_ = 0 as the twisted Möbius strip and a Möbius strip with *T*_w_ = 0 and *W*_r_ = 1 as the writhed Möbius strip. The first reported stable Möbius aromatic molecule is Herges’ [16]­annulene derivative (Figure 1b).^7^ It was designed as a writhed Möbius strip although careful topological analysis of the crystal structure later revealed *T*_w_ = 0.46 and *W*_r_ = 0.54 for it.^6^ Unlike Herges’ design, the Möbius annulene moieties in the expanded porphyrins^8,9^ are twisted Möbius strips. Replacing the vinylene groups in Möbius annulenes with larger π-units, such as 1,4-phenylene and *para*-phenylmethine, leads to Möbius nanorings or nanohoops,^10–12^ which are single-stranded macrocycles.

**Figure 1:**
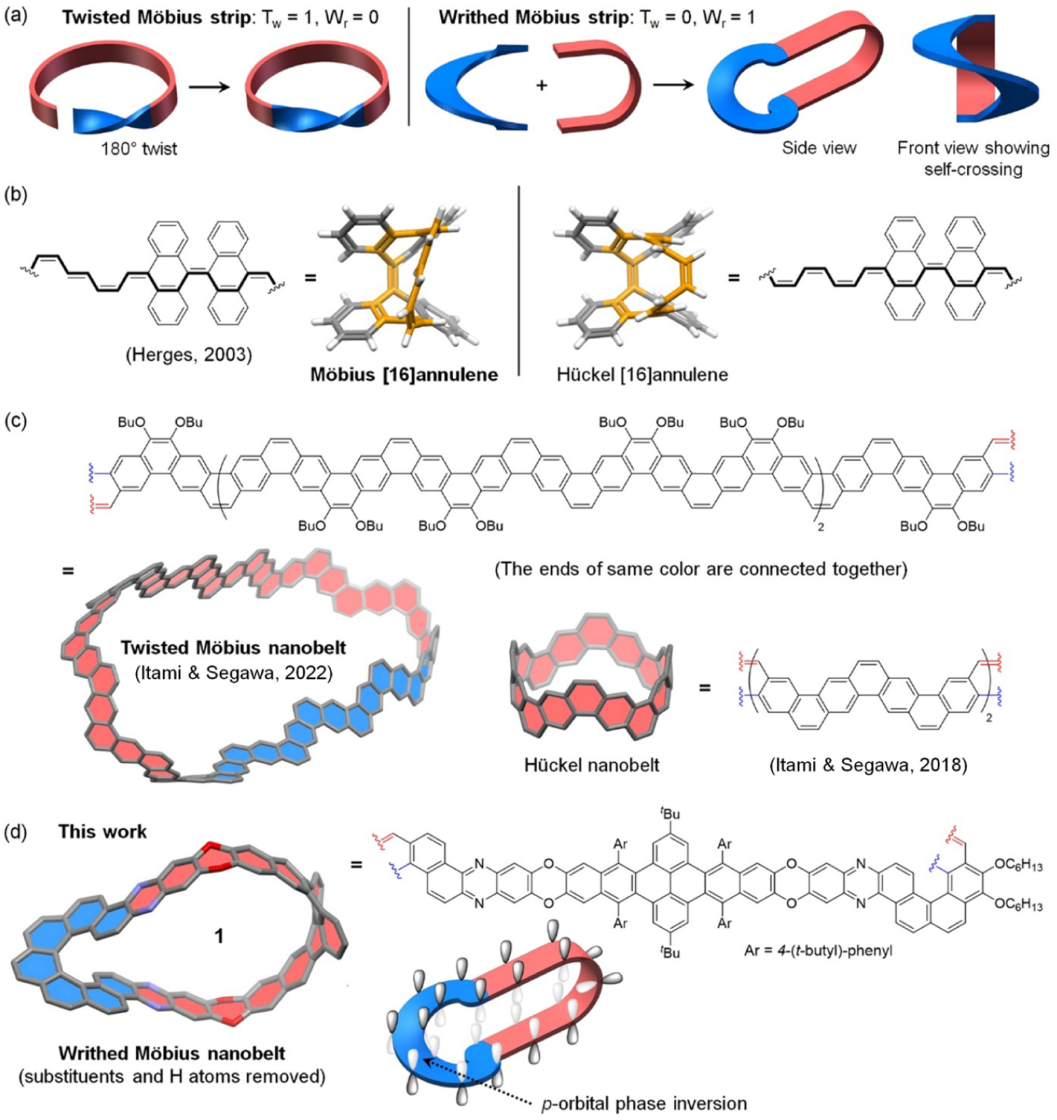
(a) Schematics of twisted and writhed Möbius strips; (b) Möbius and Hückel [16]­annulenes synthesized by Herges; (c) Möbius and Hückel nanobelts synthesized by Itami and Segawa; (d) writhed Möbius nanobelt **1** with *p*-orbital phase inversion.

Besides its one-sided, nonorientable surface, a Möbius strip is characterized by having only one edge. This characteristic can be demonstrated in two ways. One way is tracing a path along the edge of a Möbius strip, where one can observe that it eventually returns to its starting point on the same edge, encompassing all the boundary points of the strip in a continuous curve. The other way is cutting a Möbius strip lengthwise in the middle, which does not yield two separate strips but rather a single strip with double the original length. Notably, this one-edge characteristic is not reflected by the conjugation pathway of a Möbius annulene or a Möbius nanoring due to the C–C single bonds linking π-units. An annulene or a nanoring may have Möbius topology or Hückel topology depending on whether it exhibits p-orbital phase inversion. However, both Möbius and Hückel annulenes have only one conjugation pathway in a circular form.

Unlike Möbius annulenes, conjugated Möbius nanobelts fully resemble Möbius strips by having both a one-sided surface and a one-edge conjugation pathway. A conjugated Möbius nanobelt exclusively consisting of *sp* ^2^ carbons was recently synthesized by the group of Itami and Segawa,^13^ while that containing sulfur atoms was synthesized by the group of Zhu and Mu.^14^ On the other hand, the Möbius nanobelt synthesized by Tanaka et al.^15^ lacks a complete one-edge conjugation path-way due to the presence of methylene groups. Figure 1c shows Itami’s Möbius nanobelt as an example and compares it with the corresponding Hückel nanobelt.^16,17^ The Möbius nanobelt has only one edge as a result of linking the two ends of nanoribbon with a 180° twist, whereas the Hückel nanobelt presents upper and lower edges that are conjugated but do not coincide.^18^ Further increase of *T*_w_ leads to triply twisted Möbius nanobelts, which were recently synthesized by Wu and Isobe,^19^ as well as Tanaka and Hashizume.^20^

Herein, we report a writhed Möbius nanobelt (**1** in Figure 1d), which is designed by fusing [7]­helicene with a fragment of *O*,*N*-heterocyclacene. The [7]­helicene component (shown in blue) provides a full-turn helix, and the partial *O*,*N*-heterocyclacene moiety (shown in brick red) features radially arranged *p*-orbitals with in-plane alignment. Connecting the two moieties together creates p-orbital phase inversion, as shown in Figure 1d. Notably, Möbius nanobelt **1** differs from the recently reported Möbius nanorings that have a helicene moiety embedded in a cycloparaphenylene (CPP)^21–23^ or have helicene units linked through C–C single bonds forming a single-stranded macrocycle.^24–26^ Detailed below are the synthesis of **1** in both racemic and enantiopure forms, along with a study on its molecular structures and electronic properties using experimental and computational methods.

## Result and Discussion

Scheme 1 shows the synthesis of Möbius belt **1**, with compound **2** serving as the key intermediate. Compound **2** was produced from a helical building block (**3a**) and a C-shaped building block (**4**). Building block **3a** was synthesized from dibromophenanthrene **5** through a four-step process. Initially, the Heck reaction of **5** with 2,3-dimethoxystyrene (**6**) yielded dialkene **7a** as the (*E*,*E*) isomer, which subsequently underwent photocyclization to afford the corresponding substituted [7]­helicene (**8a**). The demethylation of **8a** with BBr_3_, followed by oxidation with pyridinium dichromate (PDC), resulted in bis­(*ortho*-quinone) **9a**, which was then condensed with 4,5-difluorobenzene-1,2-diamine to yield compound **3a**, a π-extended [7]­helicene. In addition, compound **3b**, an analogue of **3a** without *n*-hexoxy and fluorine substituents was synthesized using a similar way as detailed in the Supporting Information, and successfully characterized with single crystal X-ray crystallography. Building block **4** was synthesized by reducing compound **10**, a C-shaped bis­(ortho-quinone) synthesized by us earlier,^27,28^ using sodium dithionite. The condensation of building blocks **3a** and **4** through nucleophilic aromatic substitution under a basic condition led to compound **2**. It is worth noting that the reactive sites in **3a** and **4** are separated by similar distances. C1 and C2 in **3a** are separated by 8.51 Å, as measured from the crystal structure of its analogue (**3b**), while O1 and O2 in compound **4** are separated by 9.14 Å, as measured from the energy-minimized model. The similar distances between C1 and C2 in **3a** and O1 and O2 in **4** presumably facilitate the macrocyclization process. Treatment of **2** with NaI/trimethylsilyl iodide (TMSI)^27,29^ enabled reductive aromatization, resulting in an overhydrogenated product **11** in a yield of 53%. Further oxidation of **11** with 2,3-dichloro-5,6-dicyano-1,4-benzoquinone (DDQ) afforded Möbius belt **1** as a yellow solid in quantitative yield. The molecular formula of **11** was determined by high-resolution mass spectrometry (HRMS) as C_134_H_124_N_4_O_6_, which contains four additional hydrogen atoms compared to **1** (C_134_H_120_N_4_O_6_). The structure of **11**, specifically the position of hydrogenation (highlighted with gray rings in the structure of **11** in Scheme 1) was established by comparing its ^1^H NMR spectrum to that of compound **1**. Möbius nanobelt **1** is chiral and was initially obtained as a racemic mixture through the described procedures. However, when the enantiopure form of **3a**, obtained by chiral resolution, was used in the synthesis, Möbius belt **1** was also obtained in an enantiopure form.

**Scheme 1:**
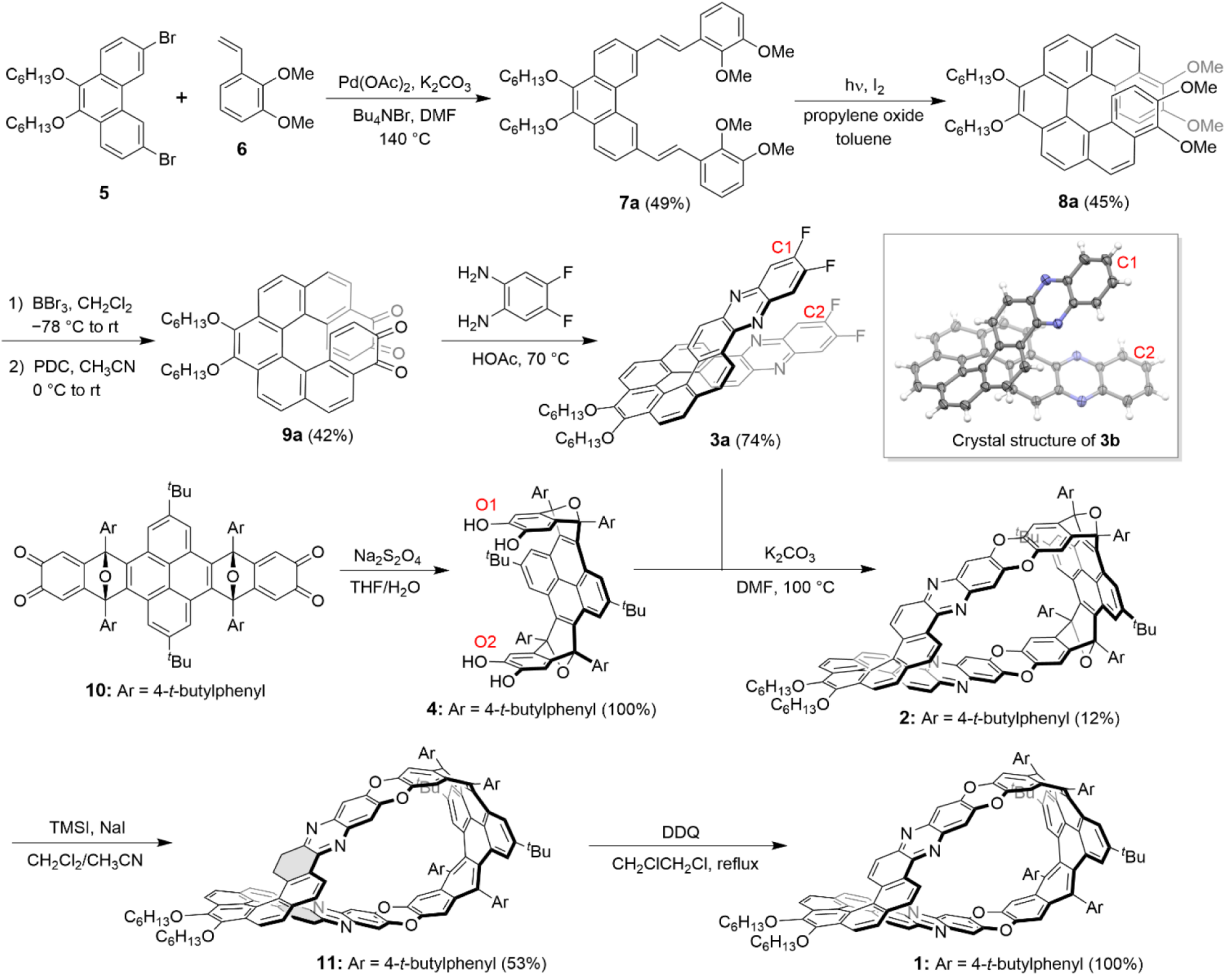
Synthesis of Möbius Belt **1** with the Crystal Structure of **3b**

Given that the conjugation along nanobelt **1** is limited by the presence of dioxin rings, replacing these rings with pyrazine rings or avoiding them altogether could enhance the conjugation within the macrocycle. However, our preliminary attempts to synthesize such Möbius nanobelts were unsuccessful during either the condensation step or the reductive aromatization, as detailed in the Supporting Information.

Single crystals of (−)-**1** were obtained by slow evaporation of its solution in CH_2_Cl_2_. The structure of nanobelt **1** was unambiguously determined using X-ray crystallography, with an absolute *M*-helical configuration arbitrarily assigned to (−)-**1**, as shown in Figure 2. In the crystal, nanobelt **1** (excluding the substituting hexyl groups) exhibits approximate *C*_2_ symmetry, with the *C*_2_ axis crossing the centers of the [7]­helicene and pyrene moieties, as shown in the top view. The nanobelt displays slight differences in bending at the two dioxin moieties, with dihedral angles of 127° and 131° measured between the two benzene rings linked by oxygen atoms. The space within the nanobelt is occupied by disordered solvent molecules, which were removed using the PLATON/SQUEEZE program.

**Figure 2:**
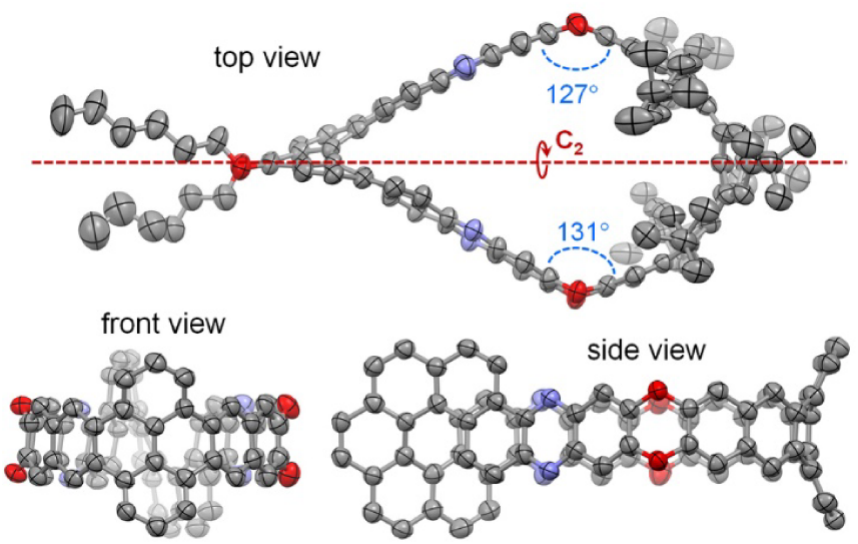
Structures of (−)-**1** in the crystal. (For clarity, H atoms and disordered atoms are not shown and substituting groups in the front and side views are removed. C, N and O atoms are shown as ellipsoids set at 50% probability.)

One notable finding from structural characterization of compounds **1** and **2** is the difference in their ^1^H NMR spectra. Figure 3 compares the ^1^H NMR signals of compounds **1** and **2** in the aromatic region, with the signals assigned to the respective protons based on the 2D ROESY NMR spectra (Figures S20–S22 and S28–S31). These NMR spectra were recorded from their solution in CD_2_Cl_2_ at 294 K or in C_2_D_2_Cl_4_ at 363 K with CS_2_ added due to the low solubility of compounds **1** and **2** at room temperature. Compounds **1** and **2** exhibit similar signals for the [7]­helicene and diazadioxatetracene moieties, namely, six doublets for protons a–f and four singlets for protons g/g′ and h/h′ (see Figure 3 for the labels of protons). However, apparent differences are observed for the protons on the central pyrene moiety and the pendent 4-*tert*-butylphenyl groups. In compound **2**, protons i–l and i′–l′ in the 4-*tert*-butylphenyl groups exhibit two doublets, indicating that the C–C single bond (colored green in the front view in Figure 4b) between the phenyl group and the macrocycle rotates freely, allowing fast exchange of protons i and j as well as k and l. In contrast, protons i–l and i′–l′ in compound **1** exhibit four sets of resonances, indicating restricted rotation of the C–C single bond (colored green in the front view in Figure 4a). Protons i/i′ and j/j′ placed inside the belt are upfield shifted relative to protons k/k′ and l/l′ placed outside the belt, similar to those reported for a zigzag carbon nanobelt with pendent 4-*tert*-butylphenyl groups.^30^ Remarkably, protons i and i′ appear as two double–doublets, indicating different chemical environments. Protons m and m′ on the central pyrene moiety in compound **2** show one singlet, while in compound **1** they appear as two upfield-shifted doublets, indicating different chemical environments.

**Figure 3:**
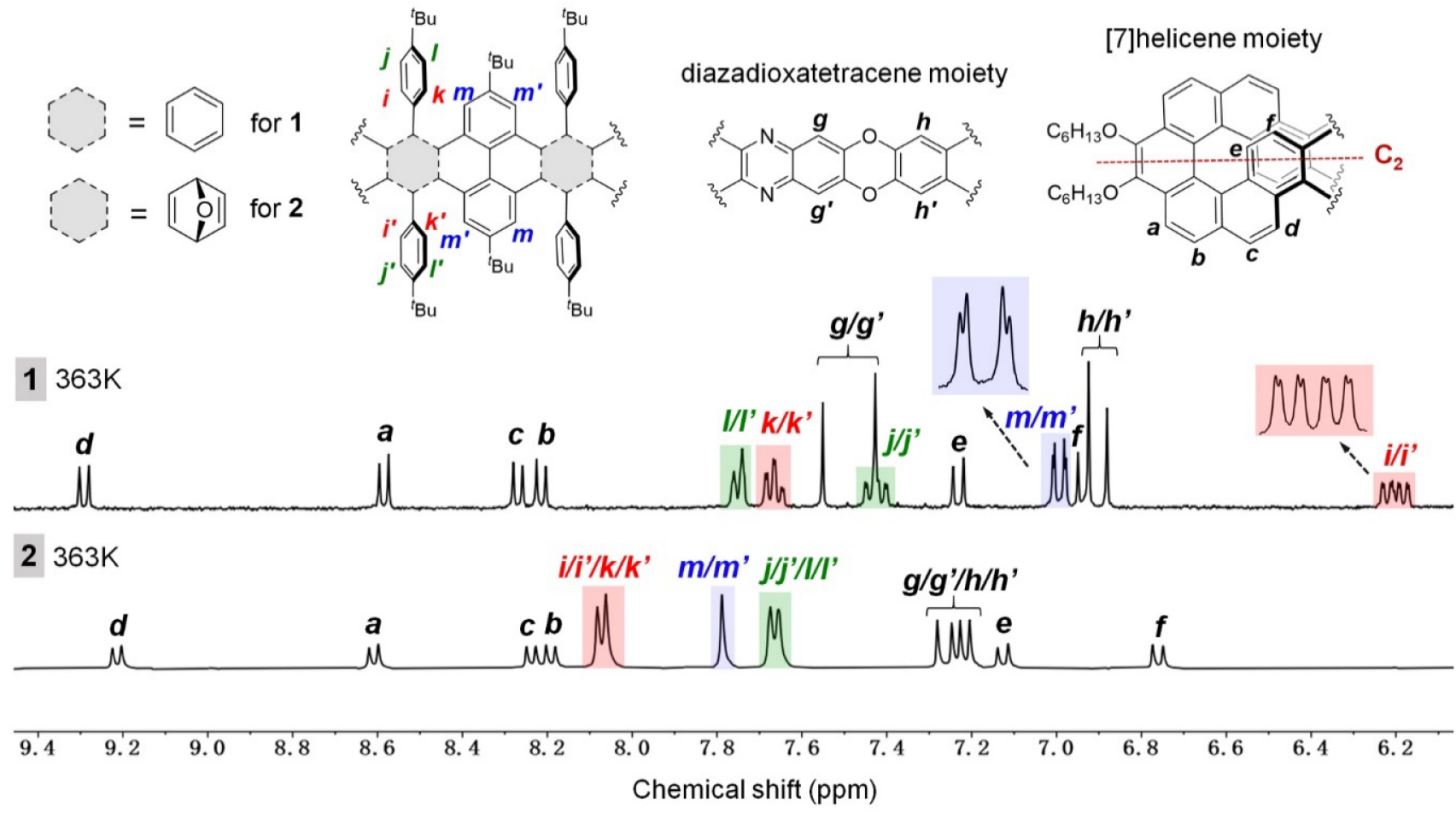
Partial ^1^H NMR spectra of **1** and **2** both in C_2_D_2_Cl_4_ with CS_2_ at 363 K with assignment of the signals.

**Figure 4:**
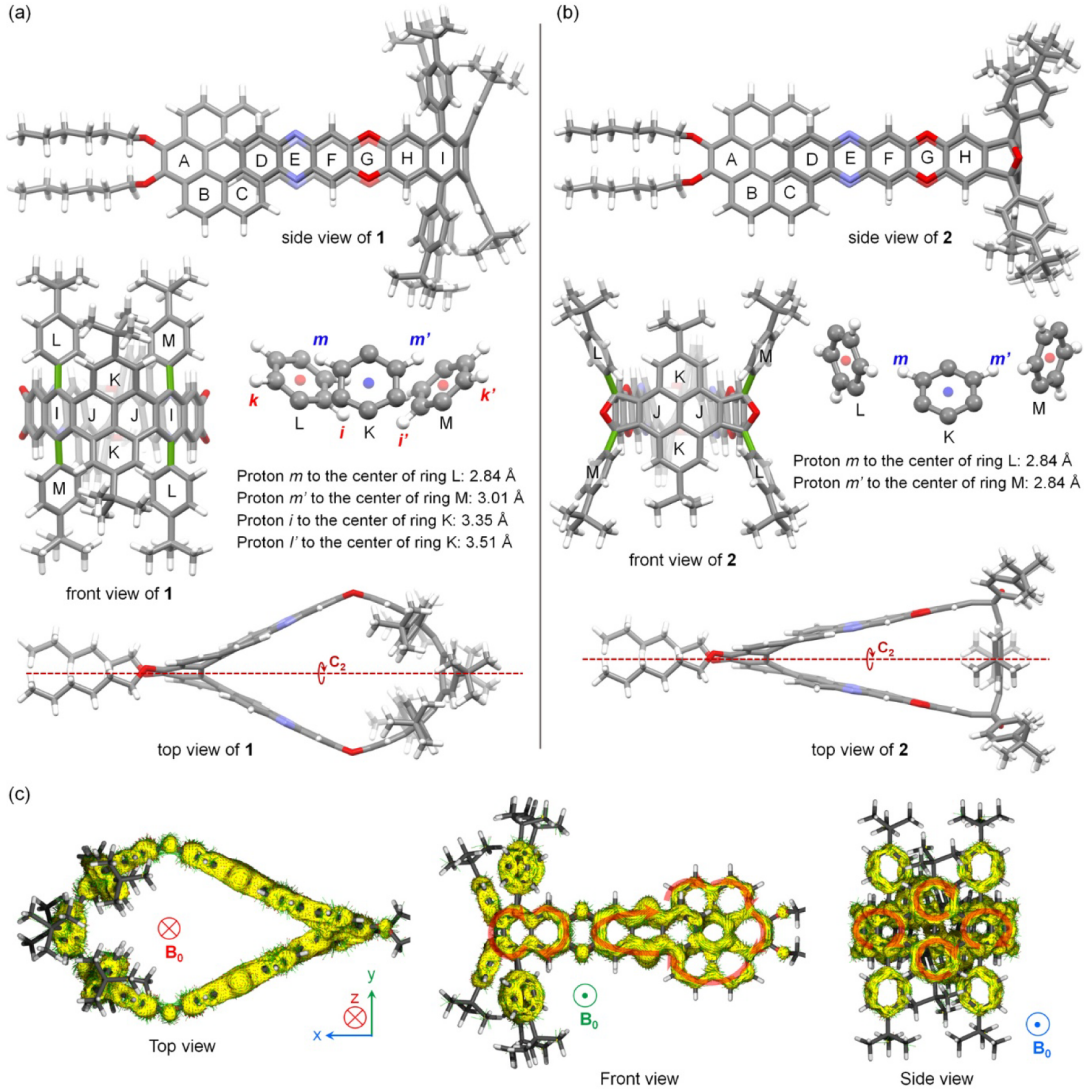
(a) DFT-optimized structure of compound **1** with selected rings and atoms labeled; (b) DFT-optimized structure of compound **2** with selected rings and atoms labeled; (c) calculated ACID plots of **1** with an isovalue of 0.05 from contributions of π-electrons, induced by the external magnetic field (*B*_0_) along the direction of *z* in the top view, along the direction of y in the front view, and along the direction of *x* in the side view. (DFT calculations were conducted at the B3LYP-D3­(BJ)/6–311g­(d,p) level of theory.)

To understand the differences observed in the^1^ H NMR spectra of compounds **1** and **2**, we compared their structures, which were optimized using density functional theory (DFT) calculations at the B3LYP-D3 (BJ) level with the 6-311G­(d,p) basis set. Figure 4a,b shows the optimized structures of compounds **1** and **2**, respectively. Both structures exhibit *C*_2_ symmetry, with the *C*_2_ axis passing through the centers of the [7]­helicene and pyrene moieties, as illustrated in the top views. The differentiation of protons m′ and i′ from protons m and i in the ^1^H NMR spectrum of compound **1** is consistent with the *C*_2_ symmetry, whereas the *C*_2_ symmetry of compound **2** is not reflected by the ^1^H NMR resonances of protons i–m and i′–m′. As shown in the top view (Figure 4a), the pyrene moiety (rings K and J) in compound **1** is bent outward, whereas in compound **2** it is almost flat. Bond length analysis (Figure S48) reveals that the pyrene moieties in both compounds exhibit *C*_2_ symmetry, lacking a plane of symmetry to exchange protons m and m′. However, the pyrene moieties in both compounds only slightly deviate from plane symmetry, providing essentially the same chemical environments to protons i and i′ as well as m and m′. Instead, the chemical environments of protons m and m′ in compound **1** are differentiated by the pendent 4-*tert*-butylphenyl groups, rings L and M. As shown in Figure 4a, protons m and m′ are oriented above rings L and M, respectively. Consequently, they are shielded by the ring currents of L and M, exhibiting an upfield shift relative to those in compound **2**. The distance between proton m and the center of ring L is 2.84 Å, whereas that between proton m′ and the center of ring M is 3.01 Å, creating sufficiently different chemical environments for the two protons. Similarly, protons i and i′ are oriented below ring K, exhibiting an upfield shift relative to those in compound **2** due to the shielding effect of the ring current of K. Protons i and i′ are 3.35 Å and 3.51 Å away from the center of ring K, respectively, resulting in sufficiently different chemical environments. This analysis based on DFT-optimized structures is consistent with the crystal structure of **1**, which also reveals different chemical environments for protons m and m′ as well as i and i′. For example, the average distance between proton *m* and the center of ring L is 2.98 Å, while that between proton m′ and the center of ring M is 3.16 Å, respectively. In contrast, rings L and M in compound **2** exhibit the same distances (2.84 Å) from their centers to protons m and m′, respectively, as shown in Figure 4b, making the chemical environments of protons m and m′ essentially identical. The different arrangements of the pendent 4-*tert*-butylphenyl groups in compounds **1** and **2** can be attributed to the crowdedness and the rotation of the C–C bonds (colored green in the front view in Figure 4a,b) between them and the macrocycle. In compound **1**, the environment of the 4-*tert*-butylphenyl group is crowded, and the rotation of the green C–C bond is restricted, enhancing the arrangement of the 4-*tert*-butylphenyl groups in a *C*_2_ symmetry. On the other hand, the green C–C bonds in compound **2** can rotate freely, averaging the effect of the ring currents in rings L and M. To validate this interpretation, we evaluated the Gibbs free energy change during rotation of the C–C bond between the 4-*tert*-butylphenyl group and the macrocycle through DFT calculations (Figure S51). The results reveal that rotation of this bond in compound **1** involves an energy barrier of at least 26.8 kcal/mol, whereas in compound **2**, the barrier is as low as 6.5 kcal/mol. As a result, in addition to the DFT-calculated structures corresponding to 0 K, the rotational isomerism at room temperature around the unrestricted C–C single bond of compound **2** can vary local environments for protons m/m′ and i/i′ on the kinetic average.

The aromaticity of nanobelt **1** was investigated theoretically using nucleus-independent chemical shift (NICS) and anisotropy of the induced current density (ACID) calculations. For NICS(1) calculations, ghost atoms were placed both outside and inside the macrocycles of compounds **1** and **2**, resulting in NICS(1) and NICS(−1) values for each ring, respectively. The rings in the two compounds are labeled in Figure 4a,b. As summarized in Table 1, rings A–F in both compounds **1** and **2** exhibit NICS(1) and NICS(−1) values ranging from −6.82 to −13.06 ppm, indicating their aromatic nature. Notably, ring B in both compounds shows significant differences between the NICS(1) and NICS(−1) values. This discrepancy arises because ring B is the most contorted among these rings, resulting in different chemical environments on its two sides. Ring G in both compounds **1** and **2** exhibits NICS(1) values with small magnitudes, indicating its nonaromatic nature. The negative NICS(−1) value (−3.52 ppm) of ring G in compound **1** can be attributed to its curved geometry, which places the ghost atom for NICS(−1) close to the shielding regions of the adjacent aromatic rings (F and H). Ring G in compound **1** is bent to provide the necessary curvature for the belt, while in compound **2**, it is flat because the oxanorbornene moieties already provide the necessary curvature. The NICS values of rings H, I, and K in compound **1** indicate their aromatic nature, while the smaller magnitude of those for ring J indicates lower aromaticity, in agreement with the predicted aromatic sextets according to Clar’s rule. The NICS(−1) values of rings H–J are more negative than the corresponding NICS(1) values, indicating that the inside of the belt is more shielded than the outside. This observation aligns with the reported iso-chemical shielding surface (ICSS) map of Chi’s zigzag carbon nanobelt.^30^ The NICS values indicate that ring J in compound **2** is more aromatic than in compound **1**, in agreement with the distribution of aromatic sextets in the resonance structures. Figure 4c presents the ACID plots of nanobelt **1**, calculated with the external magnetic field oriented in three orthogonal directions. When the external magnetic field is perpendicular to the macrocyclic plane (along the *z* direction), no global ring currents are observed along the macrocycle. When the external magnetic field is within the plane of the macrocycle (along the *x* or *y* direction), diamagnetic ring currents are observed in the diquinoxalino[7]­helicene (rings A–F) and dinaphthopyrene (rings H–K) moieties. These NICS and ACID calculation results, which are in agreement with each other, indicate that nanobelt **1** does not exhibit global ring currents in the macrocycle but instead shows localized aromatic ring currents, which are separated by the oxygen atoms in the dioxin rings. Experimentally, the difference in the ^1^H NMR spectra of compounds **1** and **2** can in principle shed light on the possible global ring current along the one-edge conjugation pathway. The absence of global ring currents, as indicated by the NICS and ACID calculations, aligns with our attribution of the spectral differences to the distinct arrangements of the pendent 4-*tert*-butylphenyl groups in these compounds.

**Table 1:** NICS Values of **1** and **2**

	Compound **1**		Compound **2**	
Ring	NICS(1) (ppm)	NICS(−1) (ppm)	NICS(1) (ppm)	NICS(−1) (ppm)
A	–9.51	–9.54	–9.38	–9.38
B	–9.61	–6.82	–9.12	–7.49
C	–11.72	–9.50	–10.82	–9.86
D	–9.32	–8.15	–9.30	–9.36
E	–12.72	–13.01	–12.38	–13.06
F	–10.44	–11.53	–10.02	–10.27
G	1.62	–3.52	2.69	2.10
H	–9.10	–12.49	–8.40	–10.47
I	–7.16	–12.46	–	–
J	–1.30	–5.61	–6.68	–10.10
K	–10.41	–9.71	–12.91	–14.79

The stability of compound **1** was monitored using ^1^H NMR spectroscopy. Upon exposure of a solution of **1** in CD_2_Cl_2_ to air under ambient conditions for 20 h, new signals emerged in the spectrum, as shown in Figure S37. After additional 22 h of air exposure, a degradation product was isolated using preparative thin layer chromatography. The molecular formula of this degradation product was determined by HRMS as C_134_H_120_N_4_O_10_, indicating the addition of four oxygen atoms to compound **1**. The ^1^H NMR spectrum of the degradation product (Figure S37) reveals a loss of the 2-fold symmetry present in compound **1**. In contrast, when a solution of **1** in CD_2_Cl_2_ was exposed to air in the absence of light for 24 h, no changes were observed in its ^1^H NMR spectrum. Similarly, the solid form of **1** stored in air and darkness remained stable for at least 1 week. These observations suggest that the degradation of compound **1** is due to photoinduced oxygenation.^31–33^ The degradation of **1** is presumed to be associated with the inherent strain in its Möbius belt structure. The strain energy of **1** is estimated to be 34 kcal/mol based on the enthalpy change calculated for a hypothetical homodesmotic reaction (Figure S52). This value is approximately half of the strain energies reported for structurally related (18,0)^34^ and (12,0)^30^ zigzag carbon nanobelts, which are 63.3 and 72.9 kcal/mol, respectively. This result is consistent with the structural relationship between nanobelt **1** and the (12,0) zigzag carbon nanobelt, as **1** contains half of the (12,0) nanobelt structure, which is the primary source of the overall strain in **1**. In comparison to nanobelt **1**, compound **2** is estimated to have a smaller strain of 7.7 kcal/mol (Figure S52), due to its oxanorbornene moieties with intrinsic curvature.

The photophysical properties of compounds **1** and **2** were studied using UV–vis absorption and photoluminescence spectroscopy, while their chiroptical properties were investigated using circular dichroism (CD) and circularly polarized luminescence (CPL) spectroscopy. Figure 5a compares the UV–vis absorption spectra of **1** and **2** in CH_2_Cl_2_, showing that the longest wavelength absorption maxima for compound **1** and **2** occur at 455 and 465 nm, respectively. Both molecules exhibit essentially the same absorption edge at approximately 490 nm, corresponding to an optical gap of 2.53 eV. This observation aligns with the nearly identical DFT-calculated gaps between the highest occupied molecular orbital (HOMO) and the lowest unoccupied molecular orbital (LUMO), 2.86 eV for **1** and 2.82 eV for **2**, although the experimental optical gap is smaller than the calculated values. As shown in Figure S50, the HOMO of **1** is primarily located on the dinaphthopyrene moiety, while its LUMO is mainly situated on the diquinoxalino[7]­helicene moiety. Similarly, HOMO of **2** is mainly located on the pyrene moiety, with its LUMO located on the diquinoxalino[7]­helicene moiety. Consequently, the excited states (S_1_) of both **1** and **2**, characterized by charge separation, can be stabilized by polar solvents such as CH_2_Cl_2_, leading to a reduced optical gap.

**Figure 5:**
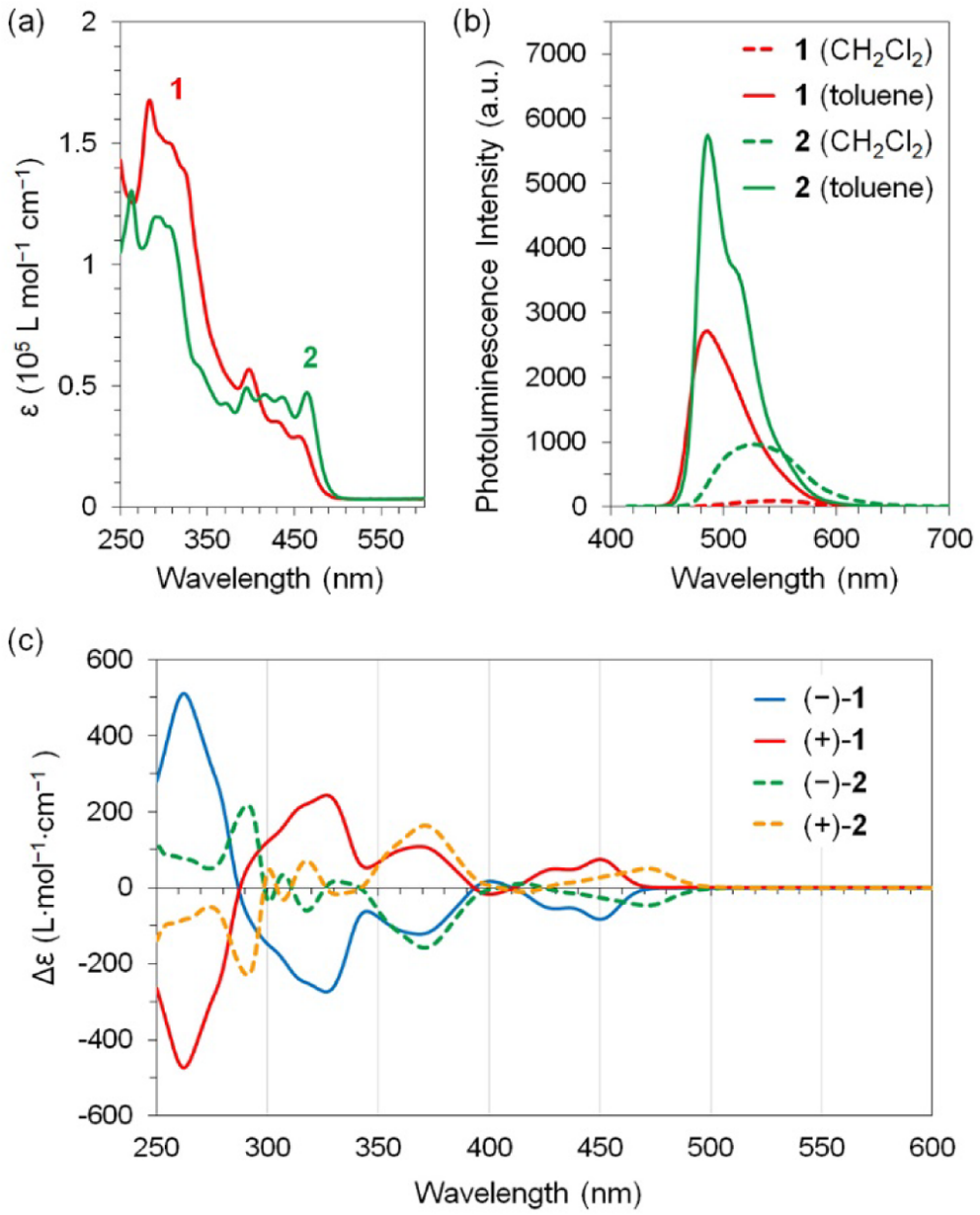
(a) UV–vis absorption of **1** and **2** in CH_2_Cl_2_ (1 × 10^–5^ mol/L) excited at 396 nm; (b) photoluminescence spectra of **1** and **2** in CH_2_Cl_2_ and toluene (1 × 10^–5^ mol/L); (c) circular dichroism (CD) spectra of **1** and **2** in CH_2_Cl_2_.

The charge separation in the excited states of **1** and **2** was further studied computationally and experimentally. DFT calculations on accumulated Mulliken charges indicate that excitation from the S_0_ ground state of **1** to the S_1_ excited state is accompanied with a charge transfer of 0.62 e in vacuum or 0.76 e in CH_2_Cl_2_ (see Table S3). Experimentally, we recorded the UV–vis absorption and photoluminescence spectra of the two compounds in CH_2_Cl_2_ and toluene, two solvents with distinct dielectric constants of 8.93 and 2.38, respectively. The UV–vis absorption spectra of both compounds are nearly identical in both solvents, whereas their photoluminescence spectra appear sensitive to solvent polarity, as depicted in Figure 5b. Both compounds display red-shifted and weaker emission in CH_2_Cl_2_ compared to toluene. Specifically, the absolute fluorescence quantum yield of **1** is measured as 2.9% in CH_2_Cl_2_ and 24.3% in toluene, while that of **2** is measured as 15.2% in CH_2_Cl_2_ and 44.6% in toluene. Additionally, the fluorescence lifetime (τ_1_) for **1** is 5.62 ns in CH_2_Cl_2_ and 3.75 ns in toluene, and for compound **2** it is 6.82 ns in CH_2_Cl_2_ and 3.88 ns in toluene. The red-shifted and weaker emission in CH_2_Cl_2_ can be attributed to better stabilization of the charge-separated excited state by the polar solvent.^28,35^ The stabilized excited state has a longer lifetime, increasing the possibility of nonemissive transitions.

Figure 5c compares the CD spectra of the optically pure forms of **1** and **2**, where their enantiomers exhibit approximate mirror images. Compound **1** exhibits maximum |Δε| values of 511 L·mol^–1^· cm^–1^ at 263 nm, 273 L·mol^–1^· cm^–1^ at 326 nm, 122 L·mol^–1^· cm^–1^ at 369 nm, and 82 L·mol^–1^· cm^–1^ at 450 nm. The corresponding absorption dissymmetry factor, *g*_abs_ = |Δε|/ε,^36,37^ are thus calculated as 4.1 × 10^–3^ at 263 nm, 2.0 × 10^–3^ at 326 nm, 2.2 × 10^–3^ at 369 nm, and 2.9 × 10^–3^ at 450 nm. For compound **2**, the maximum |Δε| values are 229 L·mol^–1^· cm^–1^ at 291 nm, 158 L·mol^–1^· cm^–1^ at 371 nm, and 51 L·mol^–1^· cm^–1^ at 471 nm, giving dissymmetry factors of 1.7 × 10^–3^ at 291 nm, 3.4 × 10^–3^ at 371 nm, and 1.1 × 10^–3^ at 471 nm. The higher dissymmetry factors of **1** compared to **2** are presumably related to its greater extent of conjugation. Notably, the dissymmetry factors of **1** are comparable to those reported for Möbius nanorings containing [5]­helicene moieties (2.7–3.3 × 10^–3^).^22^ The optically pure forms of **1** and **2** also exhibit circularly polarized luminescence (CPL) activity within the wavelength range of 480 to 650 nm (Figures S46 and S47). The luminescence dissymmetry factors (*g*_lum_) of **1** and **2** are approximately 1 × 10^–3^ and 2 × 10^–3^, respectively. For context, the *g*_lum_ values for typical organic circularly polarized emitters are generally on the order of 10^–3^.^38^

## Conclusions

In conclusion, this study presents the design and synthesis of a novel writhed Möbius nanobelt (**1**). The nanobelt was successfully synthesized using a helical building block (**3a**), derived from [7]­helicene, and a C-shaped building block (**4**), derived from pyrene. The connection of these two building blocks through nucleophilic aromatic substitution resulted in macrocycle **2**, which was subsequently converted to an overhydrogenated product (**11**) by reductive aromatization. Finally, oxidation of **11** afforded nanobelt **1**, whose structure was determined with X-ray crystallography. Both molecules **1** and **2** exhibit *C*_*2*_ symmetry. However, this symmetry is reflected by the ^1^H NMR signals for the tetra­(4-*t*-butylphenyl)­dinaphthopyrene moiety in nanobelt **1**, but not in its macrocyclic precursor (**2**). This intriguing difference can be attributed to the distinct arrangements of the pendent 4-*tert*-butylphenyl groups, resulting from the crowdedness and restricted rotation of the C–C single bonds in nanobelt **1**. Theoretical calculations indicate that nanobelt **1** does not exhibit global ring currents in the macrocycle but instead has localized aromatic ring currents. When the enantiopure form of the [7]­helicene derivative (**3a**) was used as a starting material in the synthesis, nanobelt **1** was obtained in an enantiopure form, exhibiting an absorption dissymmetry factor of 4 × 10^–3^.

## Supplementary Material



## Notes

Details of synthesis, characterization of new compounds, DFT calculations, fabrication and characterization of thin films and field effect transistors (PDF)
