# Supplementary material for: A Writhed Möbius Nanobelt Derived from [7]Helicene
Source: J Am Chem Soc. 2025 May 16;147(21):17795–803. doi: 10.1021/jacs.5c01323 (PMC12123616; doi:10.1021/jacs.5c01323)
Supplement: Supplementary file 1 [file ja5c01323_si_001.pdf]

Supplementary Information for

A Writhed Möbius Nanobelt Derived from [7]Helicene

Liping Ye,[a, #] Chenyu Hu,[b, #] Daiyue Yang,[a, c, #] Li Zhang,[a] Xiao Chen,[a] Lulin Qiao,[e] Zhifeng Huang,[a] Jun Yang,*[, b, d] Qian Miao*[, a, c, f]

a Department of Chemistry, The Chinese University of Hong Kong, Shatin, New Territories, Hong Kong, China
b Department of Chemistry, The University of Hong Kong, Hong Kong, China
c State Key Laboratory of Synthetic Chemistry, The Chinese University of Hong Kong, Shatin, New Territories, Hong Kong, China
d State Key Laboratory of Synthetic Chemistry, The University of Hong Kong, Hong Kong, China
e Shanghai-Hong Kong Joint Laboratory in Chemical Synthesis, Shanghai Institute of Organic Chemistry, Chinese Academy of Sciences, Shanghai, 200032, China
f. Shanghai-Hong Kong Joint Laboratory in Chemical Synthesis, The Chinese University of Hong Kong, Shatin, New Territories, Hong Kong, China

[#] Equal contribution
* Email of the corresponding author: juny@hku.edu, miaoqian@cuhk.edu.hk

Contents

1. Methods S2
2. NMR Spectra S10
3. High-resolution mass spectra..........S30
4. Photophysical properties..........S33
5. X-ray crystallography data S35
6. Density functional theory (DFT) calculations S37
7. References S51

1. Methods

Synthesis

General: The reagents and starting materials employed were commercially available and used without any further purification or made following reported methods as indicated. Unless otherwise noted, all reaction were performed with dry solvents under an atmosphere of nitrogen in dried glassware with standard vacuum-line techniques. Anhydrous and oxygen-free toluene, THF and dichloromethane were purified by an Advanced Technology Pure-Solv PS-MD-4 system. Gel permeation chromatography (GPC) were performed on a JAI LC-9160 II NEXT automatic recycling preparative HPLC system with a UV/vis detector. ^{1}H and ^{13}C NMR spectra were recorded on Brucker AVANCE III 400 NMR or AVANCE III HD 500 NMR spectrometers. Abbreviations: *s* = singlet, *d* = doublet, *t* = triplet, *m* = multiplet, *br* = broad. Chemical shift values (δ) are expressed in parts per million using residual solvent protons as an internal standard (^{1}H NMR, δ_H = 5.32 for CD_2Cl_2, δ_H = 5.98 for $C_2D_2Cl_4$, δ_H = 7.26 for $CDCl_3$; ^{13}C NMR, δ_C = 53.84 for CD_2Cl_2, δ_C = 77.16 for $CDCl_3$). Mass spectra were recorded on a Bruker Autoflex speed MALDI-TOF spectrometer. Melting points, without correction, were measured using a Nikon Polarized Light Microscope ECLIPSE 50i POL equipped with an INTEC HCS302 heating stage.

A standard photochemical reaction vessel with a double-walled quartz immersion well was used for the photochemical reactions. A 500 W high-pressure mercury vapor lamp, as the light source, was placed in the immersion quartz well, and a circulating chiller was used to provide cooling water to the quartz immersion well.

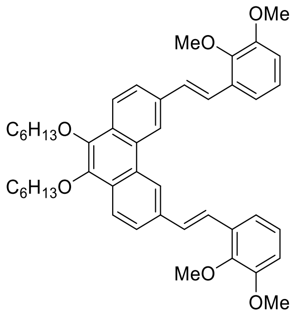

9,10-Dihexoxy-3,6-bis[(1*E*)-2-(2,3-dimethoxyphenyl)ethenyl]phenanthrene (**7a**): A mixture of 3,6-dibromo-9,10-bis(hexyloxy)phenanthrene **5** [1] (1.0 g, 1.9 mmol), 2,3-dimethoxystyrene **6** [2] (673 mg, 4.1 mmol), potassium carbonate (309 mg, 2.2 mmol), tetrabutylammonium bromide (721 mg, 2.2 mmol), palladium acetate (42 mg, 0.19 mmol) in DMF (60 mL) was heated in a Schlenk flask under an atmosphere of N_2 at 140°C for 16 h. After passing a pad of celite, the resulting solution was extracted with CH_2Cl_2, washed with brine, dried over $MgSO_4$, and concentrated under a reduced pressure. The residue was purified by column chromatography on silica gel with hexane/ethyl acetate 10/1 (V/V) as eluent to afford **7a** (635 mg, 49%) as yellow oil. ^{1}H NMR (400 MHz, $CDCl_3$): δ = 8.71 (s, 2H), 8.23 (d, *J* = 8.6 Hz, 2H), 7.94 (d, *J* = 8.6 Hz, 2H), 7.67 (d, *J* = 16.4 Hz, 2H), 7.44 (d, *J* = 16.4 Hz, 2H), 7.37 (d, *J* = 7.9 Hz, 2H), 7.12 (t, *J* = 8.0 Hz, 2H), 6.88 (d, *J* = 7.9 Hz, 2H), 4.23 (t, *J* = 6.7 Hz, 4H), 3.92 (s, 6H), 3.91 (s, 6H), 1.96-1.89 (m, 4H), 1.62-1.55 (m, 4H), 1.43-1.37 (m, 8H), 0.96-0.92 (m, 6H); ^{13}C NMR (100 MHz, $CDCl_3$) δ = 153.2, 147.1, 143.6, 135.1, 131.8, 130.4, 129.5, 128.9, 124.5, 124.3, 123.2, 122.9, 122.0, 118.0, 111.5, 73.9, 61.3, 55.9, 31.9, 30.6, 26.1, 22.8, 14.2. HRMS (MALDI-TOF): *m/z* calcd. for $C_{46}H_{54}O_6$ ($[M]^+$): 702.3915, found: 702.3928.

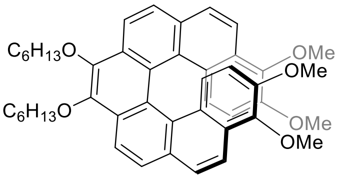

9,10-Dihexoxy-3,4,15,16-tetramethoxy[7]helicene (**8a**): In a 500 mL photochemical reactor, a stirred solution of compound **7a** (635 mg, 0.9 mmol), propylene oxide (13 mL, 180.9 mmol) in toluene (450 mL) was degassed with N_2 for 20 minutes, then I_2 (550 mg, 2.0 mmol) was added to photochemical reactor. The reaction mixture was irradiated with a 500 W high-pressure mercury vapor lamp for 1.5 h, and then was washed with small portions of saturated aqueous solution of $Na_2S_2O_3$ until the color of iodine had disappeared. The organic layer was then dried over Na_2SO_4 and all volatiles were removed in vacuo. The residue was purified by column chromatography on silica gel with hexane/ethyl acetate 8/1 (V/V) as eluent to afford compound **8a** (380 mg, 60%) as yellow solids. mp: 144.6–145.2 °C. ^{1}H NMR (400 MHz, $CDCl_3$): δ = 8.36 (d, J = 8.4 Hz, 2H), 7.90 (d, J = 8.4 Hz, 2H), 7.83 (d, J = 8.8 Hz, 2H), 7.73 (d, J = 8.8 Hz, 2H), 6.96 (d, J = 9.2 Hz, 2H), 6.15 (d, J = 9.3 Hz, 2H), 4.53-4.45 (m, 2H), 4.33-4.26 (m, 2H), 3.79 (s, 6H), 3.73 (s, 6H), 2.08-2.00 (m, 4H), 1.73-1.63 (m, 4H), 1.52-1.40 (m, 8H), 0.98 (t, J = 6.8 Hz, 6H); ^{13}C NMR (100 MHz, $CDCl_3$) δ = 148.0, 143.9, 141.9, 129.3, 129.0, 128.3, 127.4, 127.3, 126.7, 125.2, 123.2, 121.2, 120.1, 120.1, 111.1, 74.1, 61.3, 56.4, 31.9, 30.7, 26.1, 22.8, 14.2. HRMS (MALDI-TOF): *m/z* calcd. for $C_{46}H_{50}O_6$ ($[M]^+$): 698.3602, found: 698.3626.

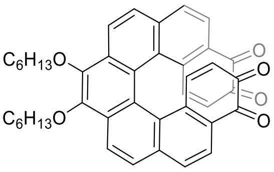

9,10-Dihexoxy-3,4,15,16-tetraoxo[7]helicene (**9a**): To a stirred solution of compound **8a** (160 mg, 0.23 mmol) in dry CH_2Cl_2 (15 mL) under N_2 was added a solution of 1M BBr_3 in CH_2Cl_2 (0.46 mL, 0.46 mmol) at −84 °C. The reaction mixture was slowly warmed to room temperature and stirred overnight. The black mixture was poured into ice water. The organic yellow layer was further washed with brine and dried with anhydrous $MgSO_4$ and concentrated under a reduced pressure. Flash chromatography (ethyl acetate) afforded 9,10-dihexoxy-3,4,15,16-tetrahydroxy[7]helicene as brown solid, used in the next step without further purifications. *(Note: The accurate amount of BBr_3 is crucial, BBr_3 less than 2 eq. gave partial demethylated products, while 4.0 eq. BBr_3 afforded over dealkylation product with two n-hexyloxyl groups converted to hydroxyl groups.)*

To a stirred solution of above 9,10-dihexoxy-3,4,15,16-tetrahydroxy[7]helicene in Acetonitrile (15 mL) was added pyridinium dichromate (386 mg, 1.0 mmol) at 0 °C and stirred for 30 minutes. The red mixture was filtered, filtrate was collected under a reduced pressure, and purified by column chromatography on silica gel with hexane/CH_2Cl_2/ethyl acetate 1/1/1 (V/V/V) as eluent to afford compound **9a** (65 mg, 45%) as red solids. mp: 175.9–176.3 °C. ^{1}H NMR (400 MHz, $CDCl_3$): δ = 8.56 (d, J = 8.7 Hz, 2H), 8.19 (d, J = 8.1 Hz, 2H), 8.08 (d, J = 8.2 Hz, 2H), 8.02 (d, J = 8.8 Hz, 2H), 6.15 (d, J = 10.5 Hz, 2H), 5.38 (d, J = 10.5 Hz, 2H), 4.59-4.51 (m, 2H), 4.28-4.21 (m, 2H), 2.06-1.96 (m, 4H), 1.69-1.59 (m, 4H), 1.48-1.38 (m, 8H), 0.95 (t, J = 6.9 Hz, 6H); ^{13}C NMR (100 MHz, $CDCl_3$) δ = 180.0, 178.3, 146.2, 141.4, 136.8, 131.6, 130.5, 129.9, 129.7, 128.0, 127.9, 126.6, 124.5, 124.2, 123.4, 74.7, 31.8, 30.6, 26.0, 22.8, 14.2. HRMS (APCI): *m/z* calcd. for $C_{42}H_{38}O_6$ ($[M+H]^+$): 639.27412, found: 639.27366.

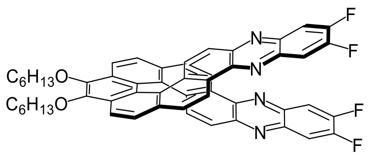

Compound **3a**: A solution of **9a** (20 mg, 0.03 mmol) and 1,2-diamino-4,5-difluorobenzene (20 mg, 0.14 mmol) in acetic acid (5 mL) was stirred at 70 °C under an atomosphere of N_2 for 2h. Then the reaction mixture was added H_2O, the yellow precipitate came out and was filtered. The residue was purified by column chromatography on silica gel with hexane/dichloromethane/ethyl acetate 4/1/1 (V/V/V) as eluent to afford **3a** (20 mg, 74%) as yellow solids. mp: not melt when heated up to 300 °C. ^1H NMR (400 MHz, CD_2Cl_2): δ = 8.72 (d, J = 8.3 Hz, 2H), 8.57 (d, J = 8.4 Hz, 2H), 8.14 (d, J = 8.2 Hz, 2H), 8.13 (d, J = 8.3 Hz, 2H), 7.83 (t, J = 9.3 Hz, 2H), 7.17 (t, J = 9.3 Hz, 2H), 6.95 (d, J = 9.5 Hz, 2H), 6.68 (d, J = 9.5 Hz, 2H), 4.62-4.54 (m, 2H), 4.38-4.30 (m, 2H), 2.12-2.02 (m, 4H), 1.75-1.05 (m, 4H), 1.53-1.42 (m, 8H), 0.99 (t, J = 6.8 Hz, 6H); ^{13}C NMR (100 MHz, CD_2Cl_2) δ = 145.4, 142.5, 141.4, 139.9, 139.8, 138.6, 138.5, 133.2, 129.6, 129.5, 129.1, 129.0, 128.5, 127.8, 126.8, 123.2, 123.0, 122.9, 122.7, 114.1, 113.9, 113.8, 113.6, 74.7, 32.2, 31.0, 26.4, 23.1, 14.3. HRMS (MALDI-TOF): *m/z* calcd. for $C_{54}H_{42}N_2O_2F_4$ ($[M]^+$): 854.3238, found: 854.3238.

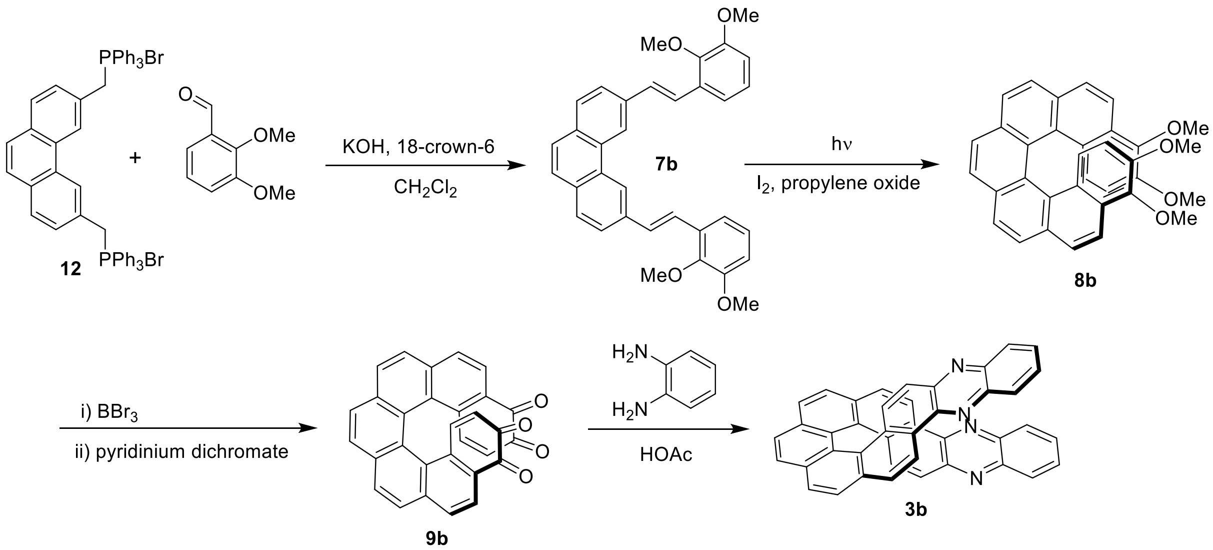

Scheme S1. Synthesis of compound **3b**.

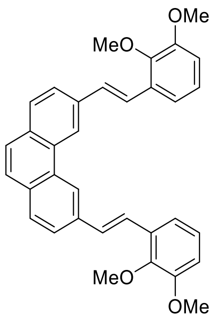

3,6-Bis[(1*E*)-2-(2,3-dimethoxyphenyl)ethenyl]phenanthrene (**7b**): A mixture of **12** (1.71 g, 1.93 mmol), 2,3-dimethoxybenzaldehyde (799 mg, 4.81 mmol), and 18-crown-6 (122 mg, 0.46 mmol) in CH_2Cl_2 (70 ml) was cooled to −84 °C and then pulverized potassium hydroxide (485 mg, 8.66 mmol) was added. The mixture was stirred at −84 °C for 1h, slowly warmed up to 0°C, and stirred for another 3h. The resulting mixture was warmed up to room temperature,

diluted with CH_2Cl_2, filtrated and quenched with an aqueous NH_4Cl solution. The organic layer was washed with water, brine, dried with Na_2SO_4, and concentrated. The obtained residue was further purified by silica gel column chromatography with hexane/EA = 8/1 as an eluent to get **7b** (674 mg, 69 %) as colorless solids. mp: 205.2–205.8 °C. ^{1}H NMR (500 MHz, $CDCl_3$): δ = 8.30 (s, 2H), 7.62 (d, J = 8.3 Hz, 2H), 7.59 (s, 2H), 7.43 (d, J = 8.2 Hz, 2H), 6.87-6.80 (m, 5H), 3.92 (s, 6H), 3.90 (s, 6H). ^{13}C NMR (126 MHz, CD_2Cl_2) δ = 153.1, 147.3, 135.4, 132.1, 131.3, 131.2, 130.2, 128.1, 127.5, 126.7, 126.3, 123.9, 123.4, 122.2, 111.6, 61.0, 55.9. HRMS (APCI): *m/z* calcd. for $C_{34}H_{30}O_4$ ([M+H]$^+$): 503.22169, found: 503.22109.

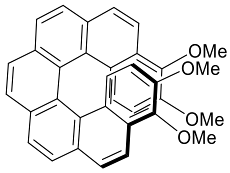

3,4,15,16-Tetramethoxy[7]helicene (**8b**): In a 500ml photochemical reactor, a stirred solution of compound **7b** (316 mg, 0.63 mmol), I_2 (328 mg, 1.29 mmol), and propylene oxide (8.8 ml, 126.0 mmol) in toluene (400 ml) was degassed with N_2 for 20 min. After irradiation with a 500 W high-pressure mercury vapor lamp for 2h, the reaction mixture was treated with an aqueous $Na_2S_2O_3$ solution to remove the unreacted I_2. The organic layer was concentrated in vacuo to afford brown solids, which was roughly washed with MeOH, and then filtered. The obtained residue was further purified by silica gel column chromatography with hexane/EA = 8/1 as an eluent to get **8b** (180 mg, 58 %) as yellow solid**.** mp: 235.3–235.8 °C. ^{1}H NMR (500 MHz, $CDCl_3$): δ = 7.99 (s, 2H), 7.93 (d, J = 8.1 Hz, 2H), 7.88 (d, J = 8.5 Hz, 2H), 7.86 (d, J = 9.2 Hz, 2H), 7.74 (d, J = 8.8 Hz, 2H), 6.96 (d, J = 9.3 Hz, 2H), 6.15 (d, J = 9.3 Hz, 2H), 3.79 (s, 6H), 3.72 (s, 6H); ^{13}C NMR (126 MHz, $CDCl_3$) δ = 148.0, 141.9, 132.3, 129.9, 128.3, 127.3, 127.3, 126.9, 126.7, 126.4, 125.3, 125.2, 121.0, 120.5, 111.3, 61.4, 56.4. HRMS (APCI): *m/z* calcd. for $C_{34}H_{26}O_4$ ([M+H]$^+$): 499.19039, found: 499.18969.

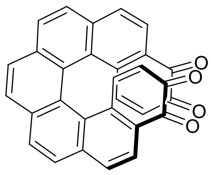

3,4,15,16-Tetraoxo[7]helicene (**9b**): In an oven-dried Schlenk flask was placed **8b** (60 mg, 0.12 mmol) in anhydrous CH_2Cl_2 (10 ml) under nitrogen atmosphere. The mixture was cooled to −84 °C, and a 1 M solution of BBr_3 in CH_2Cl_2 (1.2 ml, 1.2 mmol) was added. The resulting black solution was stirred at −84 °C for 1h, then allowed to warm to room temperature and stirred for 2h. The mixture was poured into ice water, and extracted with CH_2Cl_2. The organic layer was further washed with brine and dried with anhydrous $MgSO_4$, and concentrated under a reduced pressure to give crude 3,4,15,16-tetrahydroxy[7]helicene as reddish black solid. To a stirred solution of the above 3,4,15,16-tetrahydroxy[7]helicene in acetonitrile (10 mL) was added pyridinium dichromate (225 mg, 0.6 mmol) at 0 °C and stirred for 30 minutes. The red mixture was filtered, filtrate was collected under a reduced pressure, and purified by column chromatography on silica gel with hexane/ethyl acetate 1/1 (V/V) as eluent to afford compound **9b** (15 mg, 30 %) as red solids. mp: not melt when heated up to 300 °C. ^{1}H NMR (400 MHz,

$CDCl_3$): δ = 8.24 (s, 2H), 8.23 (d, J = 7.5 Hz, 2H), 8.19 (d, J = 8.1 Hz, 2H), 8.11 (d, J = 8.1 Hz, 2H), 8.04 (d, J = 8.5 Hz, 2H), 6.18 (d, J = 10.5 Hz, 2H), 5.39 (d, J = 10.5 Hz, 2H). ^{13}C NMR (100 MHz, $CDCl_3$) δ = 179.9, 178.3, 141.0, 137.2, 133.0, 131.7, 130.5, 130.3, 129.9, 129.0, 128.0, 127.9, 127.0, 126.1, 124.4. HRMS (APCI): *m/z* calcd. for $C_{30}H_{14}O_4$ ($[M+H]^+$): 439.09649, found: 439.09604.

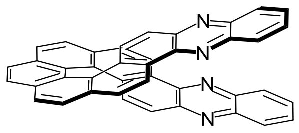

Compound **3b**: A solution of compound **9b** (12 mg, 0.027 mmol) and *o*-phenylenediamine (48 mg, 0.4 mmol) in acetic acid (3 mL) was stirred at 70 °C under an atomosphere of N_2 overnight, and cooled to room temperature, then quenched with H_2O and extracted with CH_2Cl_2. The extracts were combined, washed with brine, dried over $MgSO_4$, and concentrated under a reduced pressure. The residue was purified by column chromatography on silica gel with hexane/dichloromethane/ethyl acetate 10/5/3 to 2/1/1 (V/V/V) as eluent to afford **3b** (12 mg, 75 %) as yellow solids. mp: not melt when heated up to 300 °C. ^1H NMR (400 MHz, CD_2Cl_2): δ = 8.94 (d, J = 8.4 Hz, 2H), 8.26-8.17 (m, 8H), 8.04 (d, J = 8.5 Hz, 2H), 7.65 (t, J = 6.6 Hz, 2H), 7.47-7.39 (m, 4H), 7.04 (d, J = 9.6 Hz, 2H), 8.94 (d, J = 9.6 Hz, 2H). Due to the poor solubility, clear ^{13}C NMR spectrum could not be recorded after overnight measurements. HRMS (MALDI-TOF): *m/z* calcd. For $C_{42}H_{22}N_4$ ($[M+H]^+$): 583.1917, found: 583.1914.

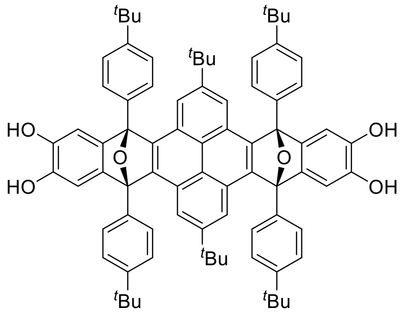

Compound **4:** To a solution of **10** [3] (54 mg, 0.048 mmol) in THF (15 ml) was added a solution of $Na_2S_2O_4$ (99 mg. 0.569 mmol) in H_2O (5 ml) at room temperature under N_2 and stirred for 10 minutes. The reaction mixture was extracted with ethyl acetate, washed with brine, dried over $MgSO_4$, and concentrated under a reduced pressure. The crude compound **4** (50 mg, 100%) was obtained as red solids used in the next step without further purifications due to its sensitivity to air. ^1H NMR (500 MHz, CD_2Cl_2) δ = 7.97 (d, J = 5.4 Hz, 8H), 7.61 (s, 4H), 7.52 (d, J = 6.5 Hz, 8H), 7.19 (s, 4H), 5.5 (br, 4H), 1.34 (s, 36H), 0.93 (s, 18H). HRMS (APCI): *m/z* calcd. for $C_{80}H_{82}O_6$ ($[M+H]^+$): 1139.6184, found: 1139.6171.

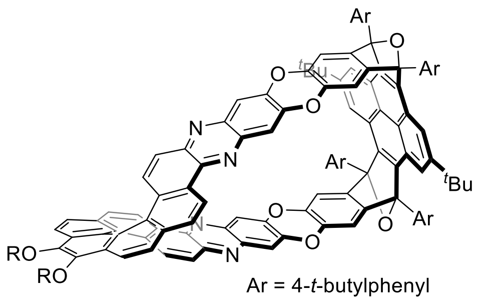

Compound **2:** A mixture of **3a** (35 mg, 0.041 mmol), compound **4** (51 mg, 0.045 mmol), pulverized potassium carbonate (50 mg, 0.36 mmol) in degassed DMF (40 mL) was heated in a Schlenk flask under an atmosphere of N_2 at 100°C for 2 days. The resulting black solution was extracted with CH_2Cl_2, washed with brine, dried over $MgSO_4$, and concentrated under a reduced pressure. The residue was purified by column chromatography on silica gel with hexane/CH_2Cl_2 1/2 (V/V) as eluent to afford **2** (9.5 mg, 12%) as yellow solids. mp: not melt when heated up to 300 °C. ^{1}H NMR (400 MHz, CD_2Cl_2 + CS_2) δ = 9.09 (d, J = 8.6 Hz, 2H), 8.58 (d, J = 8.6 Hz, 2H), 8.22 (d, J = 8.6 Hz, 2H), 8.16 (d, J = 8.6 Hz, 2H), 8.03 (d, J = 7.6 Hz, 8H), 7.68 (s, 4H), 7.66 (d, J = 7.7 Hz, 8H), 7.12 (s, 2H), 7.12 (s, 2H), 7.10 (s, 2H), 7.07 (s, 2H), 7.06 (d, J = 9.7 Hz, 2H), 6.62 (d, J = 9.7 Hz, 2H), 4.59-4.52 (m, 2H), 4.34-4.27 (m, 2H), 2.10-2.02 (m, 4H), 1.75-1.66 (m, 4H), 1.46 (s, 36H), 1.48-1.46 (m, 8H), 1.02 (m, 18H), 1.00-0.98 (m, 6H); ^{13}C NMR (126 MHz, CD_2Cl_2 + CS_2) δ = 152.8, 150.3, 150.0, 148.3, 148.0, 147.9, 145.5, 145.1, 145.1, 141.3, 141.3, 140.3, 138.1, 137.7, 132.8, 132.4, 132.3, 130.4, 129.7, 129.6, 128.8, 128.5, 128.3, 128.3, 127.1, 127.0, 126.2, 123.7, 123.6, 123.5, 123.1, 122.2, 119.5, 112.6, 112.0, 111.9, 93.4, 93.3, 74.6, 35.1, 35.0, 32.6, 31.7, 31.5, 31.3, 26.9, 23.7, 14.9. HRMS (MALDI-TOF): *m/z* calcd. for $C_{134}H_{120}N_4O_8$ ($[M]^+$): 1913.9134, found: 1913.9115.

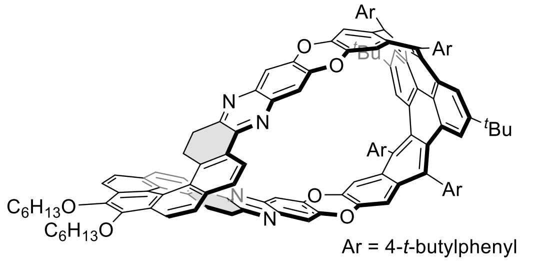

Compound **11**: To a solution of **2** (6 mg, 0.003 mmol, 1 equiv) and NaI (93 mg, 0.627 mmol) in mixture of CH_2Cl_2 (6 ml) and acetonitrile (3 ml) was added TMSI (90 µl, 0.627 mmol) at room temperature under N_2 and stirred for 2 h. The resulting solution was concentrated under reduced pressure. The residue was purified by preparative thin layer chromatography (PTLC) of silica gel with hexane/CH_2Cl_2 1/2 (V/V) as eluent to afford 3 mg (53%) of **11** as a yellow solid, ^{1}H NMR (400 MHz, CD_2Cl_2) δ = 8.72 (d, J = 8.4 Hz, 2H), 8.39 (d, J = 8.6 Hz, 2H), 8.05 (d, J = 8.5 Hz, 2H), 8.01 (d, J = 8.6 Hz, 2H), 7.74 (dd, J = 7.8, 1.7 Hz, 4H), 7.62-7.60 (m, 4H), 7.45 (dd, J = 7.0, 1.7 Hz, 4H), 7.35 (s, 2H), 7.19 (s, 2H), 6.91-6.89 (m, 4H), 6.88 (s, 2H), 6.82 (s, 2H), 6.21-6.19 (m, 4H), 4.49-4.44 (m, 2H), 4.21-4.15 (m, 2H), 2.26-2.25 (m, 4H), 2.00-1.95 (m, 4H), 1.71-1.67 (m, 4H), 1.61-1.58 (m, 4H), 1.52 (s, 18H), 1.51 (s, 18H), 1.42-1.39 (m, 8H), 0.94-0.91 (m, 6H), 0.72 (s, 18H); ^{13}C NMR (101 MHz, CD_2Cl_2) δ = 153.09, 150.52, 149.36, 148.22, 147.13, 145.64, 145.14, 145.03, 144.73, 139.96, 139.57, 138.02, 137.89, 136.61, 136.56, 135.80, 134.05, 132.23, 132.15, 131.67, 131.14, 130.25, 130.00, 129.84, 129.79, 128.79, 128.38, 128.11, 127.53, 126.59, 126.32, 126.00, 125.93, 124.48, 124.33, 124.25, 124.06, 121.42, 114.96, 113.68, 113.27, 113.13, 74.56, 35.04, 34.46, 32.32, 32.15, 31.75, 31.44, 30.88, 30.19, 30.10, 29.91, 29.77, 29.72, 29.64, 27.57, 26.36, 23.08, 14.28, 14.24. HRMS (MALDI-TOF): *m/z* calcd. for $C_{134}H_{124}N_4O_6$ ($[M]^+$): 1885.9554, found: 1885.9299.

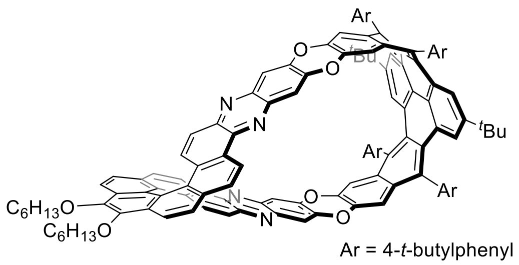

Ar = 4-*t*-butylphenyl

Compound **1**: A solution of **11** (4 mg, 0.002 mmol) and DDQ (7 mg, 0.028 mmol) in 1,2-dichloroethane (10 mL) was refluxed under N_2 for 2.5 h. After cooled to room temperature, the reaction mixture was treated with water and extracted with CH_2Cl_2, and concentrated under a reduced pressure. The residue was purified by preparative thin layer chromatography with hexane/CH_2Cl_2 3/2 (V/V) as eluent to afford 4 mg (100 %) of **1** as a yellow solid. ^1H NMR (500 MHz, CD_2Cl_2+CS_2) δ = 9.24 (d, J = 8.7 Hz, 2H), 8.57 (d, J = 8.6 Hz, 2H), 8.28 (d, J = 8.7 Hz, 2H), 8.22 (d, J = 8.6 Hz, 2H), 7.74 (dd, J = 7.8, 1.9 Hz, 4H), 7.61-7.59 (m, 4H), 7.49 (s, 2H), 7.48-7.44 (m, 4H), 7.36 (s, 2H), 7.19 (d, J = 9.9 Hz, 2H), 6.90-6.88 (m, 10H), 6.20-6.17 (m, 10H), 4.56-4.52 (m, 2H), 4.29-4.24 (m, 2H), 2.05-1.99 (m, 4H), 1.69-1.63 (m, 4H), 1.53 (s, 18H), 1.52 (s, 18H), 1.44-1.42 (m, 8H), 0.96-0.93 (m, 6H), 0.73 (s, 18H); ^{13}C NMR (101 MHz, CD_2Cl_2) δ = 150.52, 150.50, 150.26, 149.59, 145.03, 145.00, 144.95, 144.65, 142.05, 141.46, 141.16, 140.40, 137.87, 137.80, 136.60, 133.02, 132.28, 132.25, 131.63, 130.27, 130.15, 129.99, 129.81, 129.59, 129.48, 129.14, 128.66, 128.54, 128.15, 128.11, 126.58, 126.55, 126.30, 125.91, 125.88, 124.60, 124.20, 124.10, 123.56, 123.39, 122.02, 114.20, 113.35, 113.25, 74.59, 35.04, 34.45, 32.17, 31.75, 31.45, 30.91, 26.38, 23.10, 14.26. HRMS (MALDI-TOF): *m/z* calcd. for $C_{134}H_{120}N_4O_6$ ($[M]^+$): 1881.9236, found: 1881.9236.

Synthesis of compound **1** in enantiopure forms

Compound **3a** was resolved into two enantiomers with ee larger than 98% by Daicel Chiral Technologies (China) Co., Ltd. With Shimadzu LC-20AD_HPLC on a CHIRALPAK IB N-5 (IBN5CE-XA057) column using Hexane/CH_2Cl_2 = 70/30 (V/V) as the eluent. Following the synthetic procedures described above, (+)-**1** was synthesized from the second fraction of **3a** from the chiral HPLC, and (−)-**1** was synthesized from the first fraction of **3a** from the chiral HPLC. The specific rotation of (+)-**2** was measured as $[\alpha]_{546}^{25}$ = +921.1 (CH_2Cl_2), and that of (−)-**2** was measured as $[\alpha]_{546}^{25}$ = −961.1 (CH_2Cl_2). The specific rotation of (+)-**1** was measured as $[\alpha]_{546}^{25}$ = +1555.6 (CH_2Cl_2), and that of (−)-**1** was measured as $[\alpha]_{546}^{25}$ = −1555.6 (CH_2Cl_2) (the unit of specific rotation is $deg \cdot mL \cdot g^{-1} \cdot dm^{-1}$).

Attempted synthesis of N-containing Möbius nanobelts

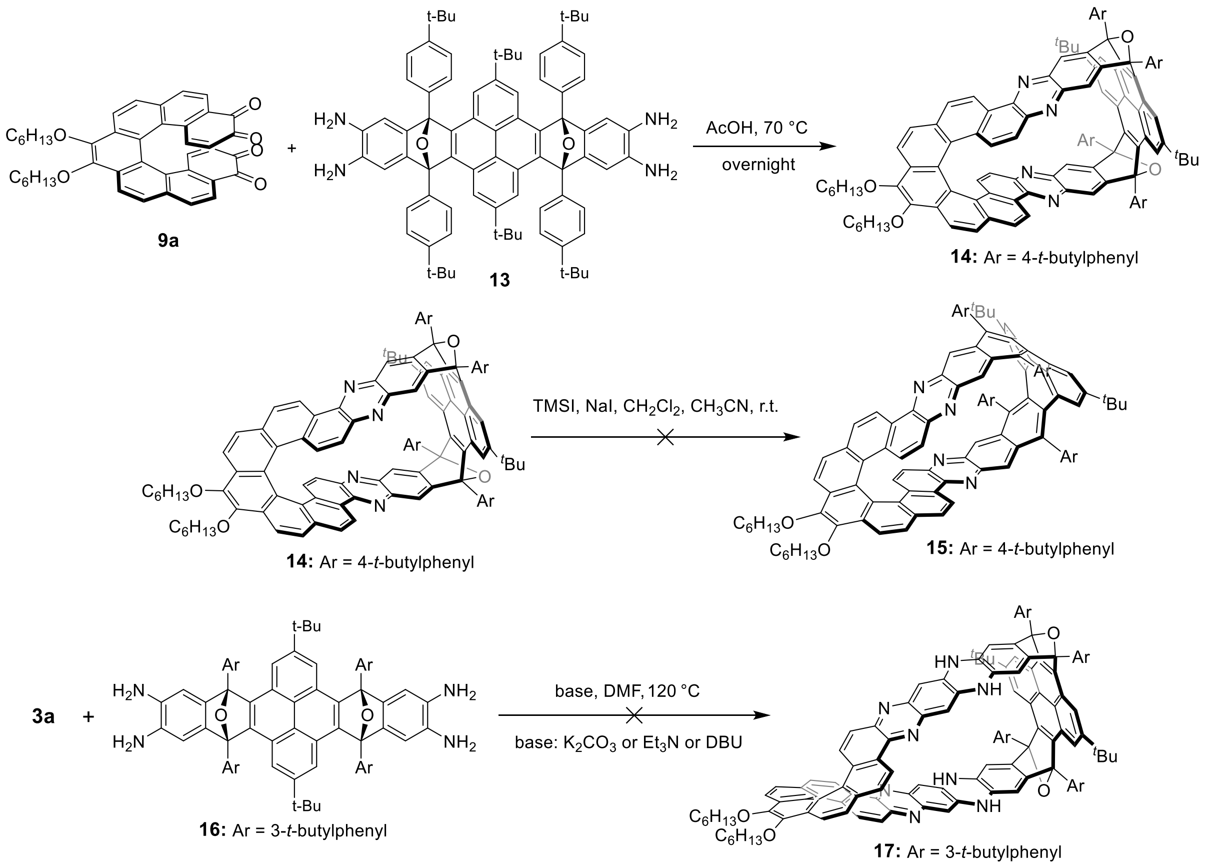

Scheme S2 Attempted reactions for synthesis of N-containing Möbius nanobelts

Compound **14**

A solution of **9a** (4 mg, 0.0062 mmol) and **13** [3] (7 mg, 0.0062 mmol) in acetic acid (5 mL) was stirred at 70 °C under N_2 overnight. The reaction system was cooled to room temperature, then quenched with H_2O, extracted with CH_2Cl_2, and concentrated under a reduced pressure. The residue was purified by preparative thin layer chromatography with hexane/CH_2Cl_2 1/4 (V/V) as eluent to afford compound **14** as yellow solid. ^{1}H NMR (400 MHz, CD_2Cl_2) δ = 9.09 (d, J = 8.5 Hz, 2H), 8.57 (d, J = 8.6 Hz, 2H), 8.22-8.18 (m, 10H), 7.83-7.69 (m, 14H), 7.13 (d, J = 9.7 Hz, 2H), 6.65 (d, J = 9.9 Hz, 2H), 4.57-4.54 (m, 2H), 4.30-4.25 (m, 2H), 2.06-1.99 (m, 4H), 1.68-1.65 (m, 4H), 1.46 (s, 18H), 1.45 (s, 18H), 1.29-1.25 (m, 8H), 1.03 (s, 18H), 0.97-0.93 (m, 6H). HRMS (MALDI-TOF): *m/z* calcd. for $C_{122}H_{116}N_4O_4$ ($[M]^+$): 1701.9024, found: 1701.8978.

2. NMR Spectra

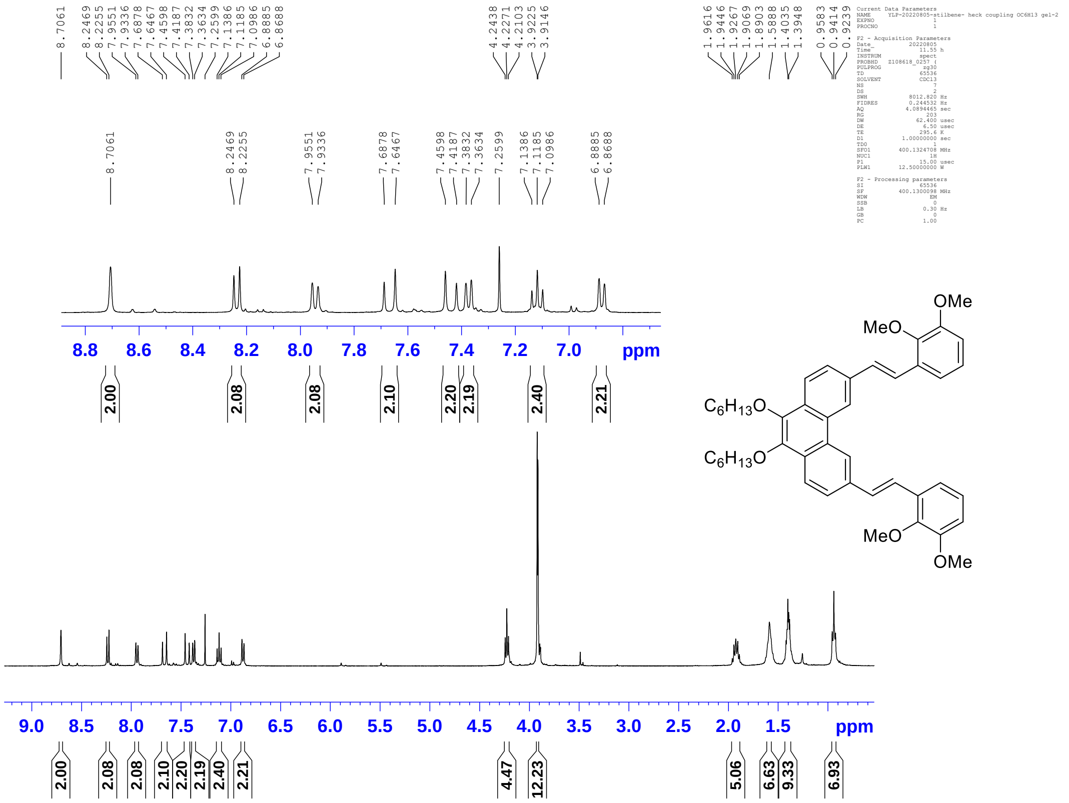

Figure S1. ^{1}H NMR spectrum of **7a** in $CDCl_3$ at 294K.

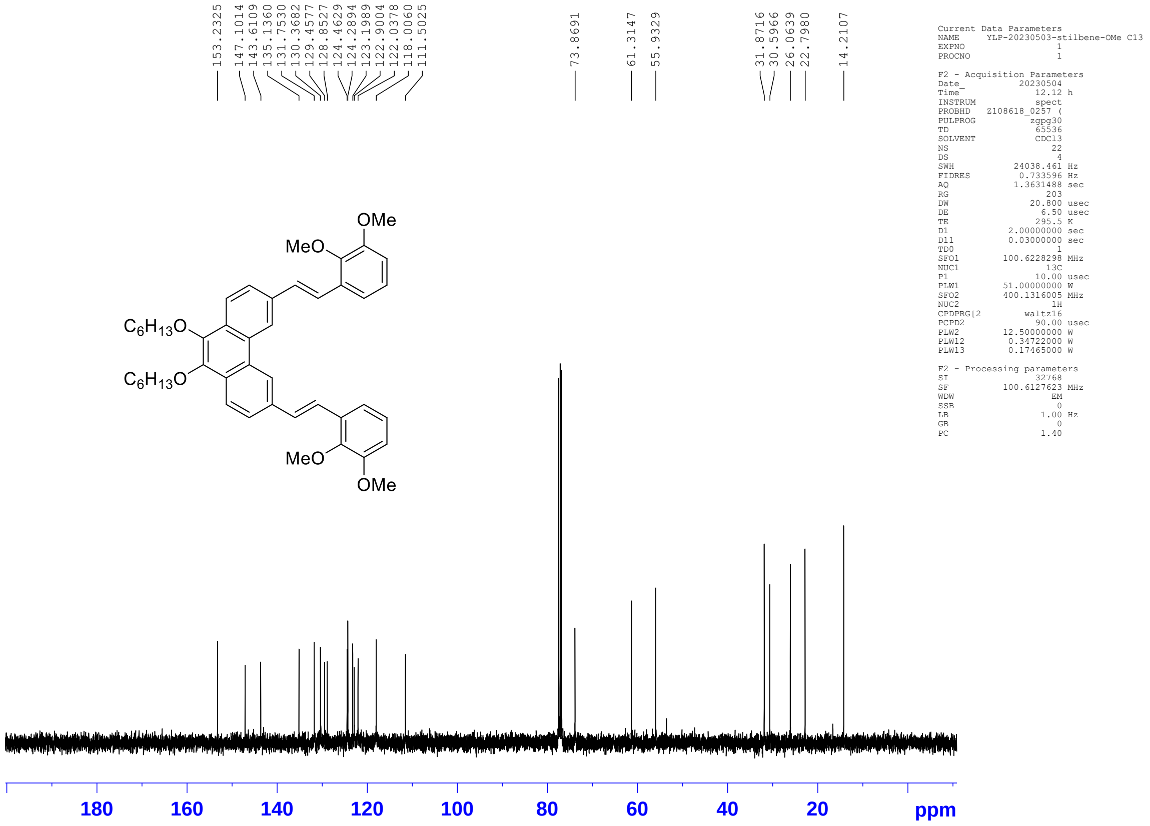

Figure S2. ^{13}C NMR spectrum of **7a** in $CDCl_3$ at 294K.

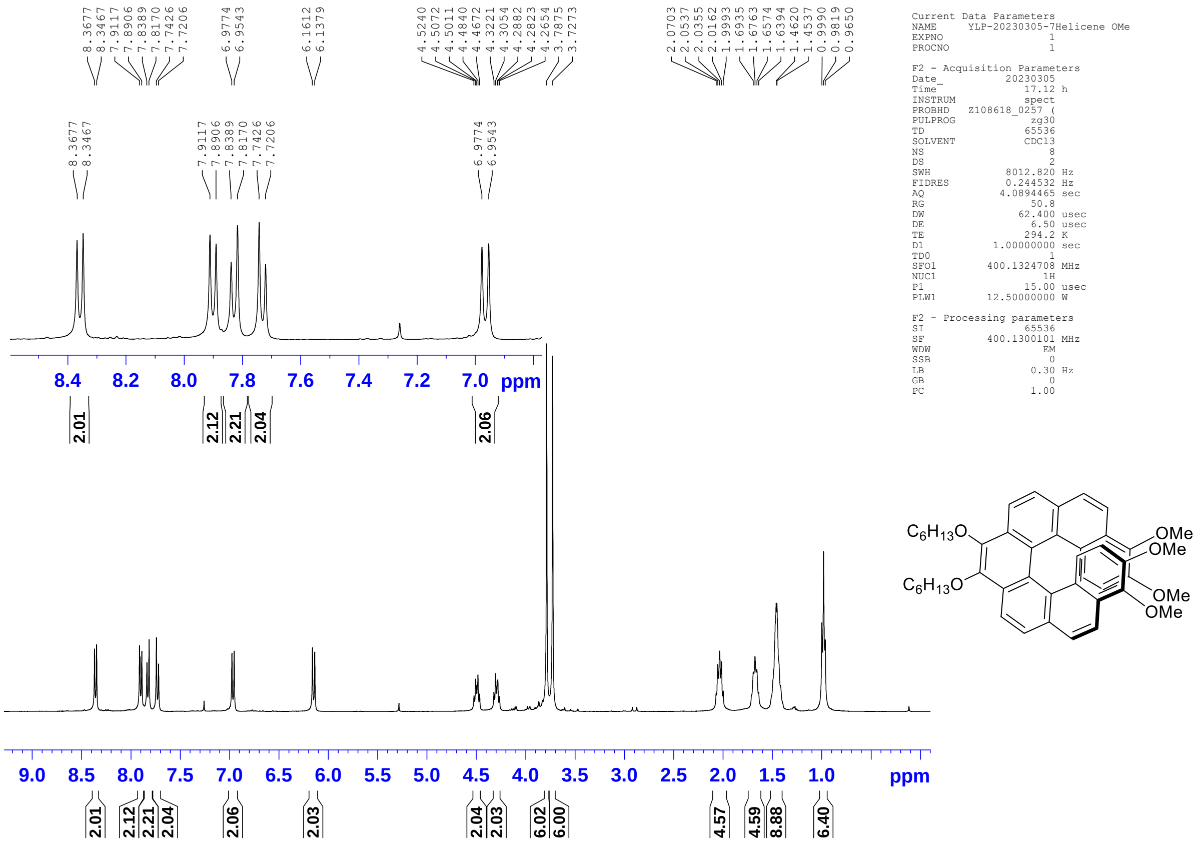

Figure S3. ^{1}H NMR spectrum of **8a** in $CDCl_3$ at 294K.

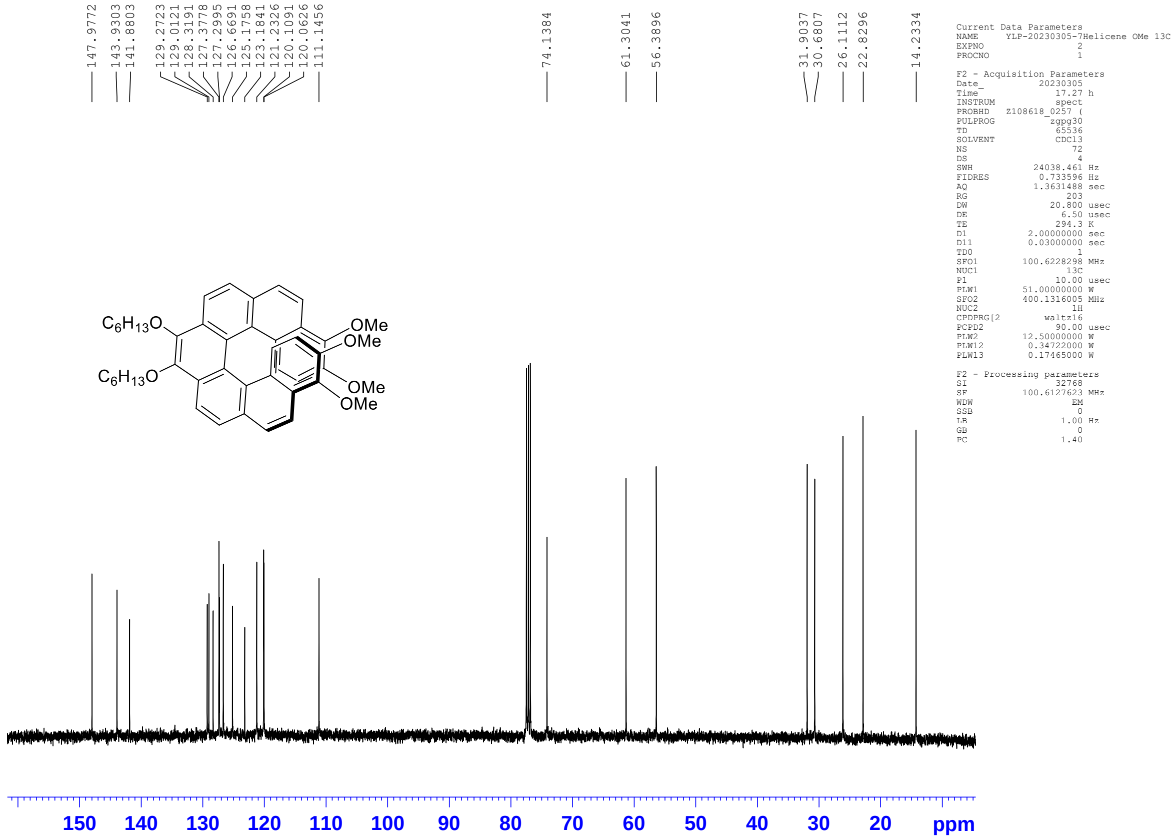

Figure S4. ^{13}C NMR spectrum of **8a** in $CDCl_3$ at 294K.

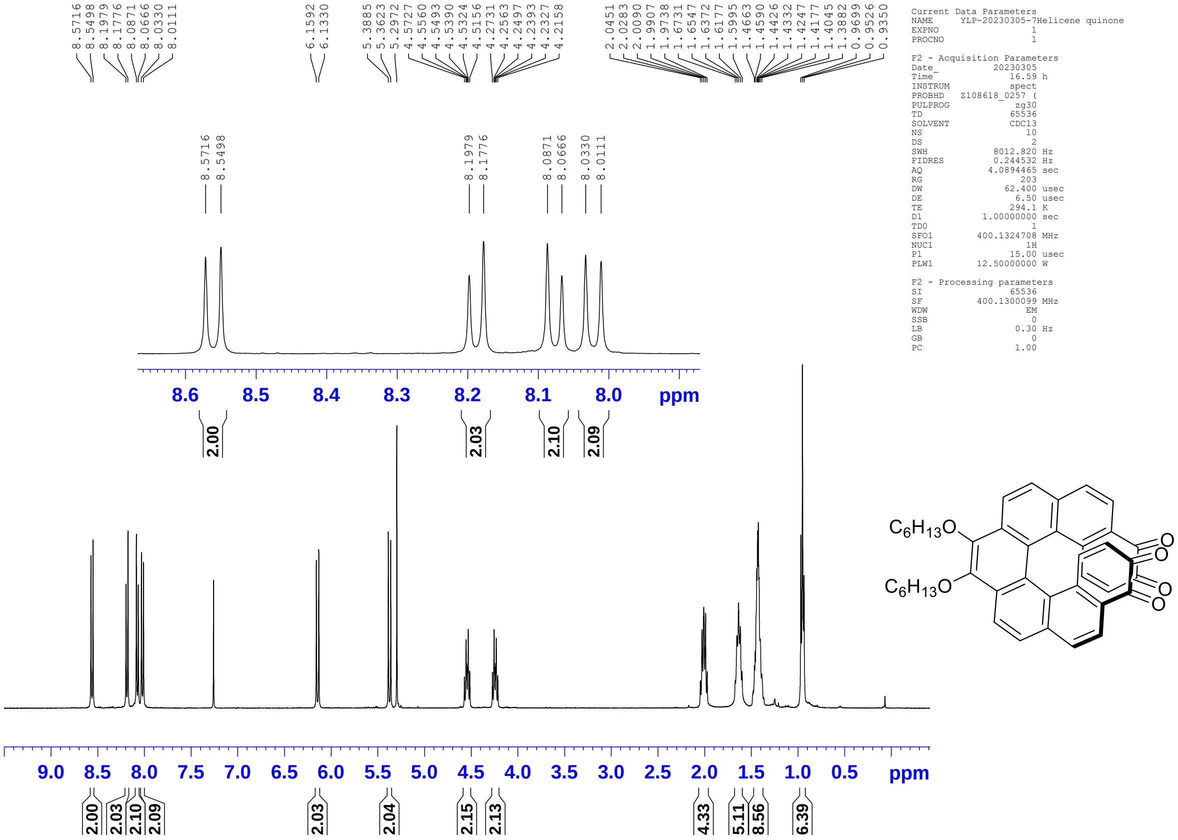

Figure S5. ^{1}H NMR spectrum of **9a** in $CDCl_3$ at 294K.

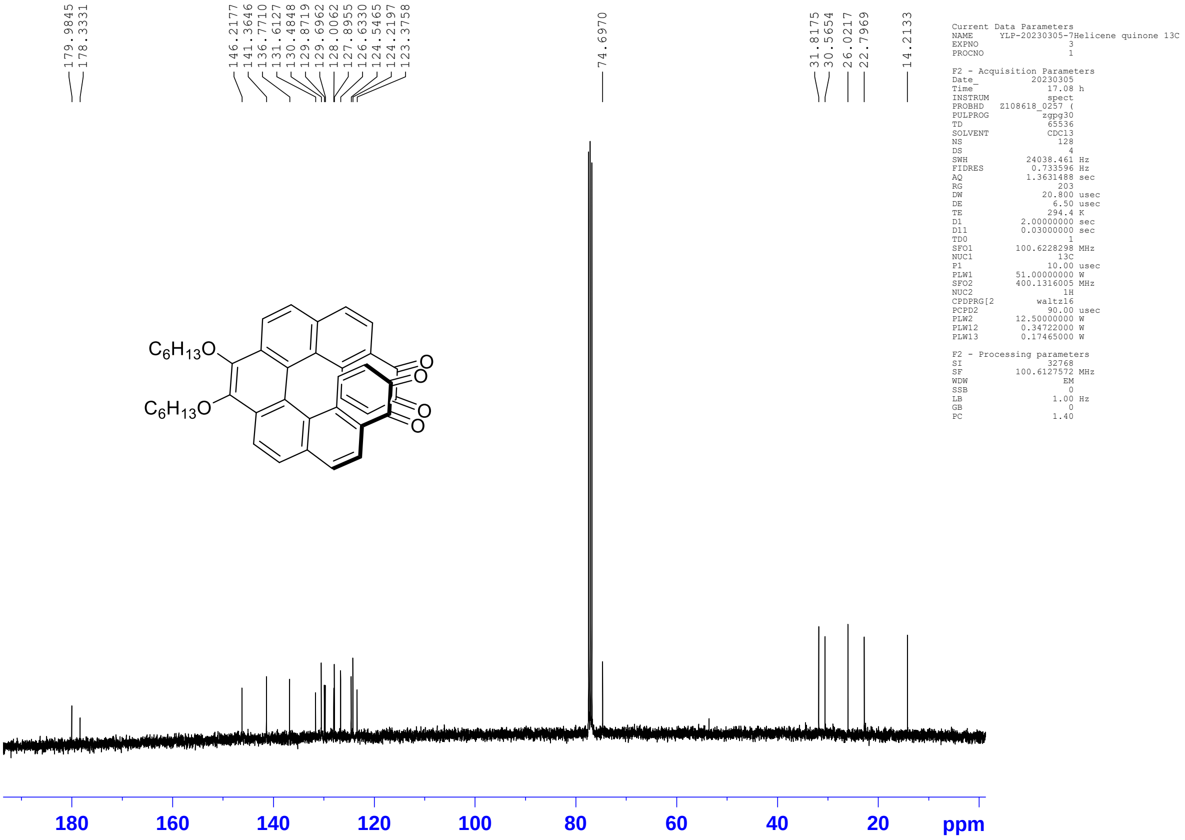

Figure S6. ^{13}C NMR spectrum of **9a** in $CDCl_3$ at 294K.

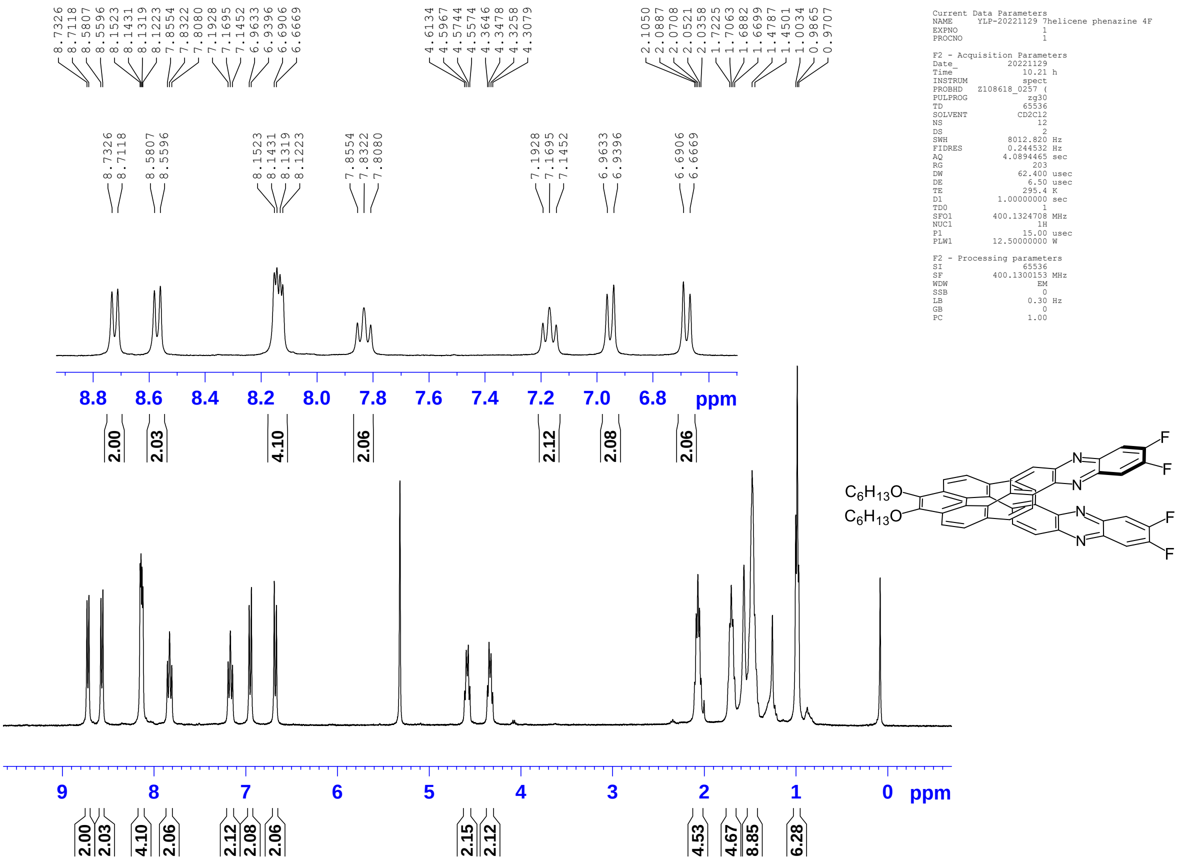

Figure S7. ^{1}H NMR spectrum of **3a** in CD_2Cl_2 at 294K.

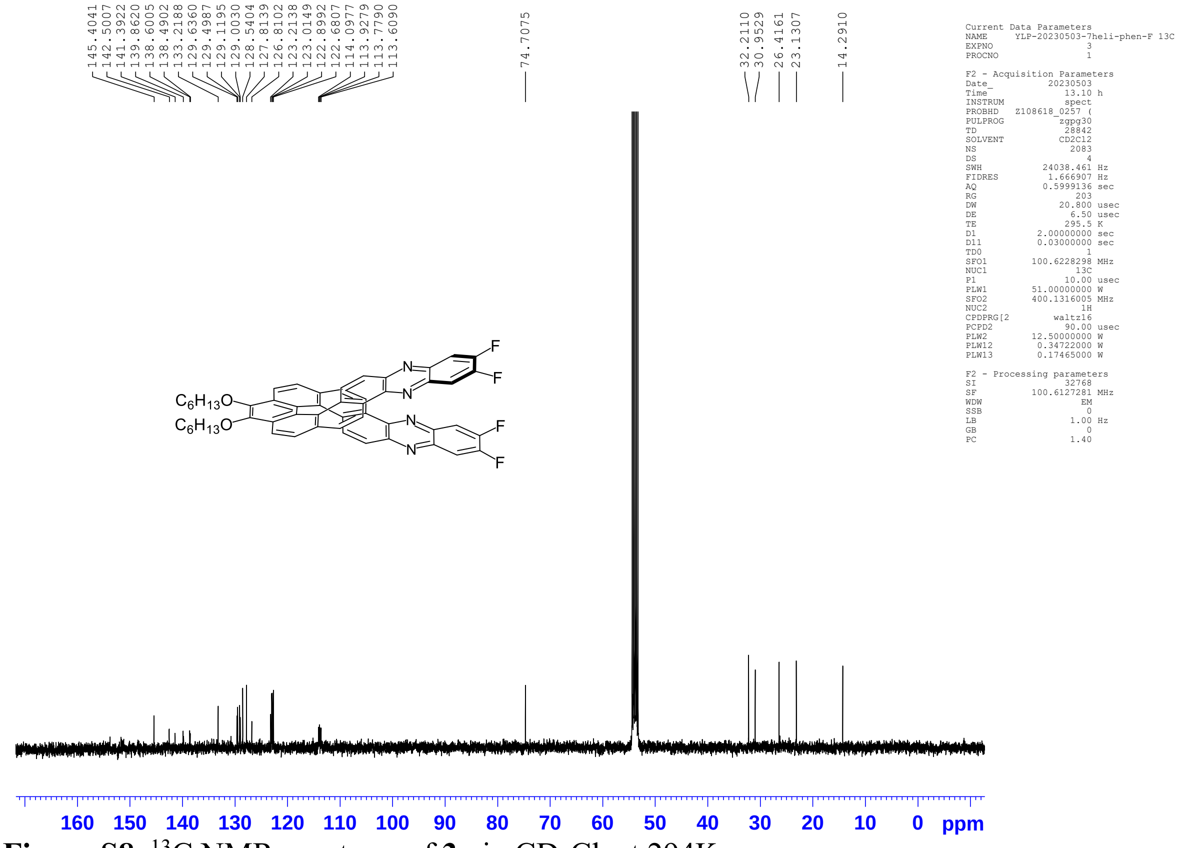

Figure S8. ^{13}C NMR spectrum of **3a** in CD_2Cl_2 at 294K.

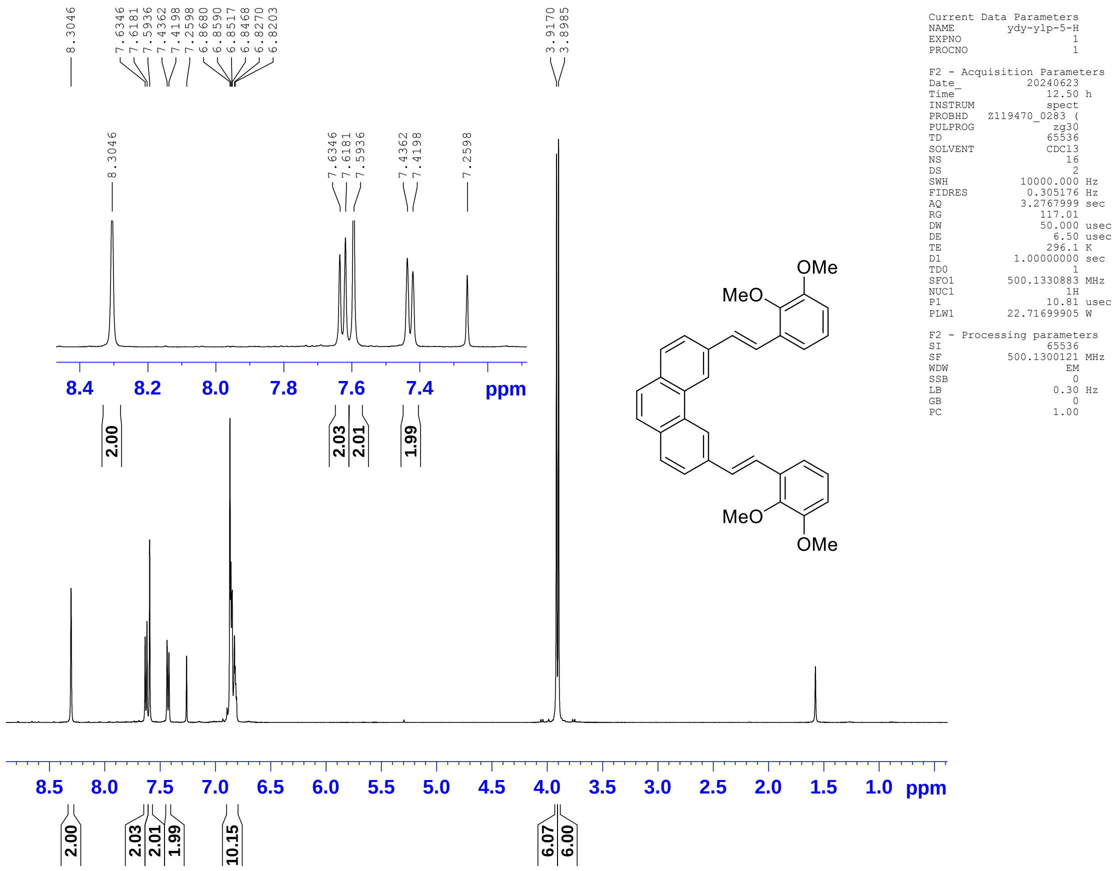

Figure S9. ^{1}H NMR spectrum of **7b** in $CDCl_3$ at 294K.

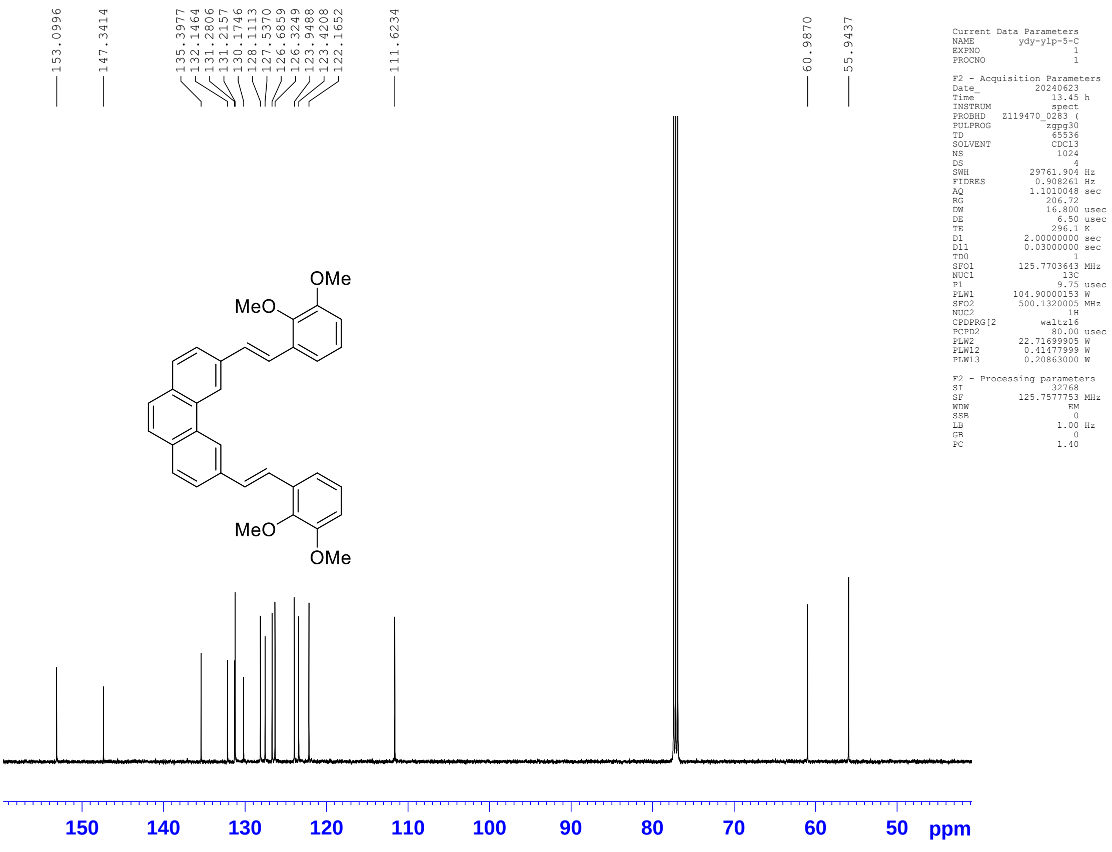

Figure S10. ^{13}C NMR spectrum of **7b** in $CDCl_3$ at 294K.

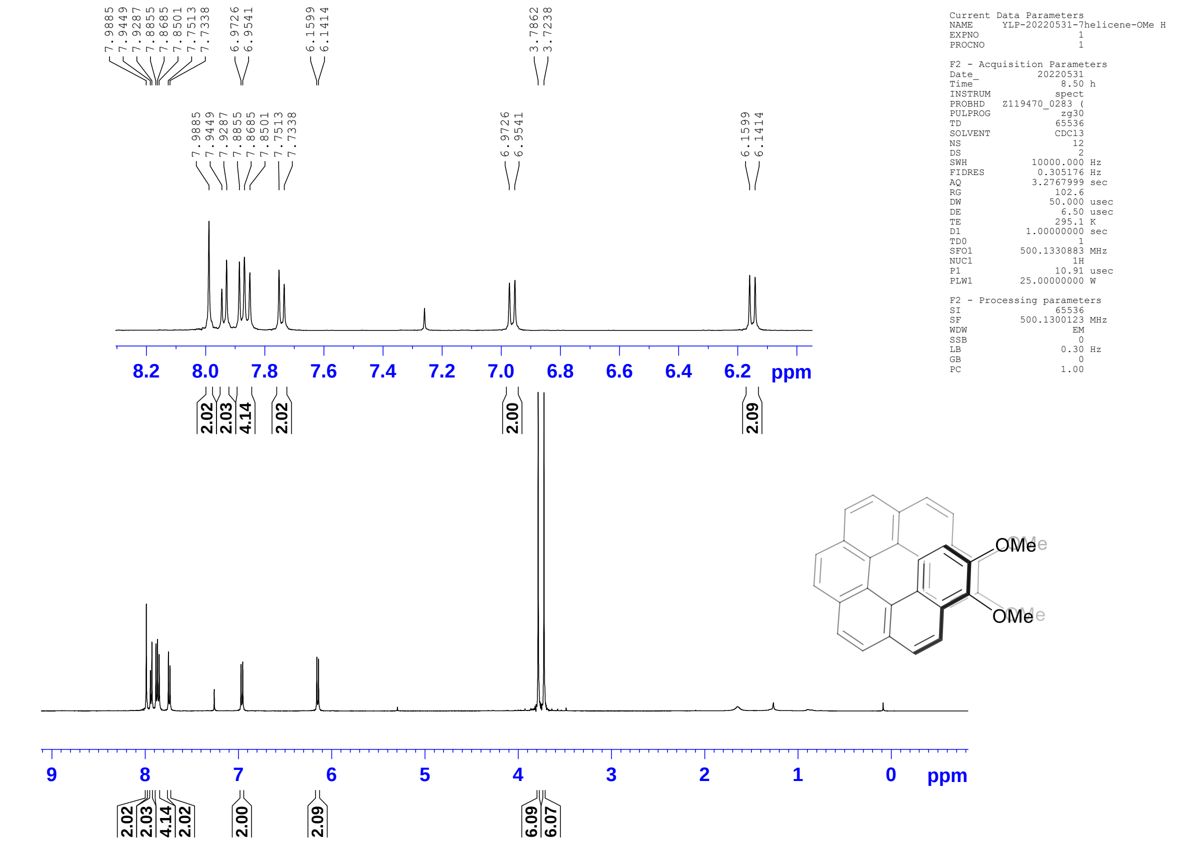

Figure S11. ^{1}H NMR spectrum of **8b** in $CDCl_3$ at 294K.

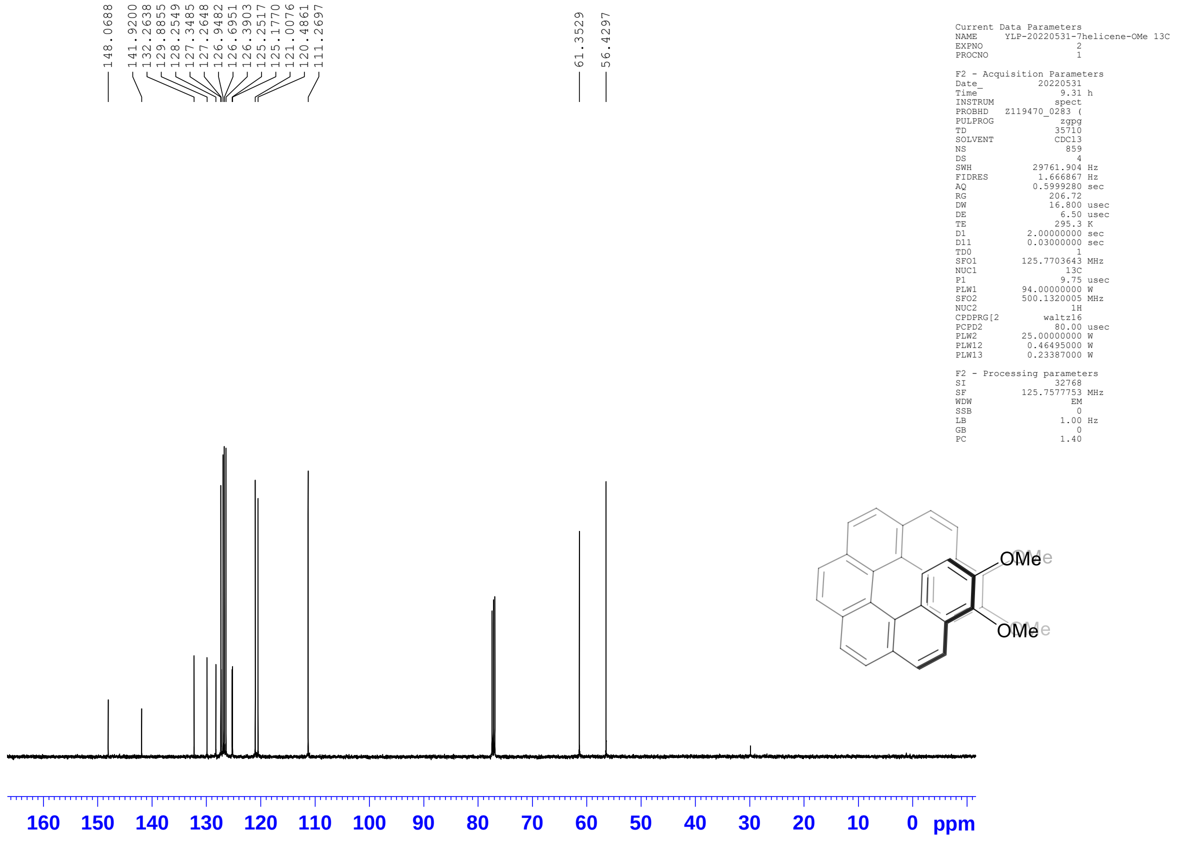

Figure S12. ^{13}C NMR spectrum of **8b** in $CDCl_3$ at 294K.

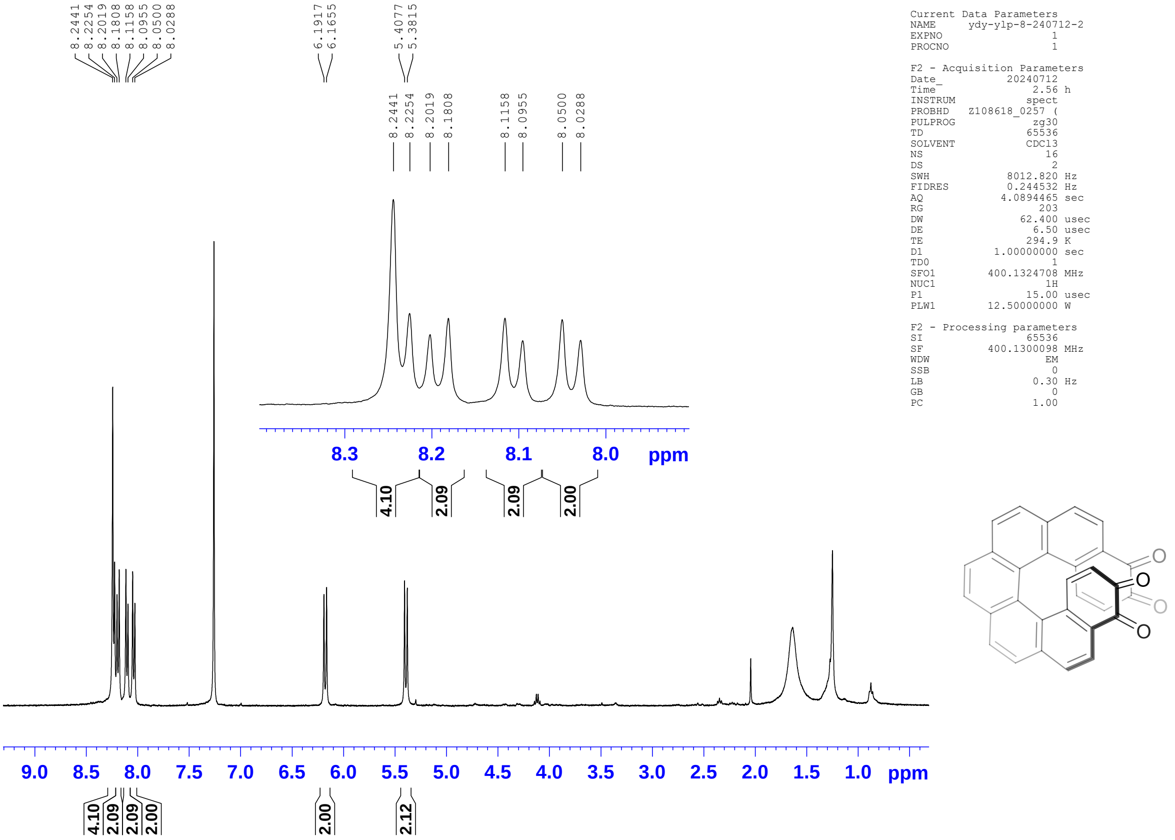

Figure S13. ^{1}H NMR spectrum of **9b** in $CDCl_3$ at 294K.

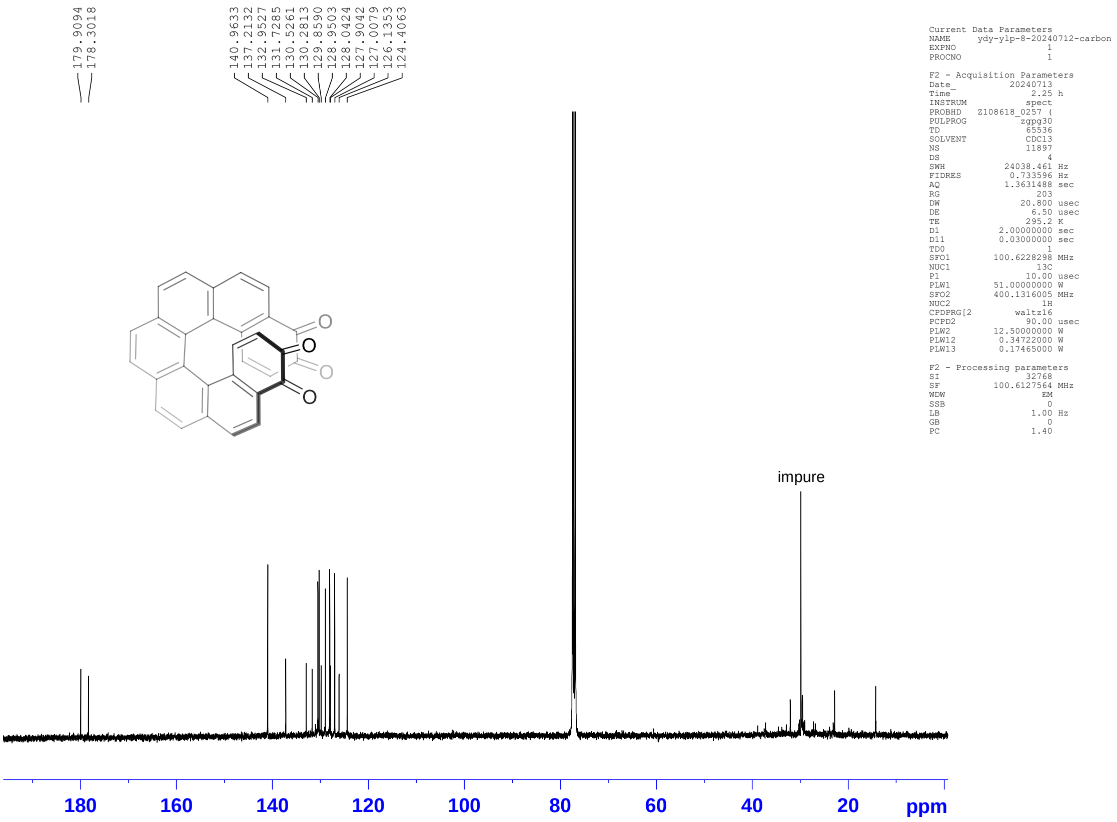

Figure S14. ^{13}C NMR spectrum of **9b** in $CDCl_3$ at 294K.

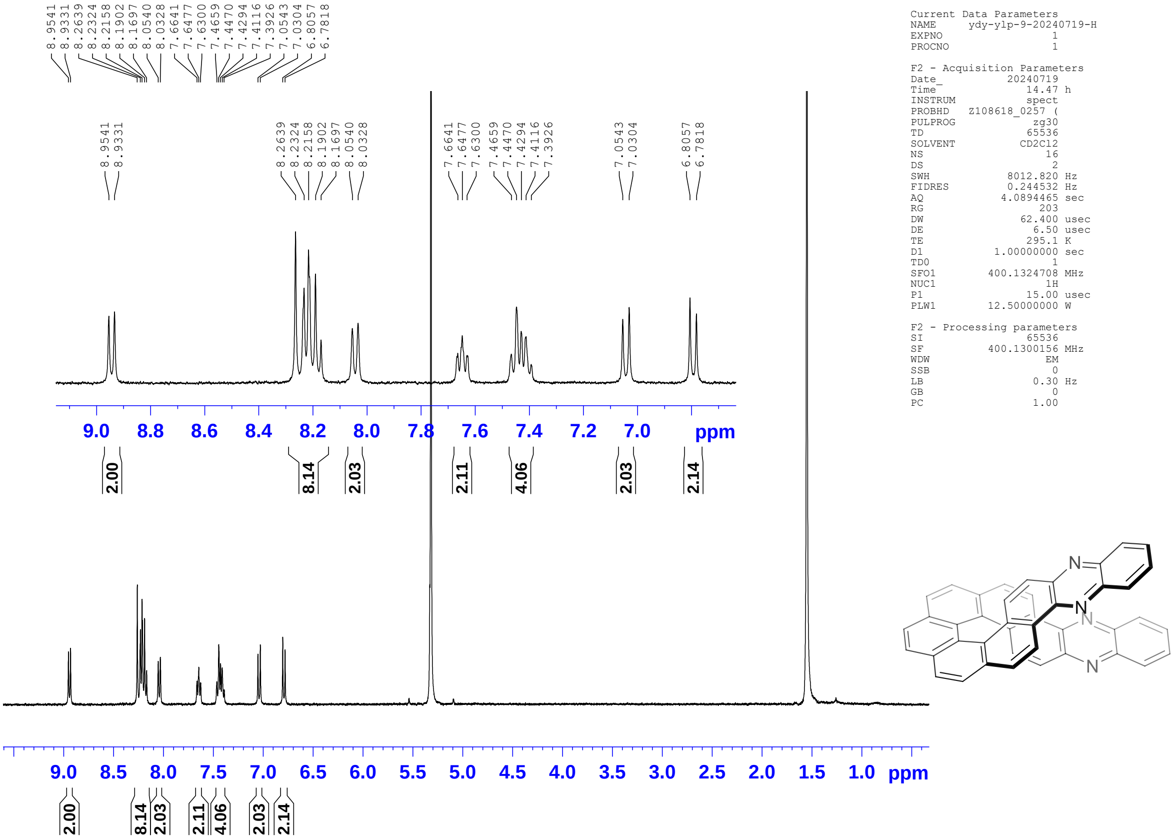

Figure S15. ^{1}H NMR spectrum of **3b** in CD_2Cl_2 at 294K.

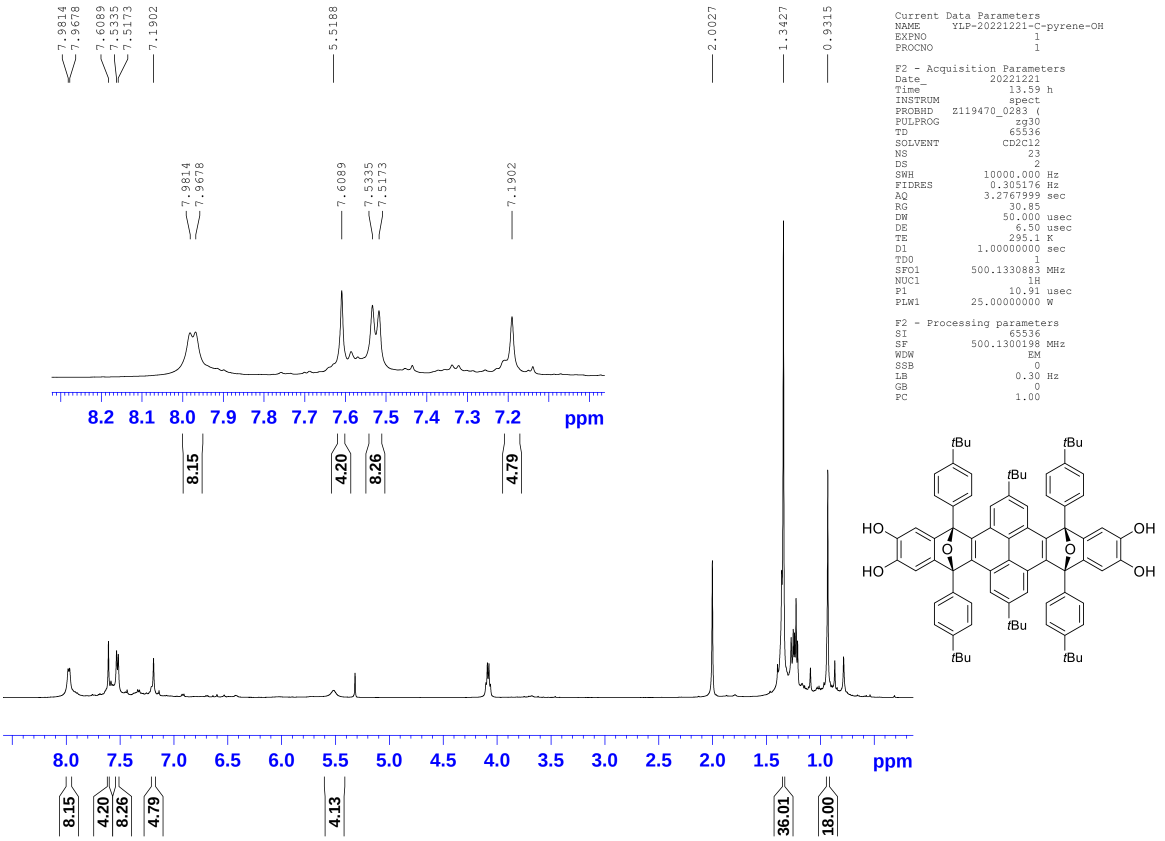

Figure S16. ^{1}H NMR spectrum of **4** in CD_2Cl_2 at 294K.

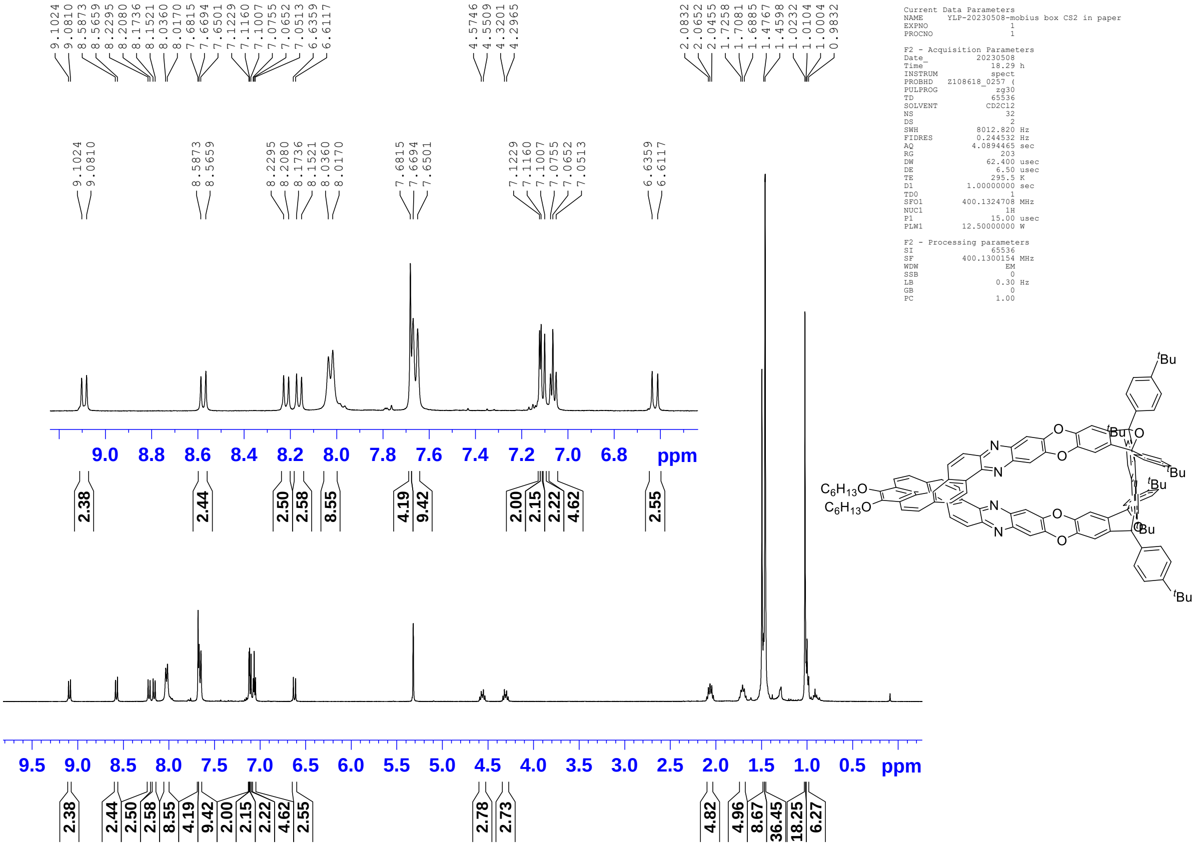

Figure S17. ^1H NMR spectrum of **2** in $CD_2Cl_2 + CS_2$ at 294K.

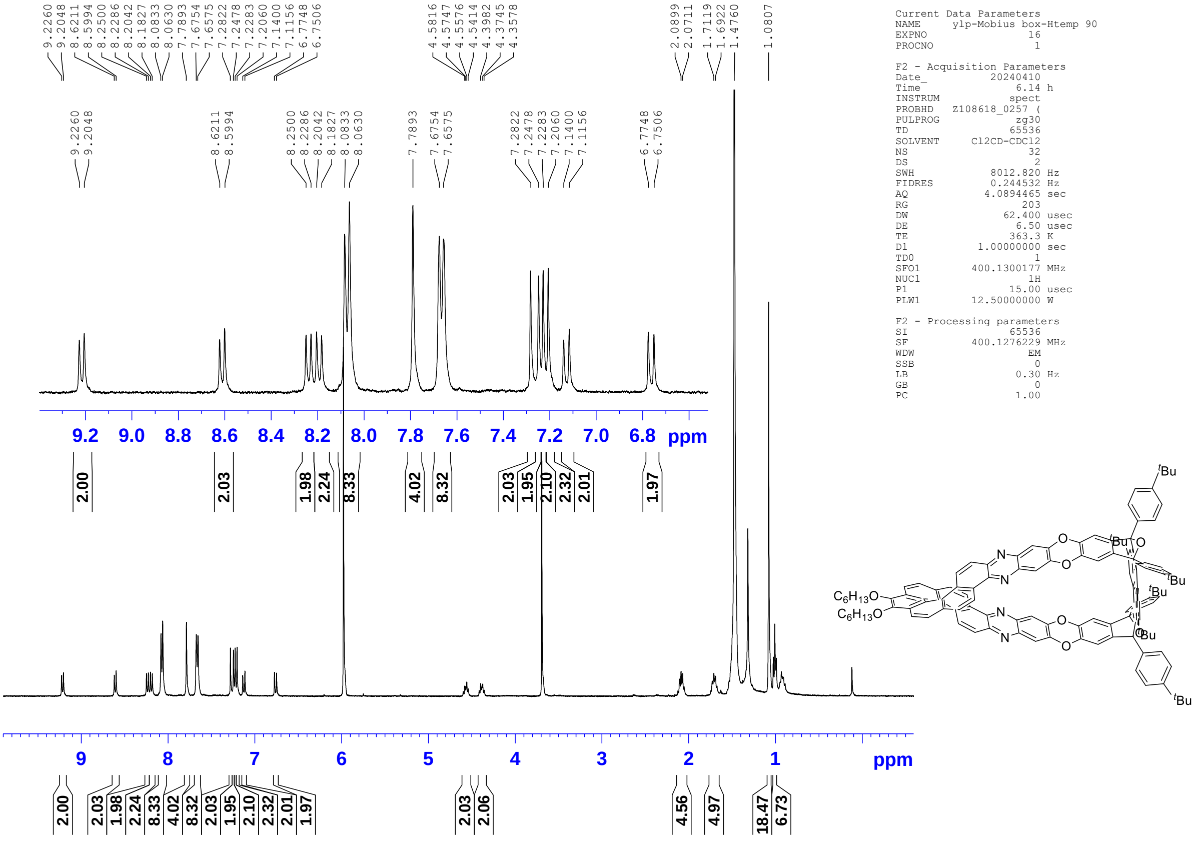

Figure S18. ^1H NMR spectrum of **2** in $C_2D_2Cl_4 + CS_2$ at 363K.

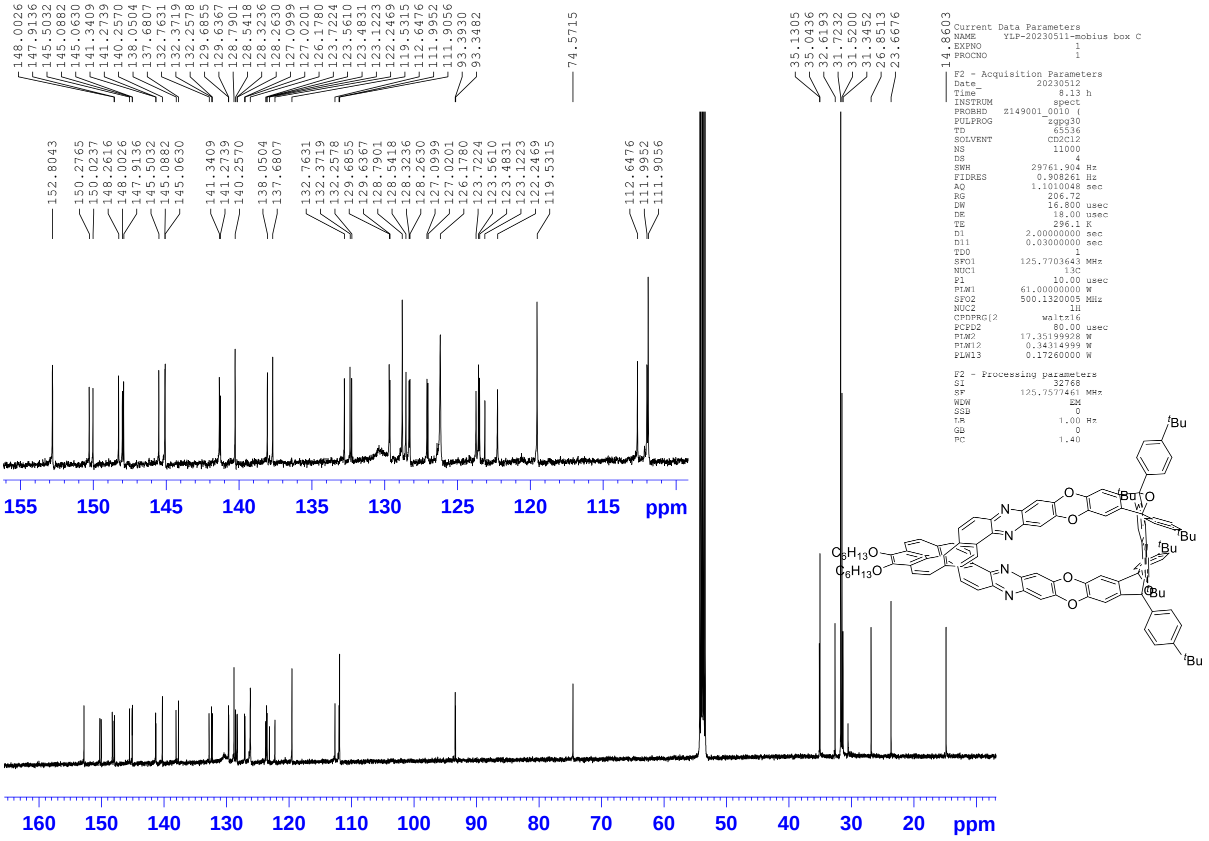

Figure S19. ^{13}C NMR spectrum of **2** in $CD_2Cl_2 + CS_2$ at 294K.

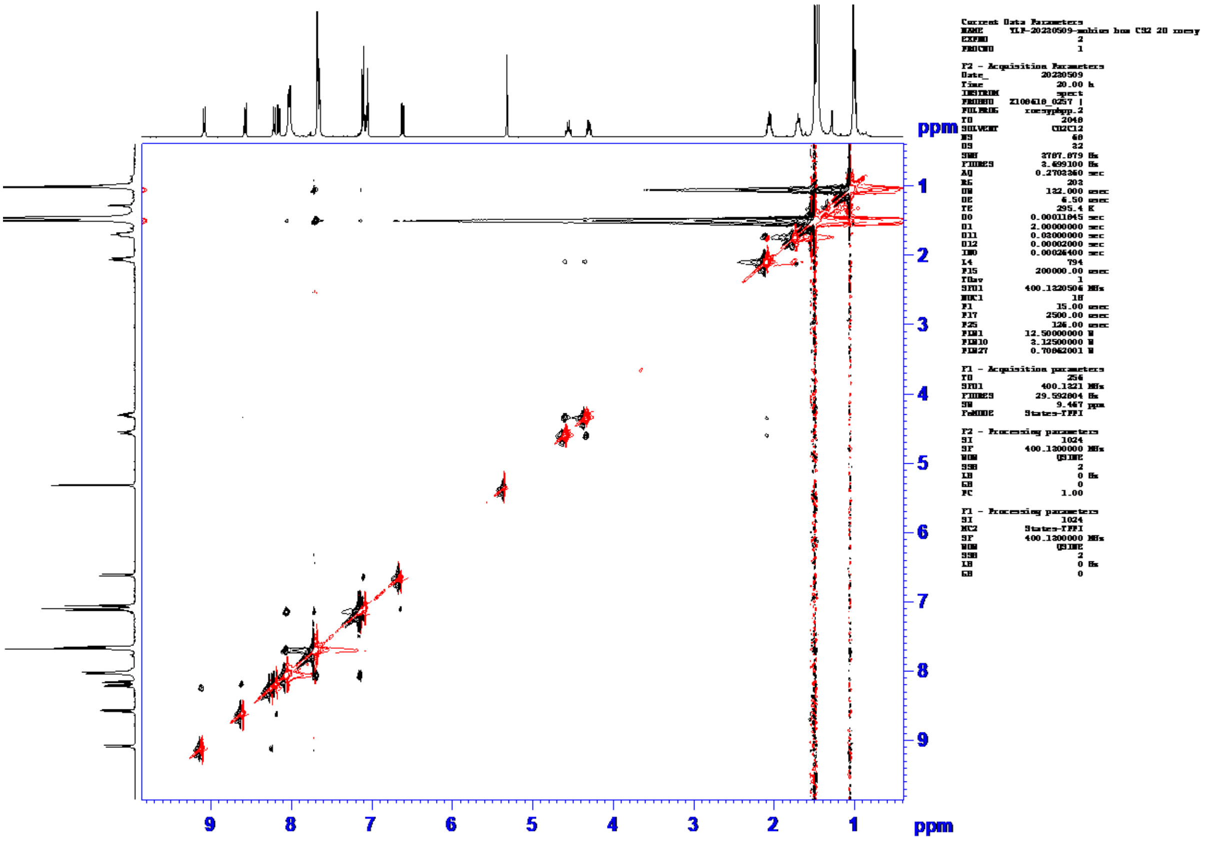

Figure S20. 2D ROESY NMR spectrum of **2** in $CD_2Cl_2 + CS_2$ at 294K.

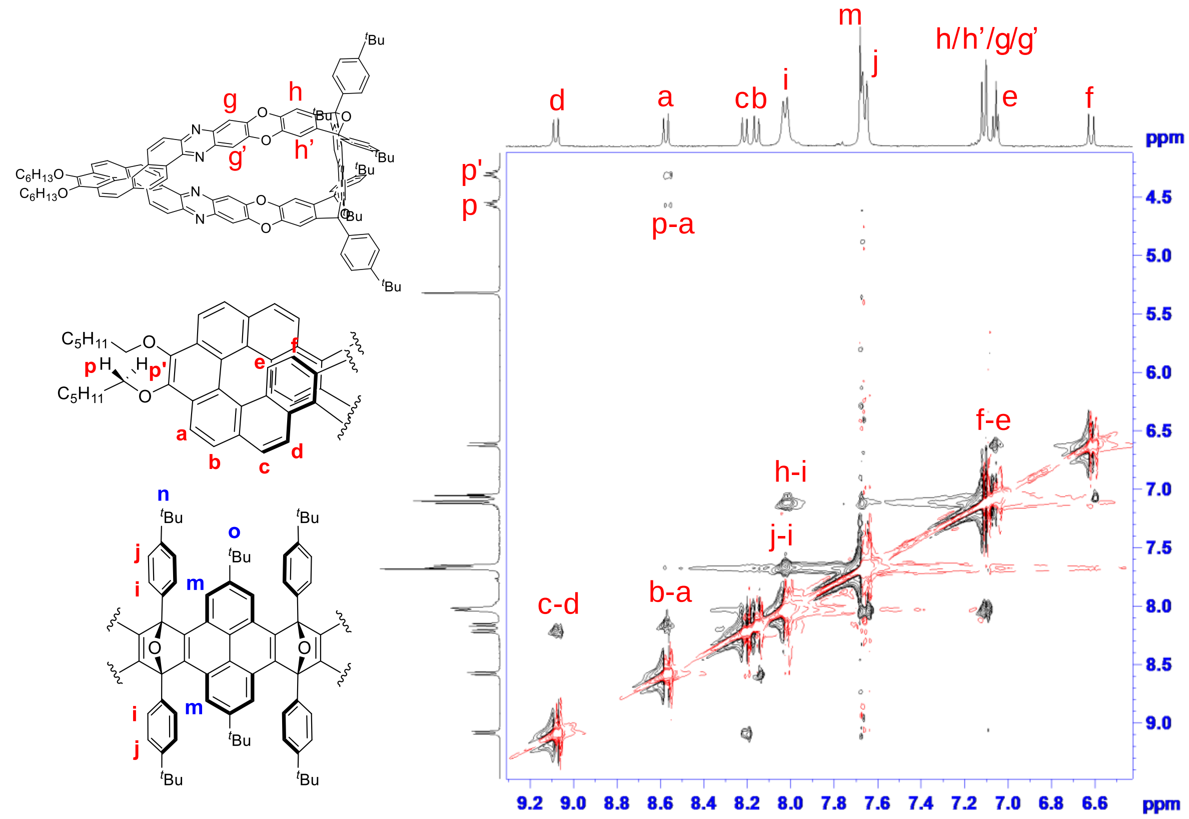

Figure S21. Partial 2D ROESY NMR spectrum of **2** in CD_2Cl_2 + CS_2 at 294K. (Protons *a-f* on helicene part are assigned starting from coupling between proton *p/p'* and proton *a*.)

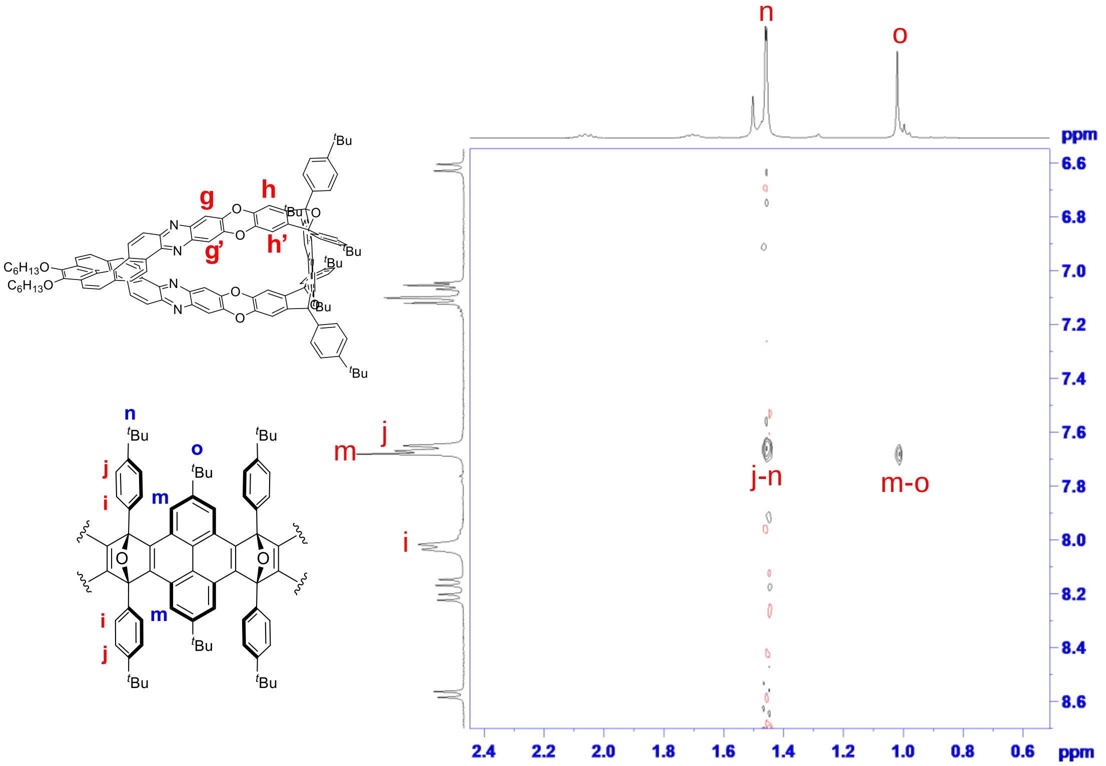

Figure S22. Partial 2D ROESY NMR spectrum of **2** in CD_2Cl_2 + CS_2 at 294K. (Protons *i, j, m* are assigned starting from coupling between proton *j* and proton *n*.)

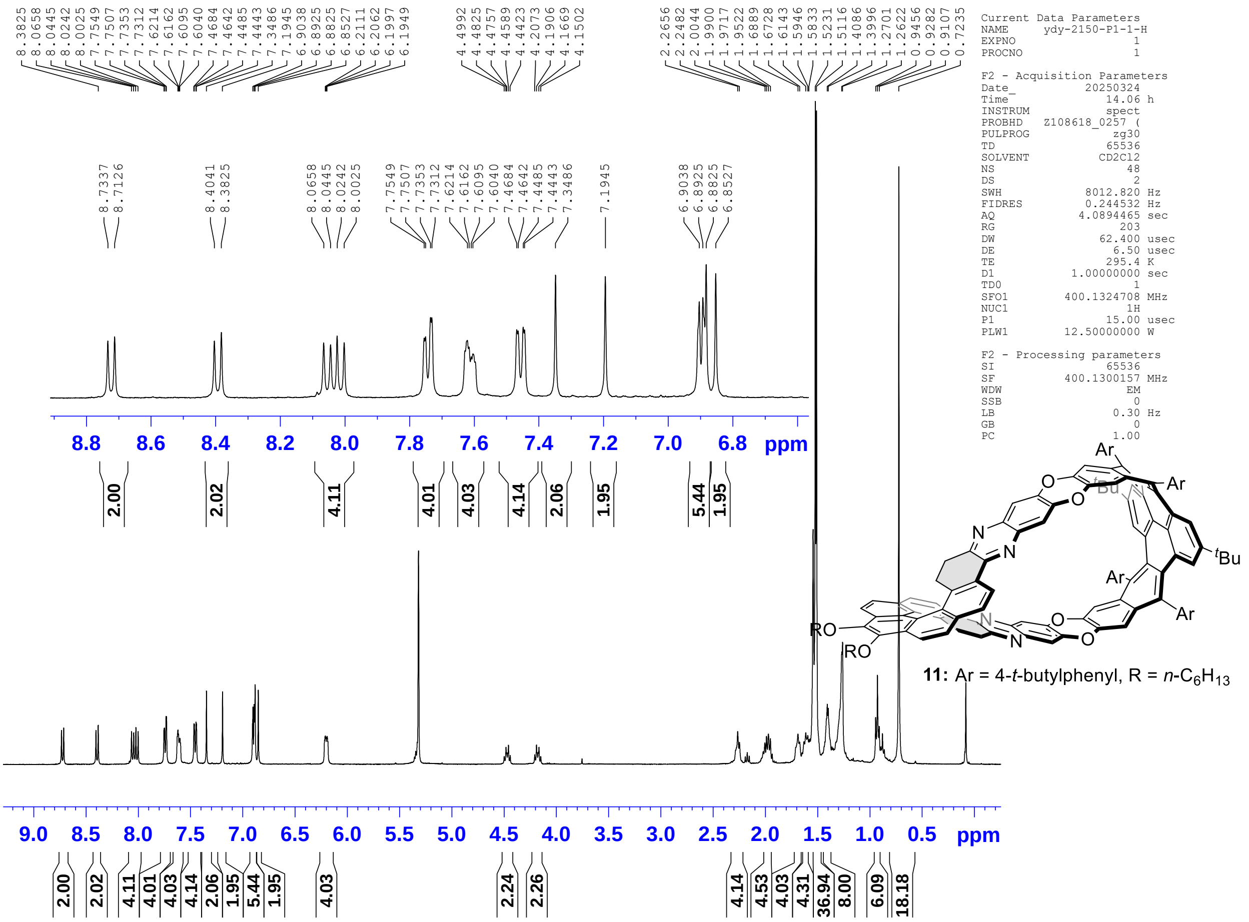

Figure S23. ^{1}H NMR spectrum of **11** in CD_2Cl_2 at 294K.

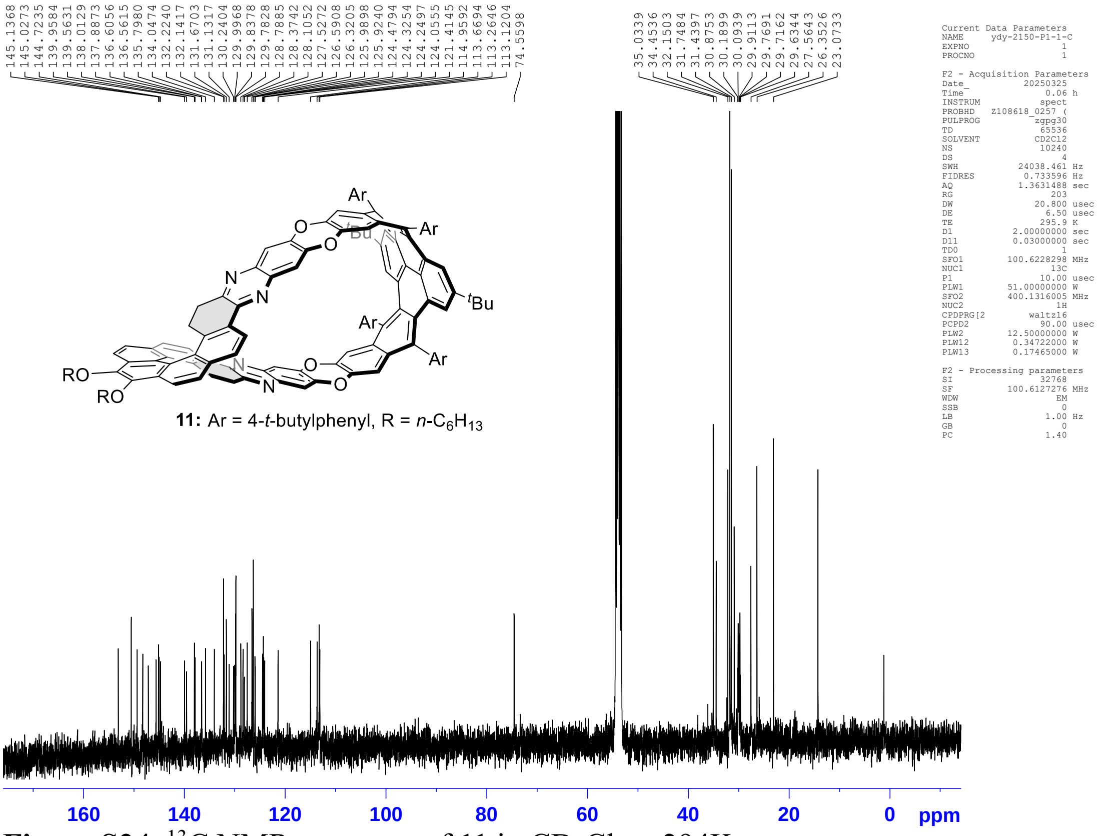

Figure S24. ^{13}C NMR spectrum of **11** in CD_2Cl_2 at 294K.

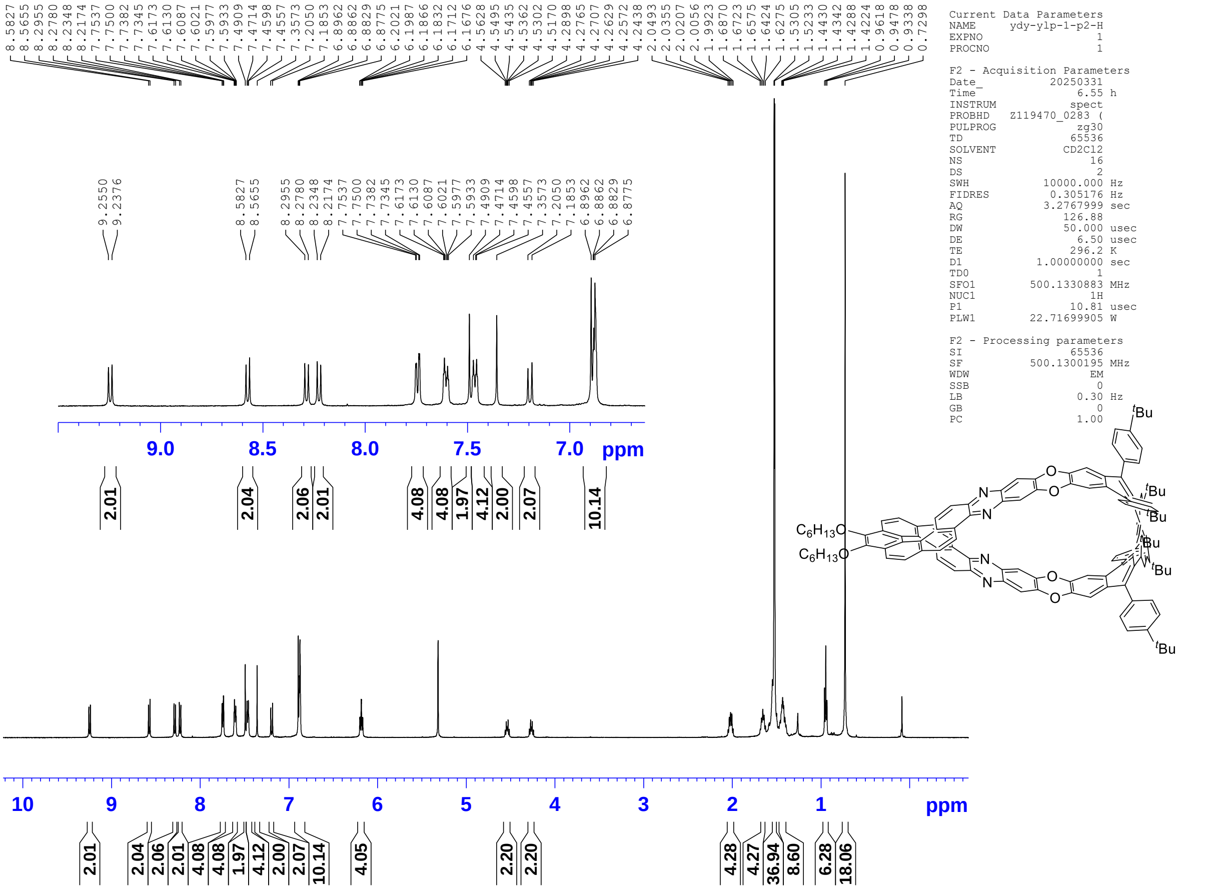

Figure S25. ^{1}H NMR spectrum of **1** in CD_2Cl_2 at 294K.

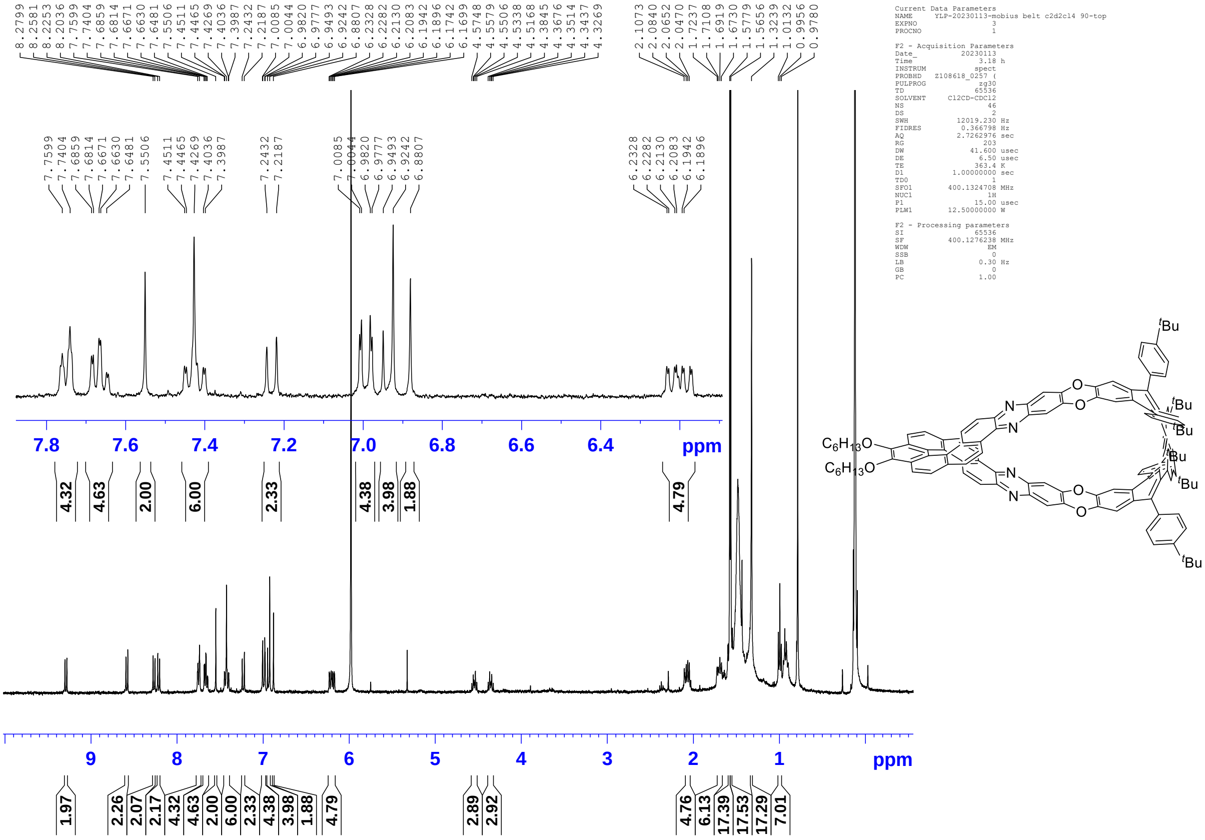

Figure S26. ^{1}H NMR spectrum of **1** in $C_2D_2Cl_4 + CS_2$ at 363K.

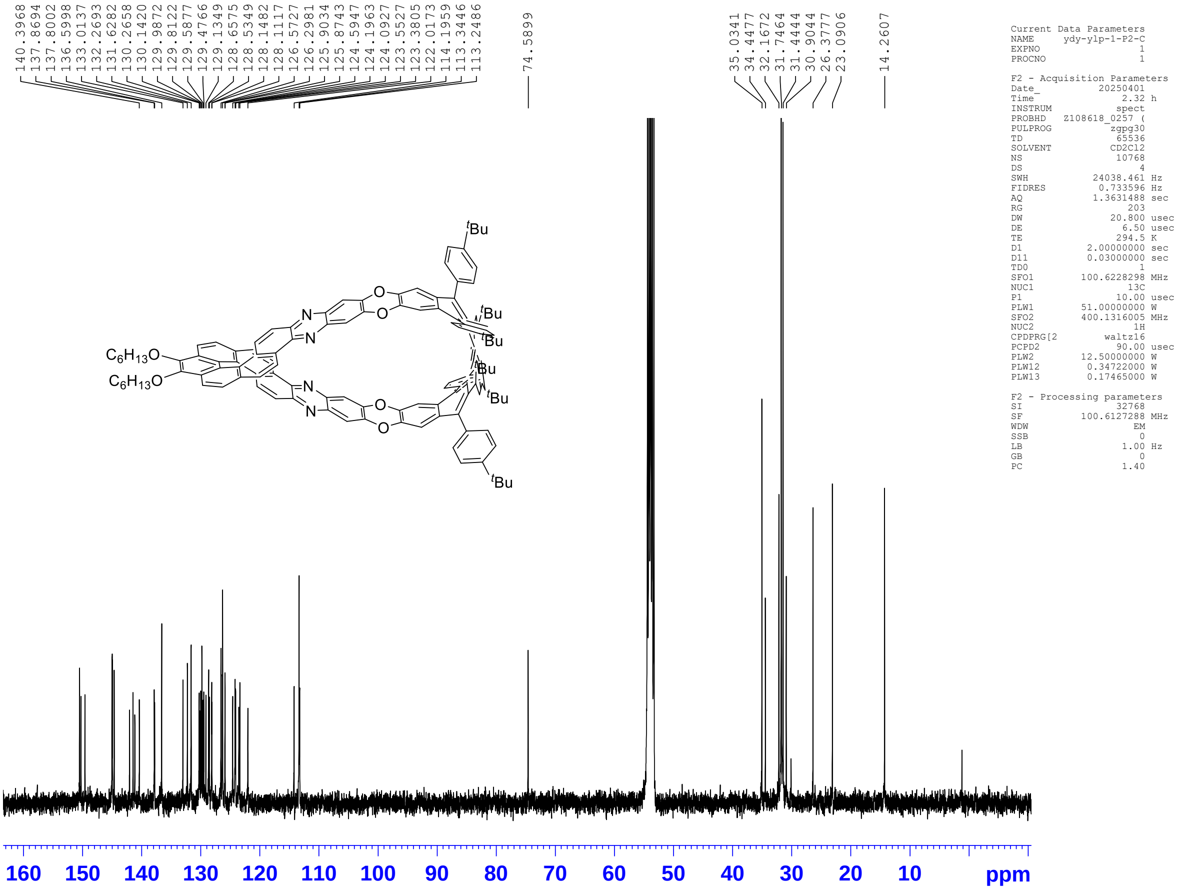

Figure S27. ^{13}C NMR spectrum of **1** in CD_2Cl_2 at 294K.

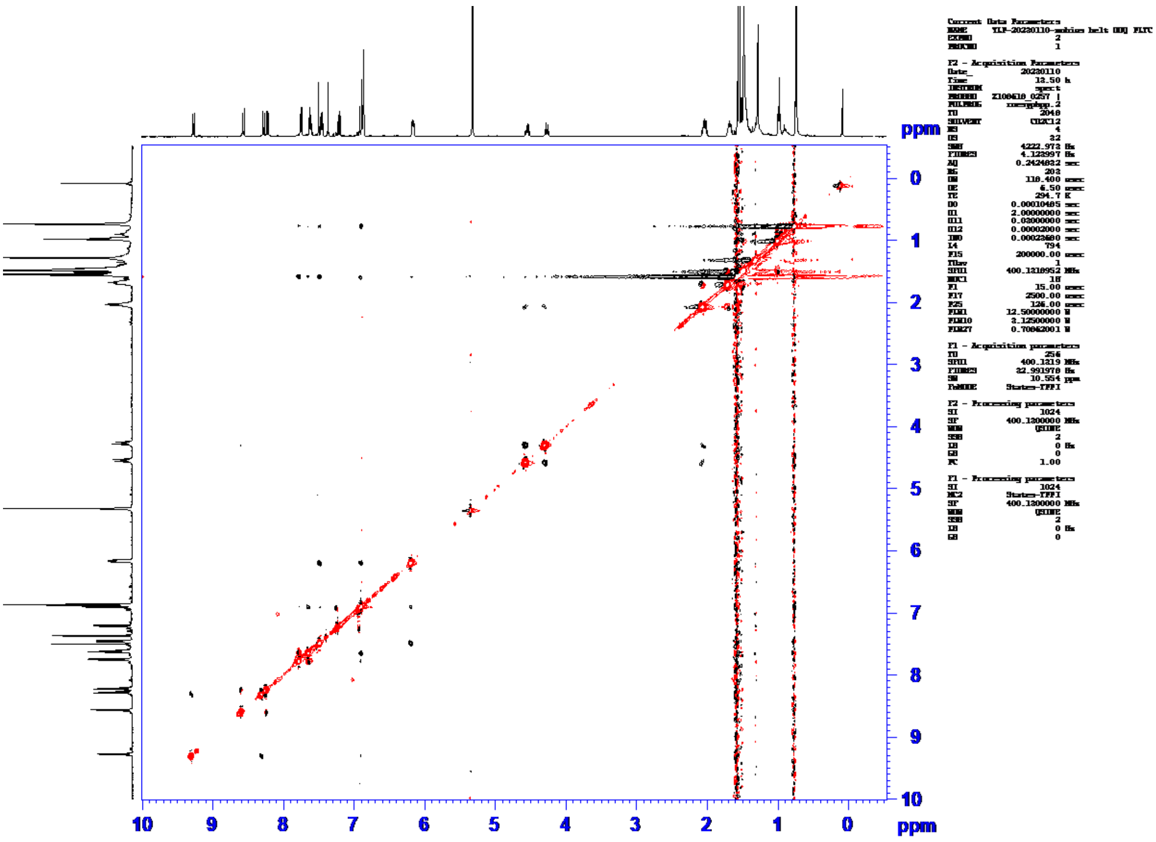

Figure S28. 2D ROESY NMR spectrum of **1** in $CD_2Cl_2 + CS_2$ at 294K.

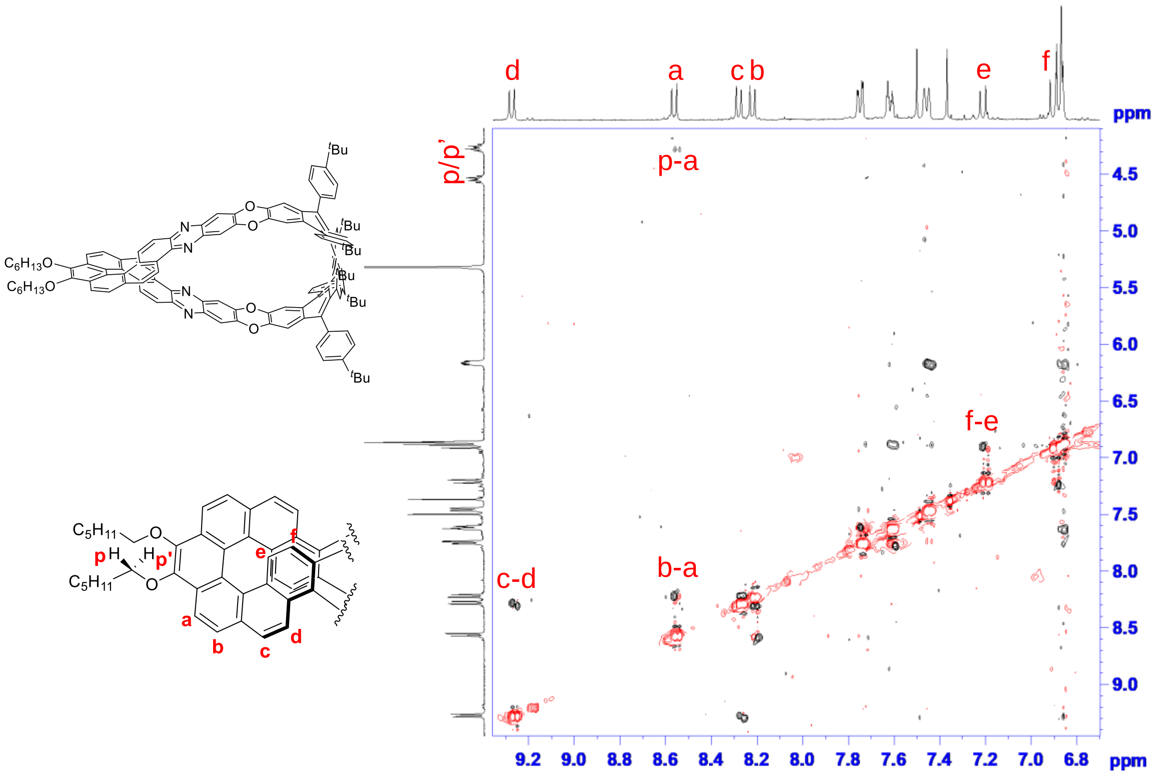

Figure S29. Partial 2D ROESY NMR spectrum of **1** in CD_2Cl_2 + CS_2 at 294K. (Protons *a-f* on helicene part are assigned starting from coupling between proton *p/p'* and proton *a*.)

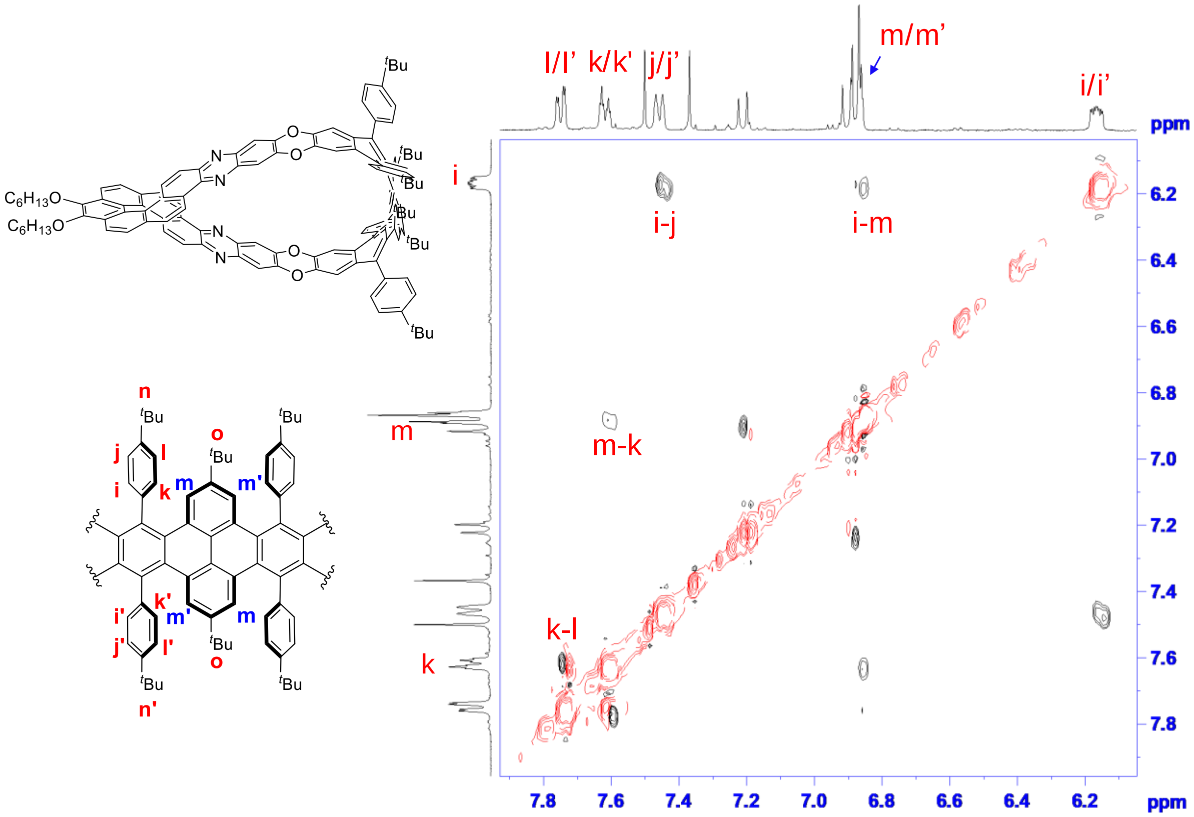

Figure S30. Partial 2D ROESY NMR spectrum of **1** in CD_2Cl_2 + CS_2 at 294K.

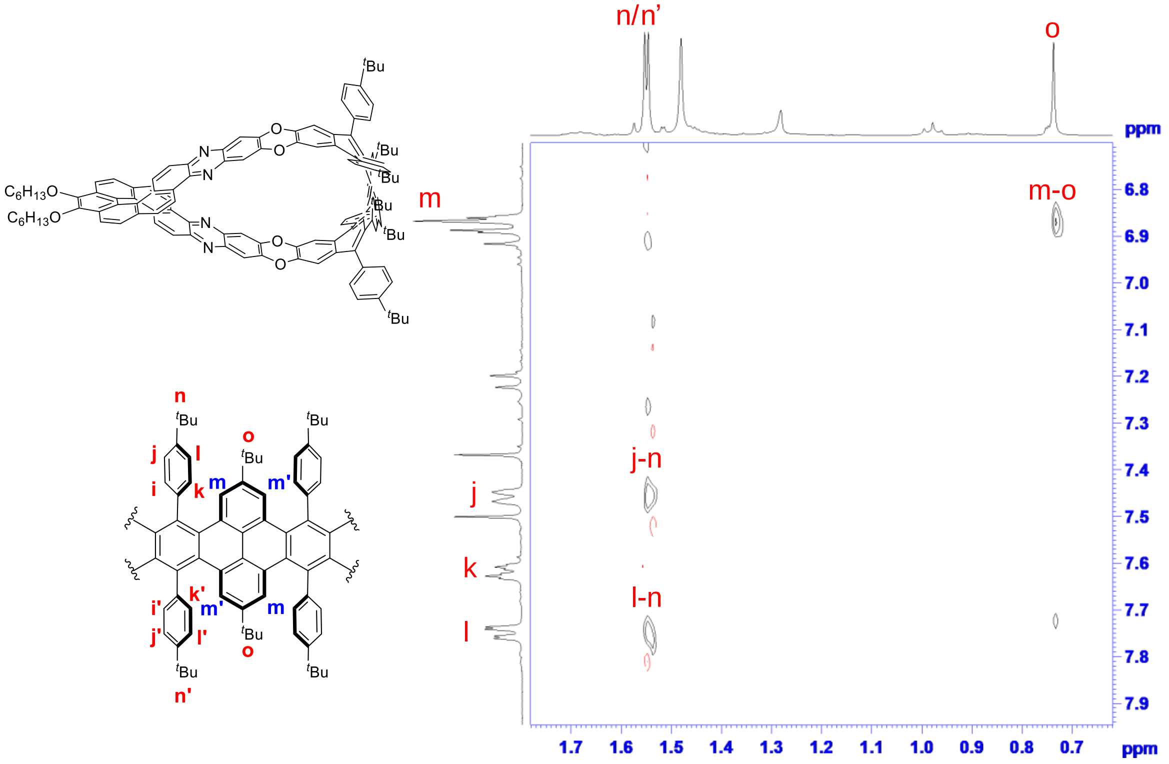

Figure S31. Partial 2D ROESY NMR spectrum of **1** in CD_2Cl_2 + CS_2 at 294K. (Protons *i, j, m* are assigned starting from coupling between proton *j* and proton *n*.)

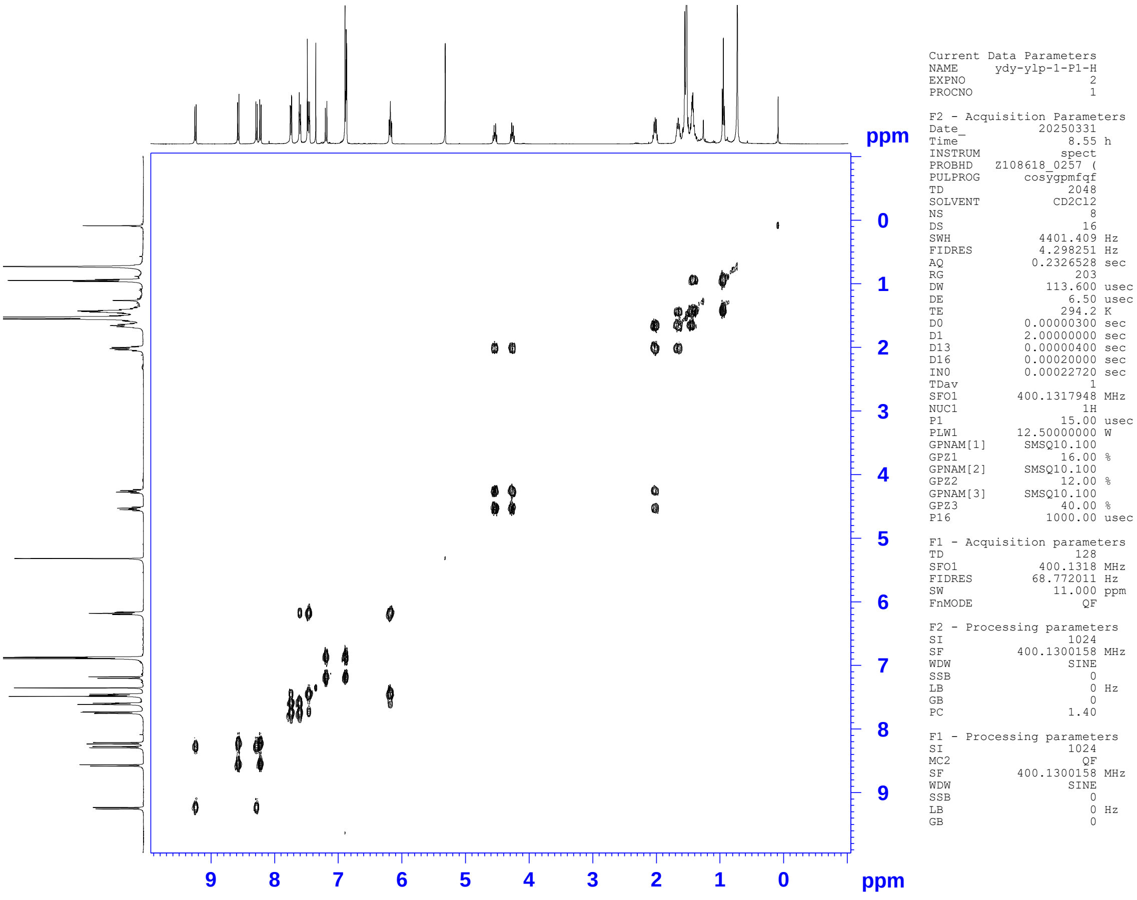

Figure S32. 2D COSY NMR spectrum of **1** in CD_2Cl_2 at 294K.

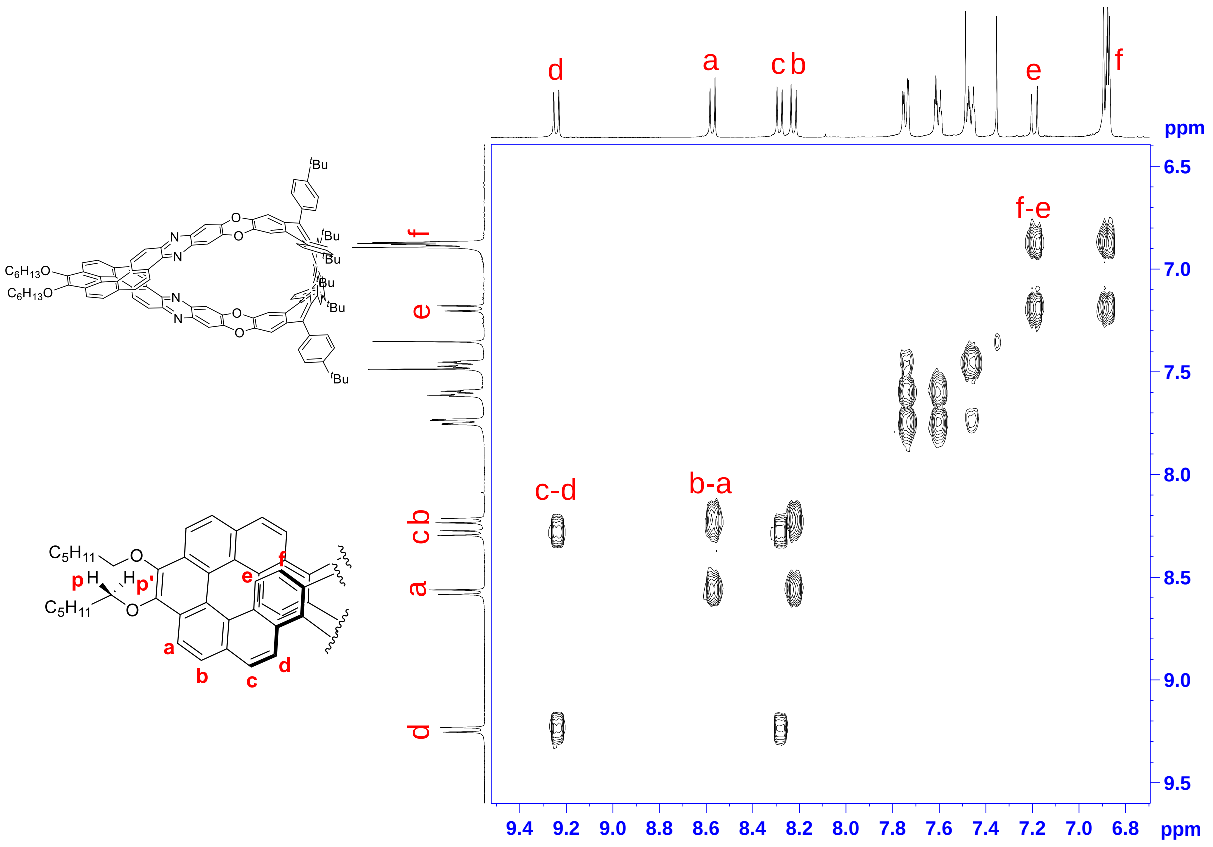

Figure S33. Partial 2D COSY NMR spectrum of **1** in CD_2Cl_2 at 294K.

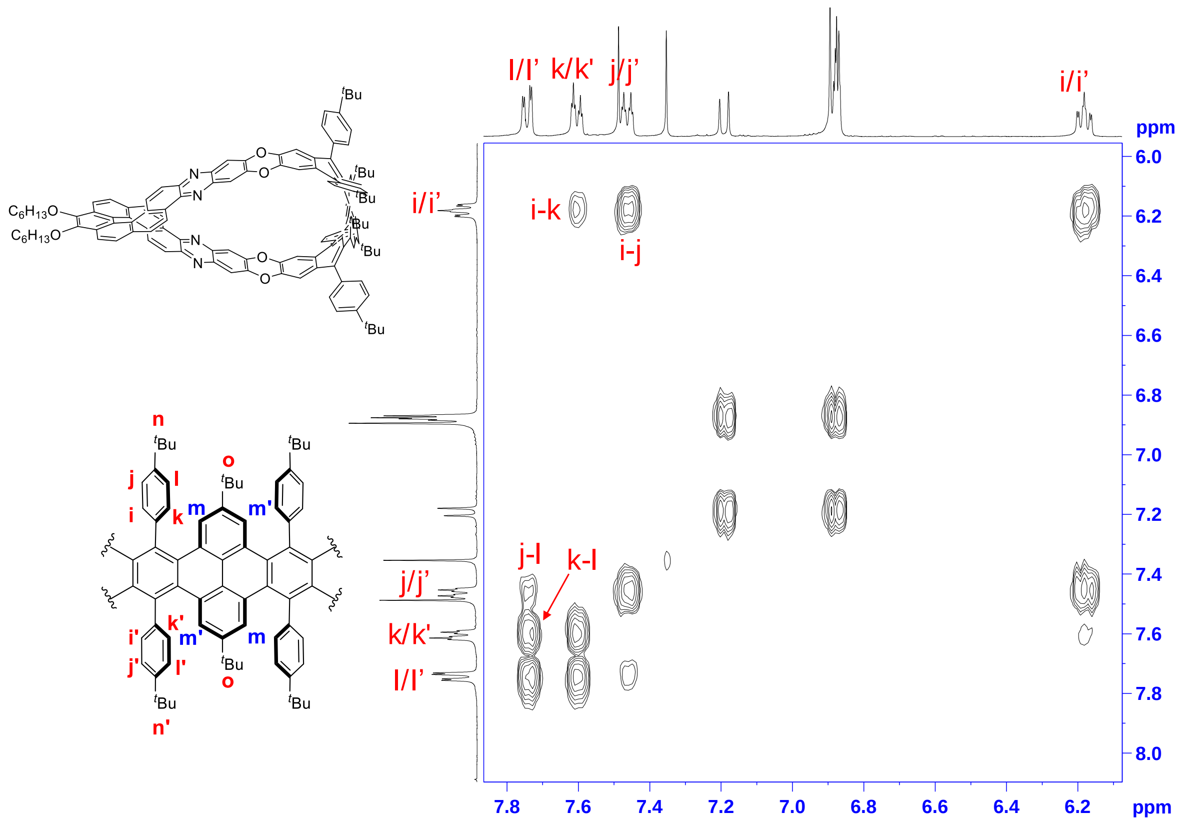

Figure S34. Partial 2D COSY NMR spectrum of **1** in CD_2Cl_2 at 294K.

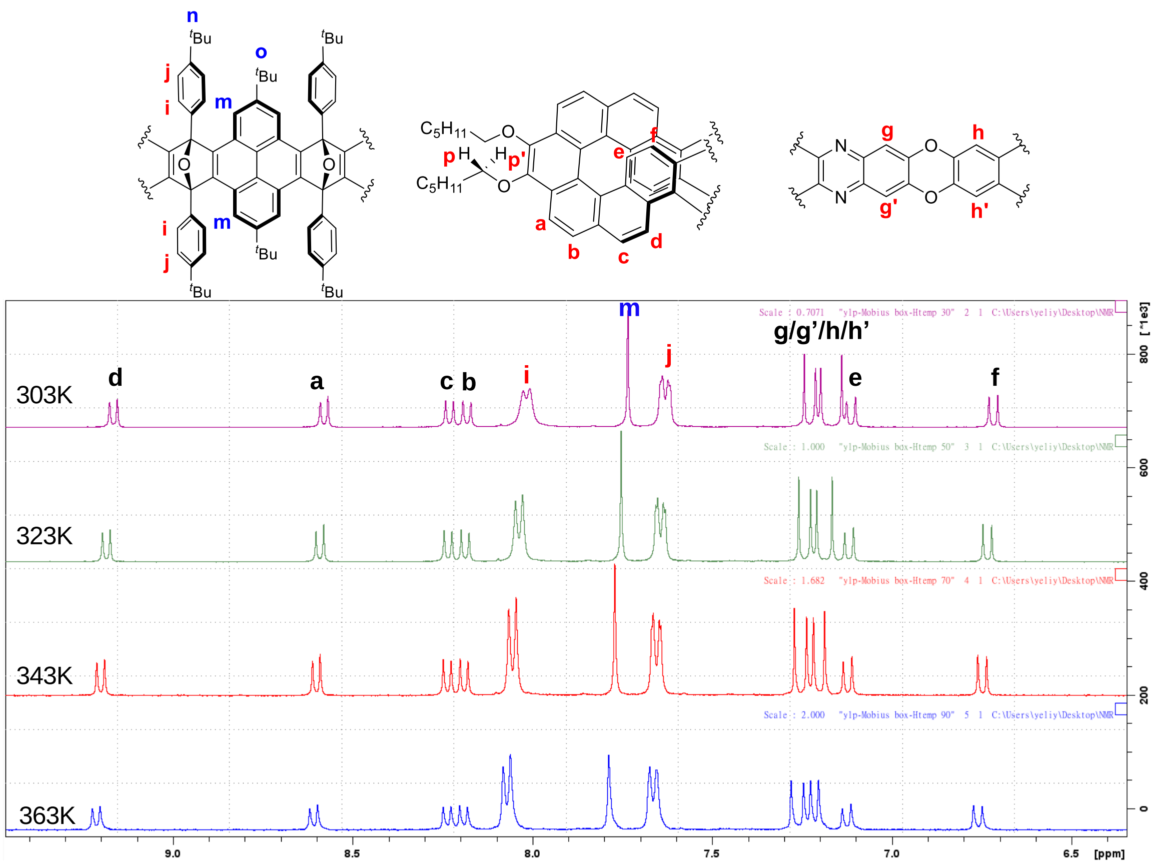

Figure S35. Partial VT ^{1}H NMR spectra of **2** in $C_2D_2Cl_4 + CS_2$.

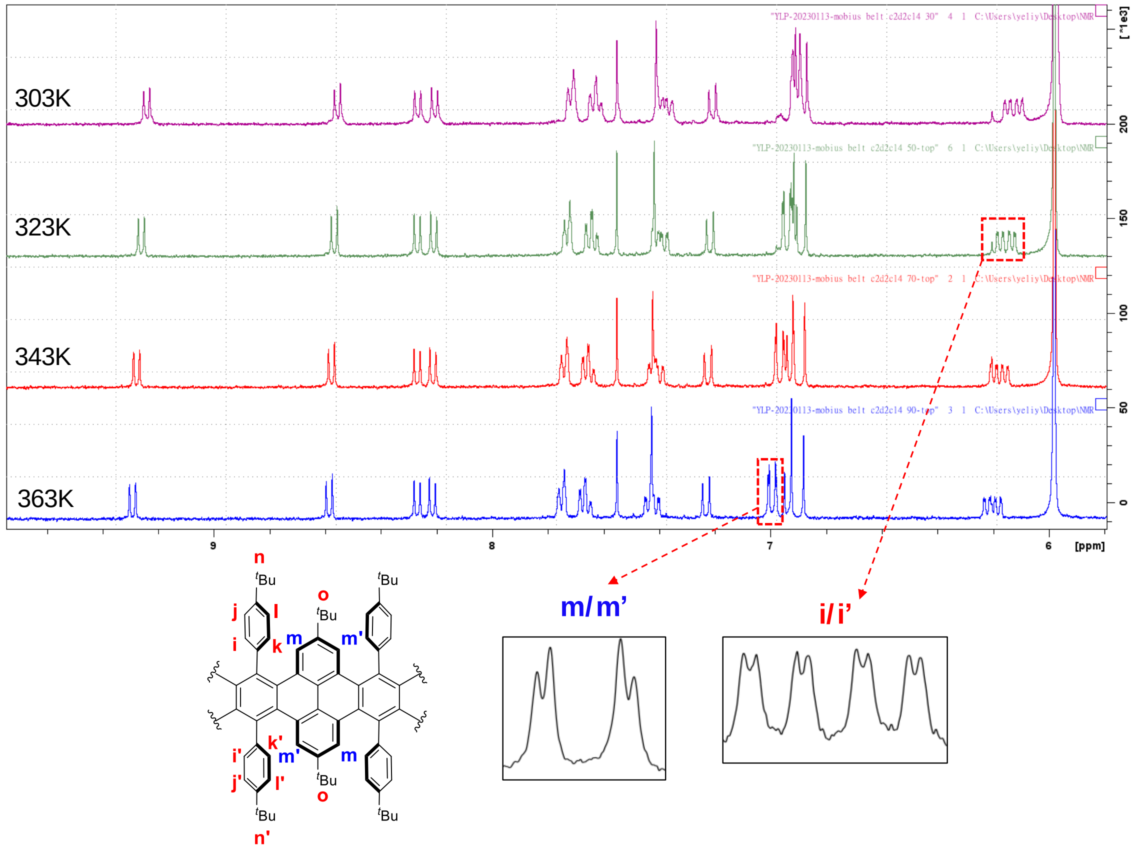

Figure S36. Partial VT ^{1}H NMR spectra of **1** in $C_2D_2Cl_4$.

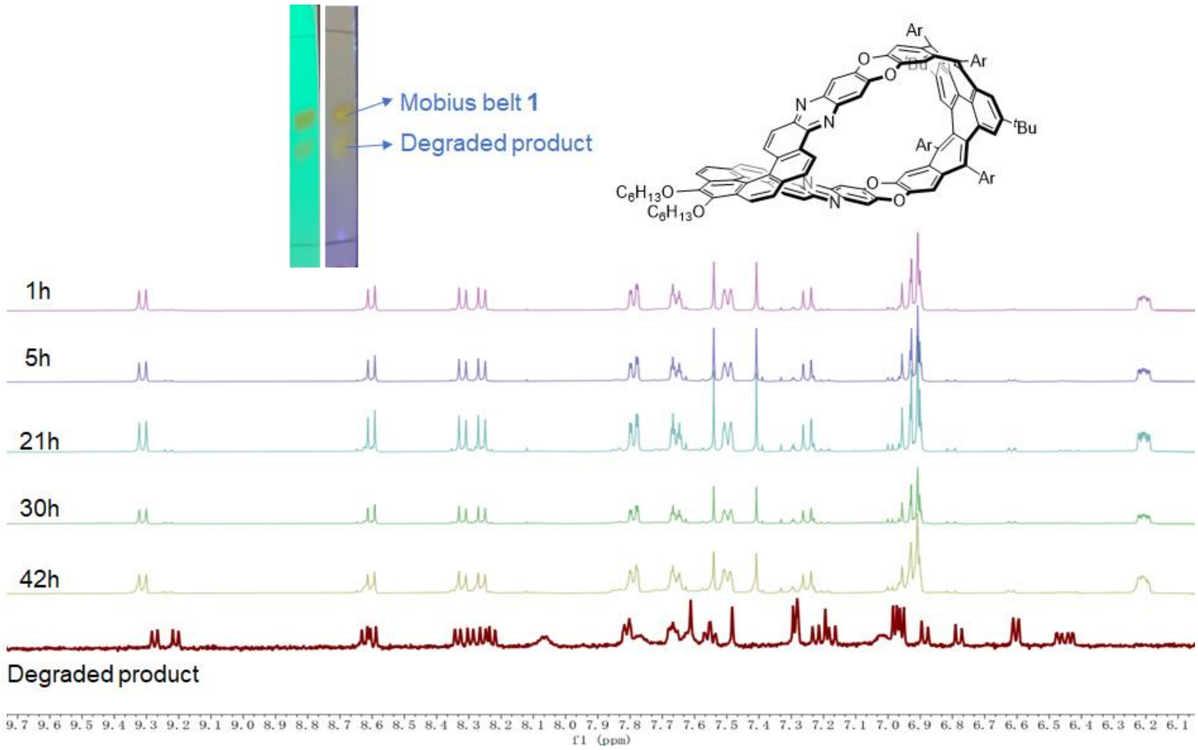

Figure S37. Partial ^{1}H NMR spectra of **1** recorded from its solution in CD_2Cl_2 exposed to air under ambient light at room temperature for indicated periods of time, compared to that of the degraded product. (The degraded product, referred to the spot below compound **1** on TLC, was separated by PTLC from the solution of 1 exposed to air and ambient light for 42 h.)

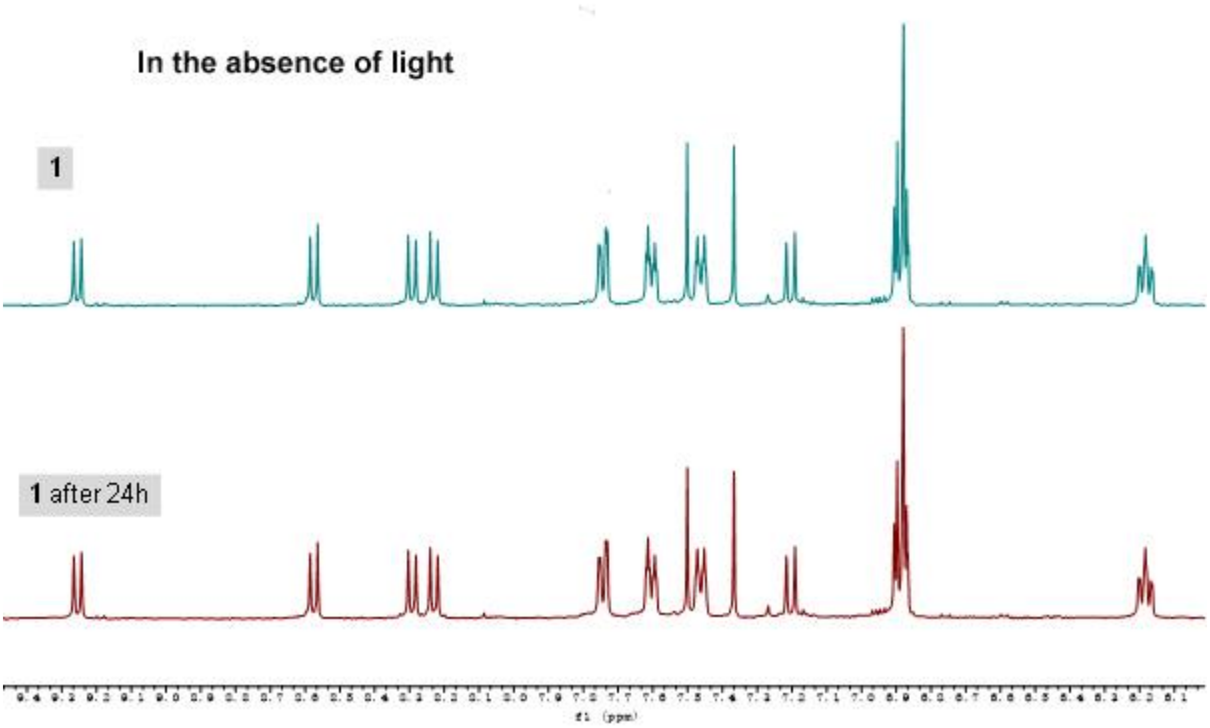

Figure S38. Partial ^{1}H NMR spectra (400MHz) of **1** before and after its solution in CD_2Cl_2 was exposed to air in the absence of light for 24 hours.

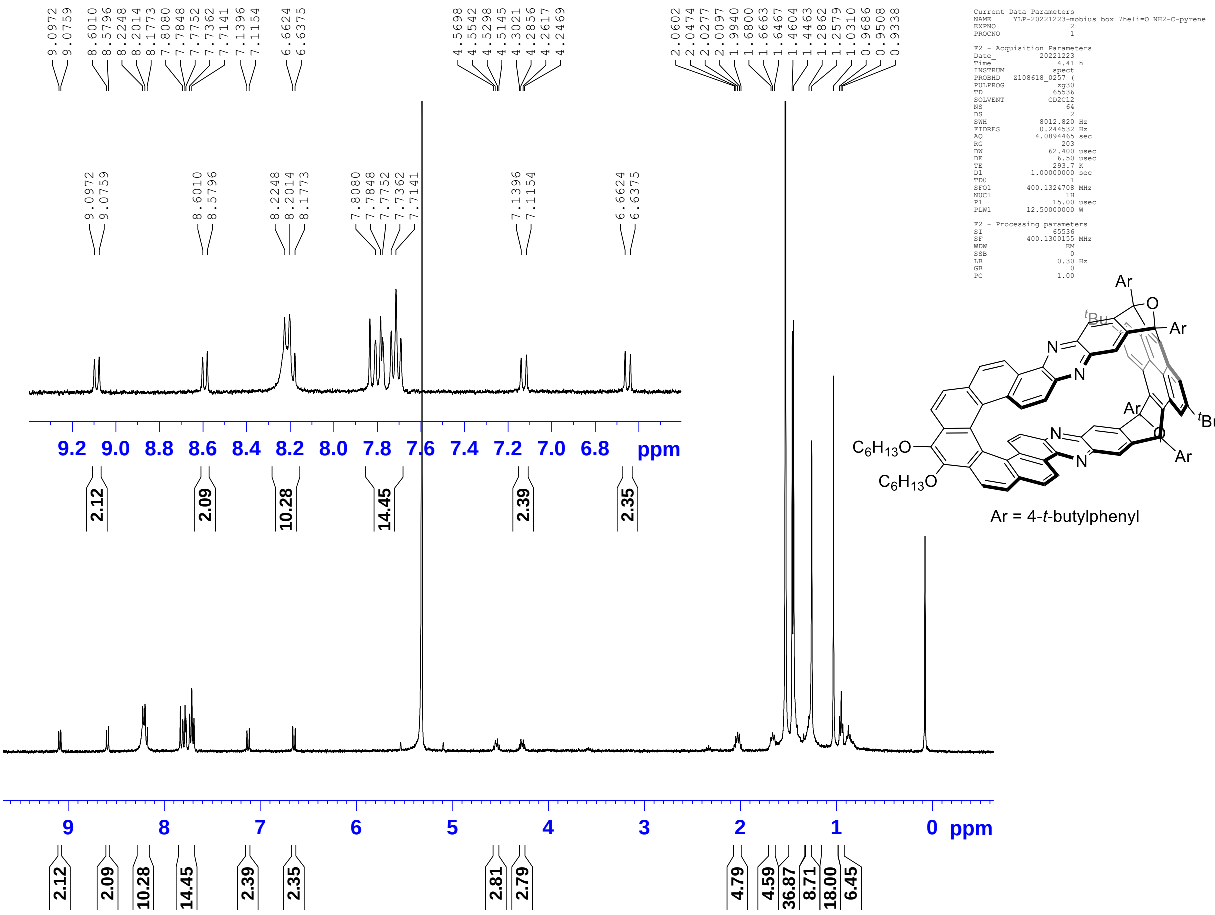

Figure S39. ^{1}H NMR spectrum of **14** in CD_2Cl_2 at 294K.

3. High-resolution mass spectra

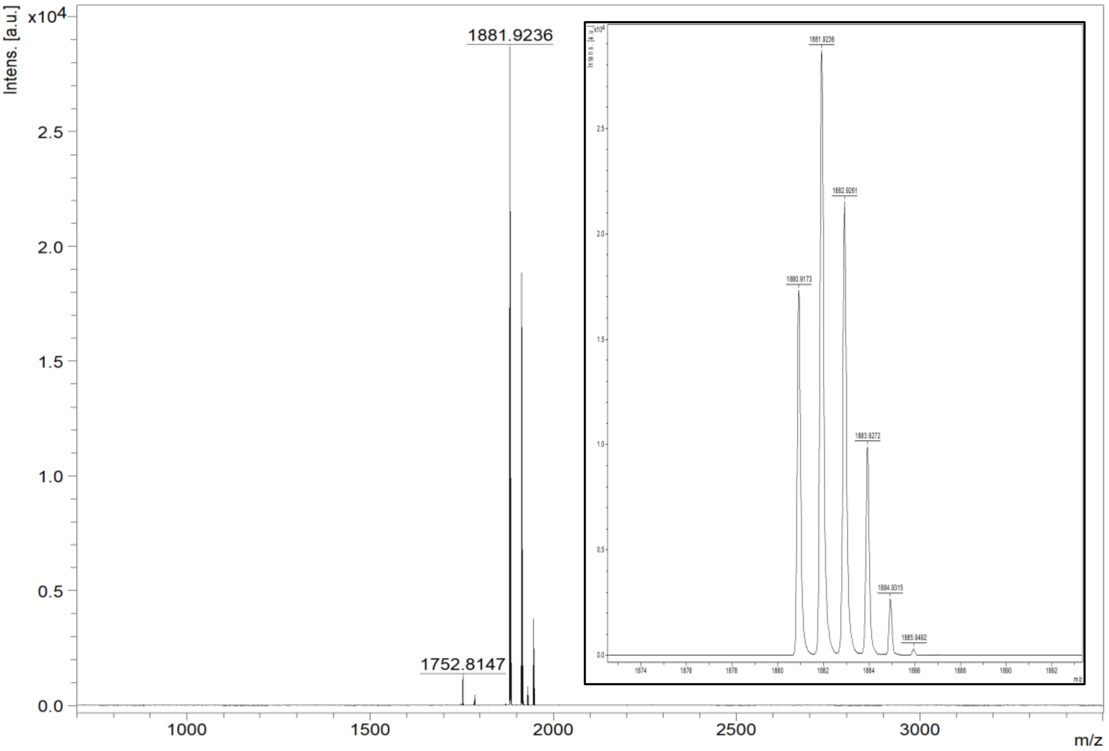

Figure S40. High resolution mass spectra (MALDI-TOF) of **1** ($C_{134}H_{120}N_4O_6$).

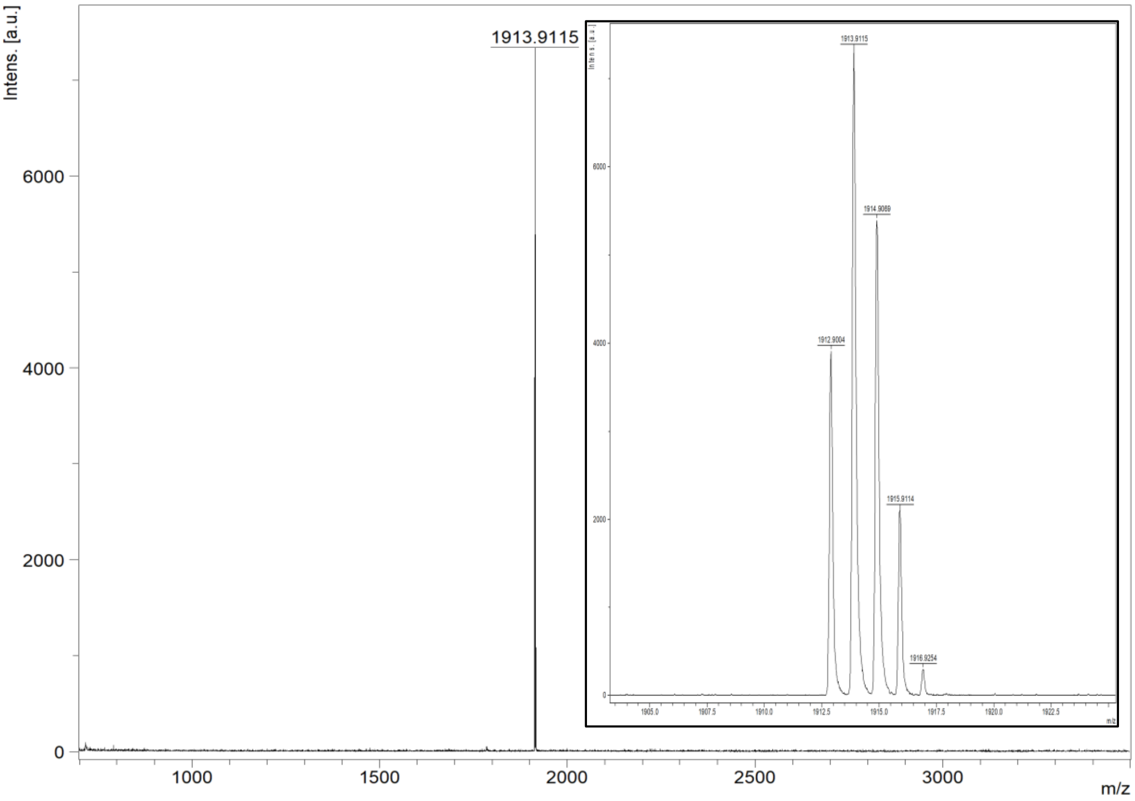

Figure S41. High resolution mass spectra (MALDI-TOF) of **2** ($C_{134}H_{120}N_4O_8$).

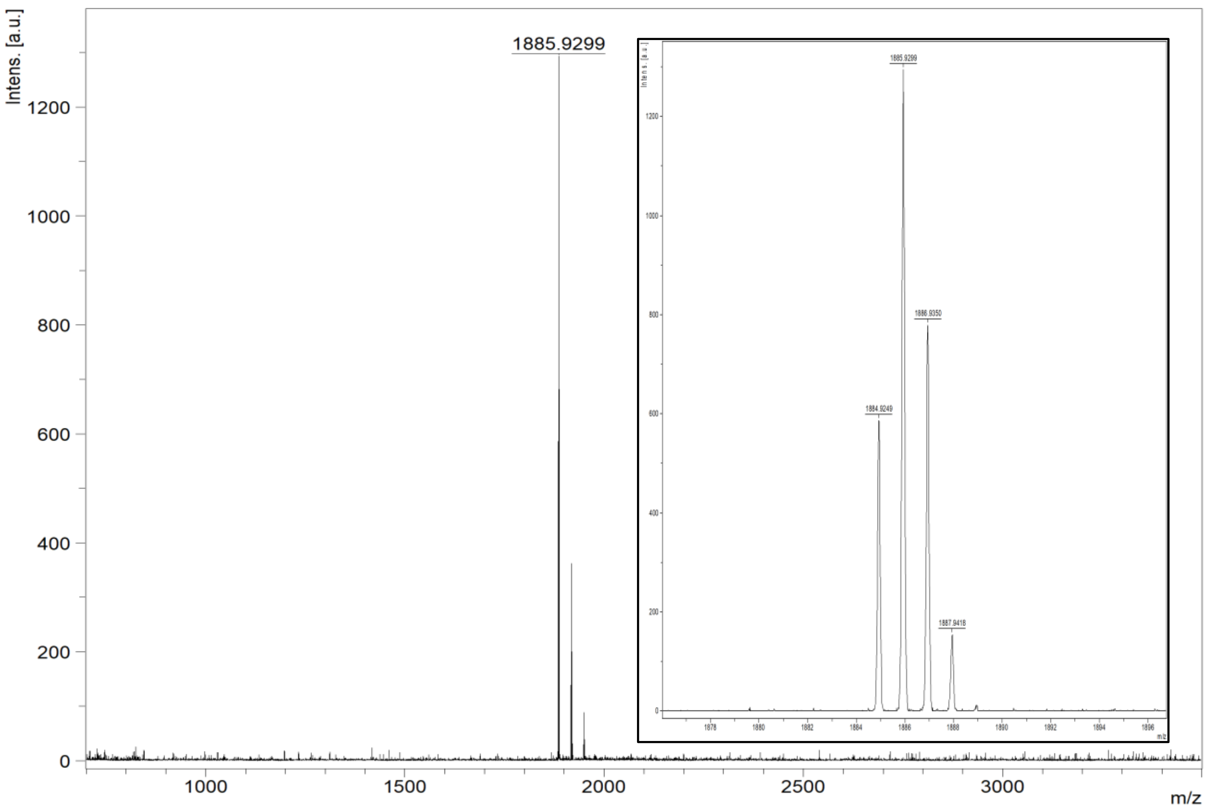

Figure S42. High resolution mass spectra (MALDI-TOF) of **11** ($C_{134}H_{124}N_4O_6$).

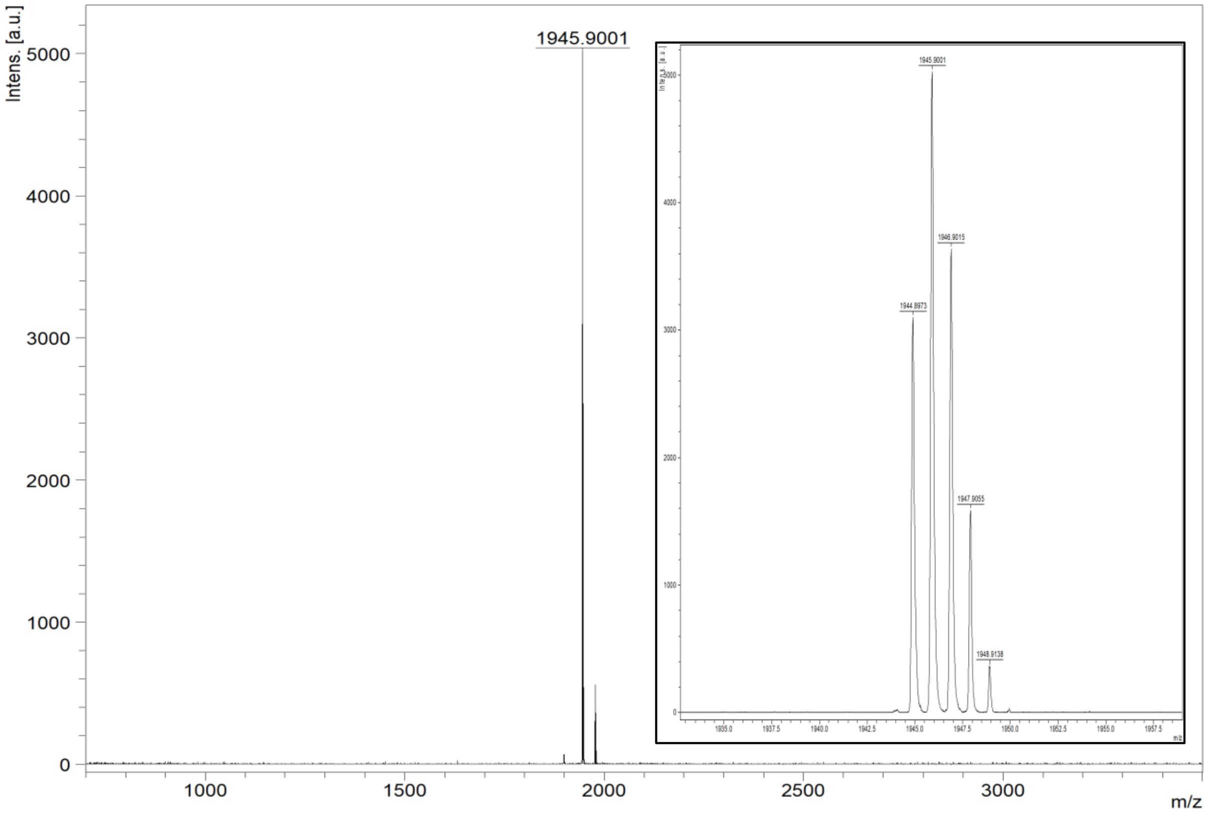

Figure S43. High resolution mass spectra (MALDI-TOF) for the degradation product of **1**, which has a molecular formula of $C_{134}H_{120}N_4O_{10}$.

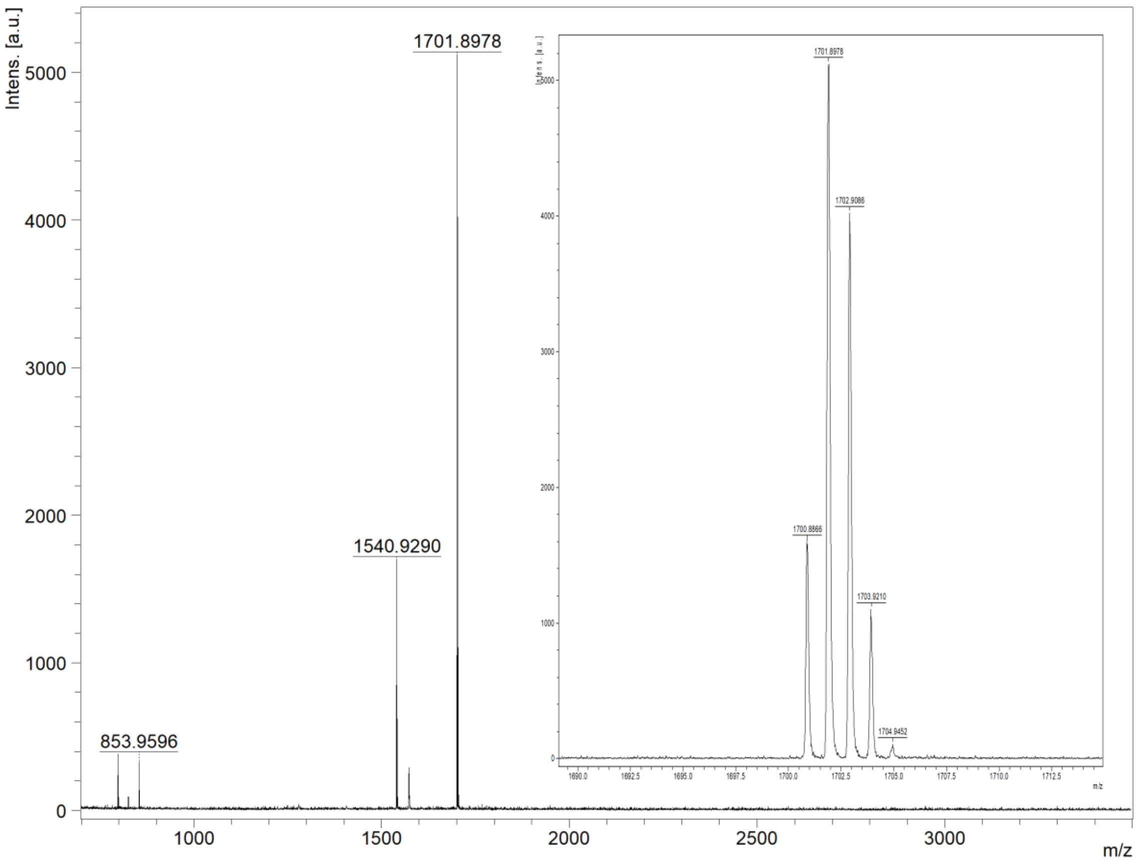

Figure S44. High resolution mass spectra (MALDI-TOF) for compound **14**, which has a molecular formula of $C_{122}H_{116}N_4O_4$.

4. Photophysical properties

UV-vis absorption spectra were recorded on a Varian CARY 1E UV-vis spectrophotometer. Fluorescence spectra were taken on a Hitachi F-4500 spectrofluorometer. Optical rotation was performed by Rudolph Autopol II polarimeter. Circular dichroism (CD) spectra were recorded on an Applied Photophysics Chirascan TM Spectrometer. Circularly polarized luminescence (CPL) spectra were recorded on an Applied photophysics Chirascan V100 circular dichroism spectrometer. All optical measurements were performed at room temperature under ambient condition. The fluorescence lifetime and absolute fluorescence quantum yield were determined by standard procedure using a FLS920P fluorescence spectrometer (Edinburgh Instrument) equipped with an integrating sphere with its inner face coated with BENFLEC® (Edinburgh Instrument). Spectral correction curves were provided by Edinburgh Instrument. The fluorescence quantum yield of **2** was measured to be 15.4% % in CH_2Cl_2, 44.6% in toluene, and that of **1** was measured to be 2.9% % in CH_2Cl_2, 24.3% in toluene.

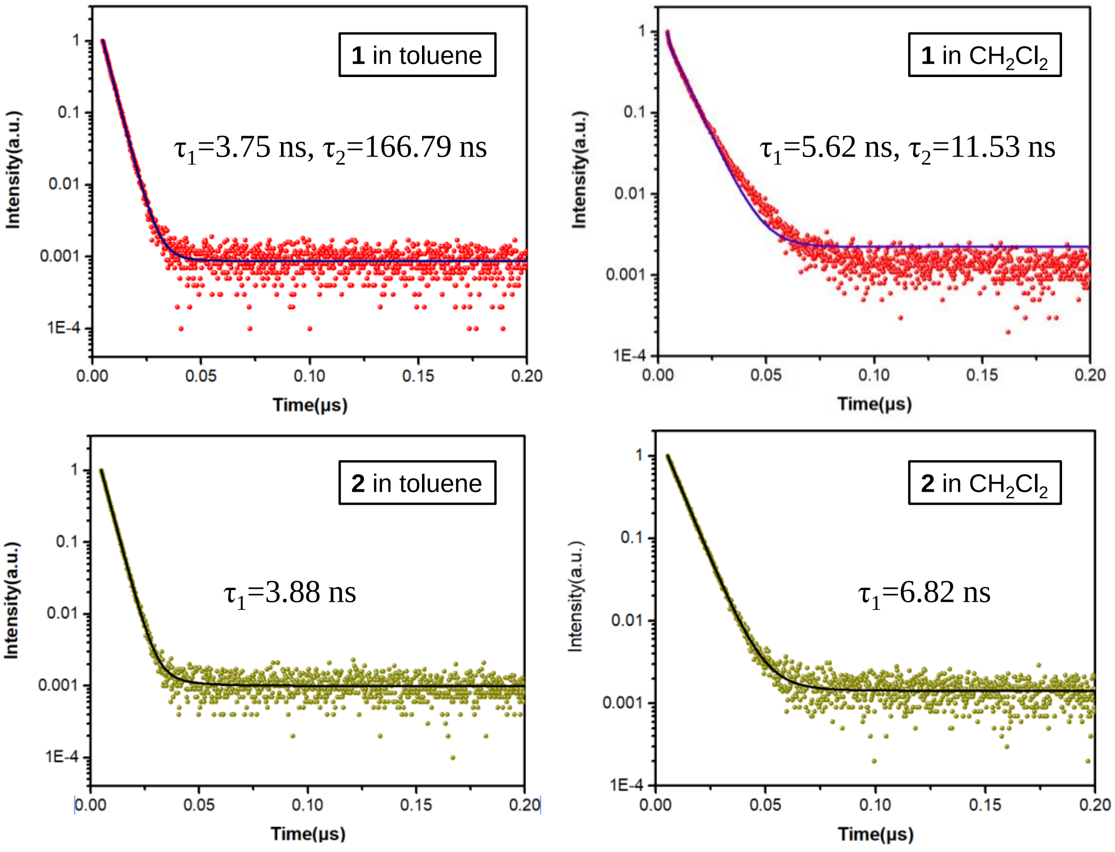

Figure S45. Fluorescence lifetime of **1** and **2** in toluene and CH_2Cl_2, respectively.

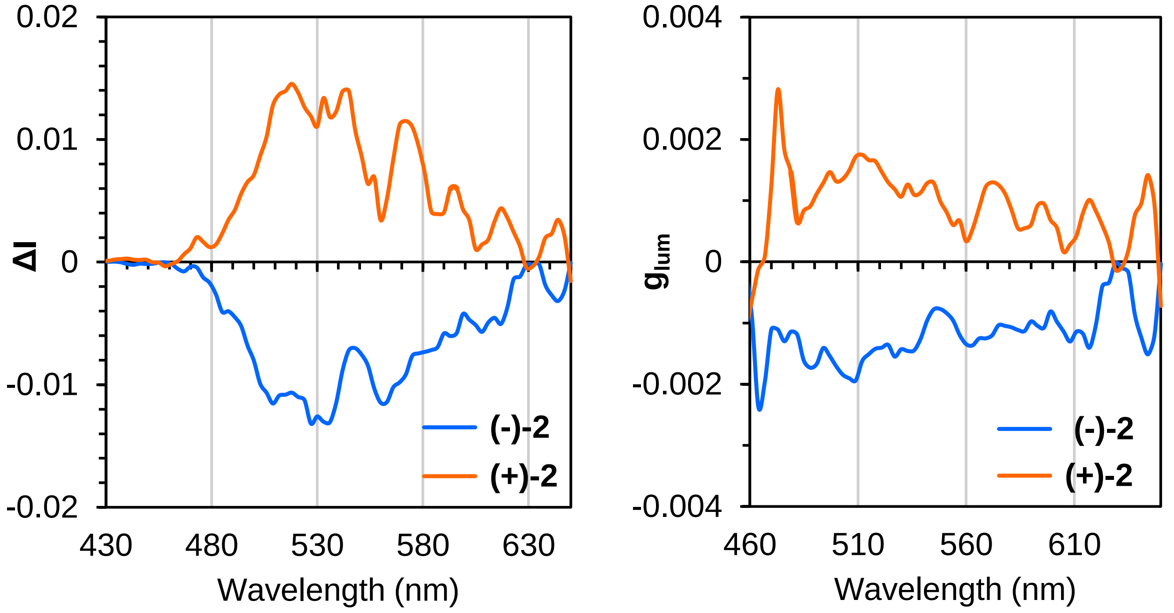

Figure S46. CPL spectra of (+)-**2** and **(-)-2** in CH_2Cl_2 excited at 370 nm

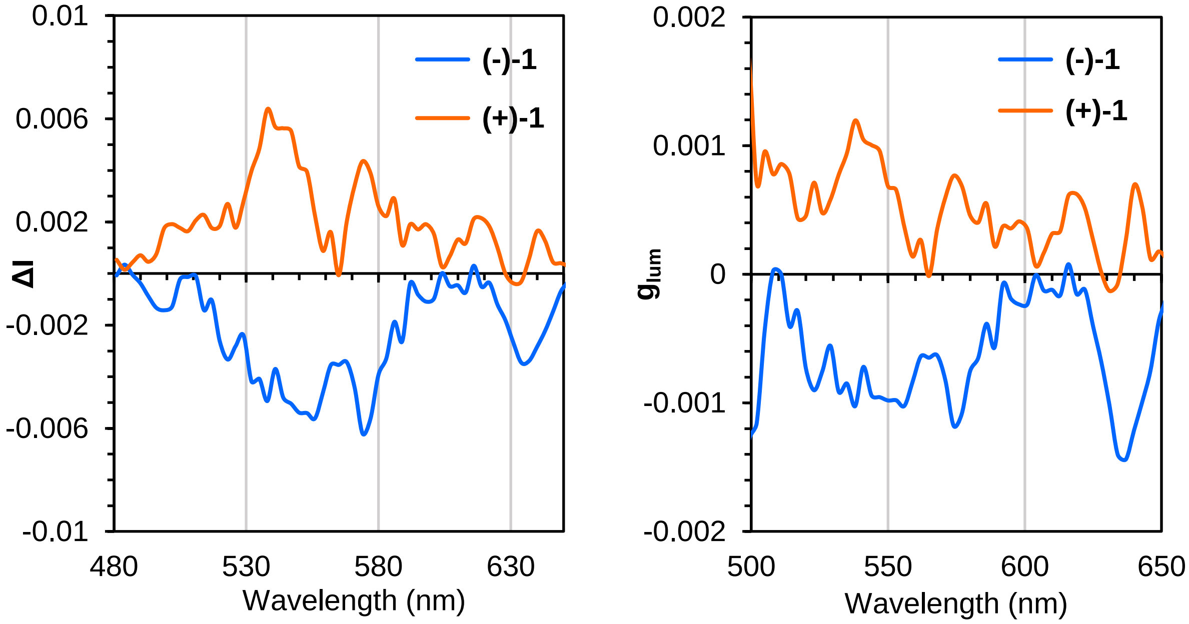

Figure S47. CPL spectra of (+)-**1** and **(-)-1** in CH_2Cl_2 excited at 460 nm.

5. X-ray crystallography data

X-ray crystallography data were collected on a Bruker AXS Kappa ApexII Duo Diffractometer.

Table S1. Crystallographic Data of **3b**

Empirical formula	$C_{42} H_{22} N_4$	
Formula weight	582.63	
Temperature	174(2) K	
Wavelength	1.54178 Å	
Crystal system	Orthorhombic	
Space group	$P2_12_12$	
Unit cell dimensions	a = 12.0169(9) Å	α = 90°.
	b = 12.4906(10) Å	β = 90°.
	c = 9.1848(9) Å	γ = 90°.
Volume	1378.6(2) $Å^3$	
Z	2	
Density (calculated)	1.404 $\times 10^3$ kg/m^3	
Absorption coefficient	0.650 mm^{-1}	
F(000)	604	
Crystal size	0.500 × 0.400 × 0.300 mm^3	
Theta range for data collection	4.815 to 68.310°.	
Index ranges	-14<=h<=14, -15<=k<=15, -11<=l<=11	
Reflections collected	18261	
Independent reflections	2529 [R(int) = 0.0641]	
Completeness to theta = 67.679°	99.7 %	
Absorption correction	multi-scan	
Max. and min. transmission	0.7531 and 0.4602	
Refinement method	Full-matrix least-squares on F^2	
Data / restraints / parameters	2529 / 0 / 208	
Goodness-of-fit on F^2	1.100	
Final R indices [I>2sigma(I)]	R1 = 0.0477, wR2 = 0.1212	
R indices (all data)	R1 = 0.0480, wR2 = 0.1217	
Absolute structure parameter	0.51(15)	
Extinction coefficient	n/a	
Largest diff. peak and hole	0.266 and -0.313 e.$Å^{-3}$	

Table S2. Crystallographic Data of **1**

Empirical formula	$C_{134}H_{120}N_4O_6$	
Formula weight	1882.33	
Temperature (K)	150(2)	
Wavelength (Å)	1.34139	
Crystal system	tetragonal	
Space group	$P4_12_12$	
Unit cell dimensions	a = 17.8312(11) Å	α = 90 °
	b = 17.8312(11) Å	β = 90 °
	c = 91.259(9) Å	γ = 90 °
V	29016(5) Å^3	
Z	8	
ρ_{calcd} (g·cm^{-3})	0.862	
μ (mm^{-1})	0.257	
F(000)	8000	
Crystal size (mm)	0.02×0.09×0.1	
θ range for data collection (°)	5.112-51.212	
Reflections collected	71700	
Independent reflections	23576	
	[R_{int} = 0.1291]	
Transmission factors (min/max)	0.4576/0.7501	
Data/restraints/parameters	23576/749/1551	
$R1$,[a] $wR2$[b] ($I > 2\sigma(I)$)	0.0808, 0.2093	
$R1$,[a] $wR2$[b] (all data)	0.1063, 0.2318	
Quality-of-fit[c]	0.993	

$R_{int} = \Sigma|F_o^2 - <F_o^2>|/\Sigma|F_o^2|$
[a] $R1 = \Sigma||F_o|-|F_c||/\Sigma|F_o|$. [b] $wR2 = [\Sigma[w(F_o^2-F_c^2)^2]/\Sigma[w(F_o^2)^2]]$.
[c] Quality-of-fit = $[\Sigma[w(F_o^2-F_c^2)^2]/(N_{obs}-N_{params})]^{1/2}$, based on all data.

6. Density functional theory (DFT) calculations

All the DFT calculations were done by using Gaussian 16 program,[4] under B3LYP-D3(BJ)/6-311G(d, p) level of theory. Gauge-Independent Atomic Orbital (GIAO) method was utilized for computing NICS values. Continuous Set of Gauge Transformations (CSGT) method was used for generating data files of ACID program to visualize the anisotropy of induced current density.[5]

1

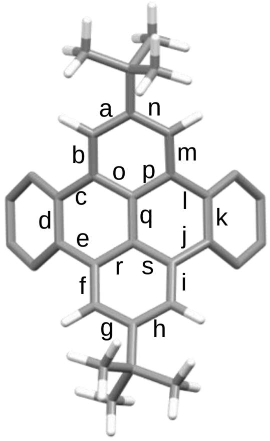

2

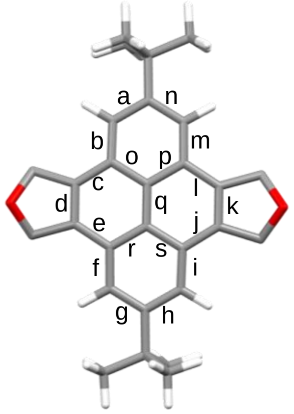

Bond	Bond length (Å)	
	**1**	**2**
a	1.397	1.396
b	1.397	1.400
c	1.477	1.427
d	1.444	1.363
e	1.477	1.427
f	1.392	1.404
g	1.403	1.392
h	1.397	1.396
i	1.397	1.400
j	1.477	1.472
k	1.444	1.363
l	1.477	1.427
m	1.392	1.404
n	1.403	1.392
o	1.406	1.432
p	1.408	1.429
q	1.438	1.428
r	1.408	1.429
s	1.406	1.432

Figure S48. Bond length analysis of **1** and **2** in pyrene moieties based on optimized geometries.

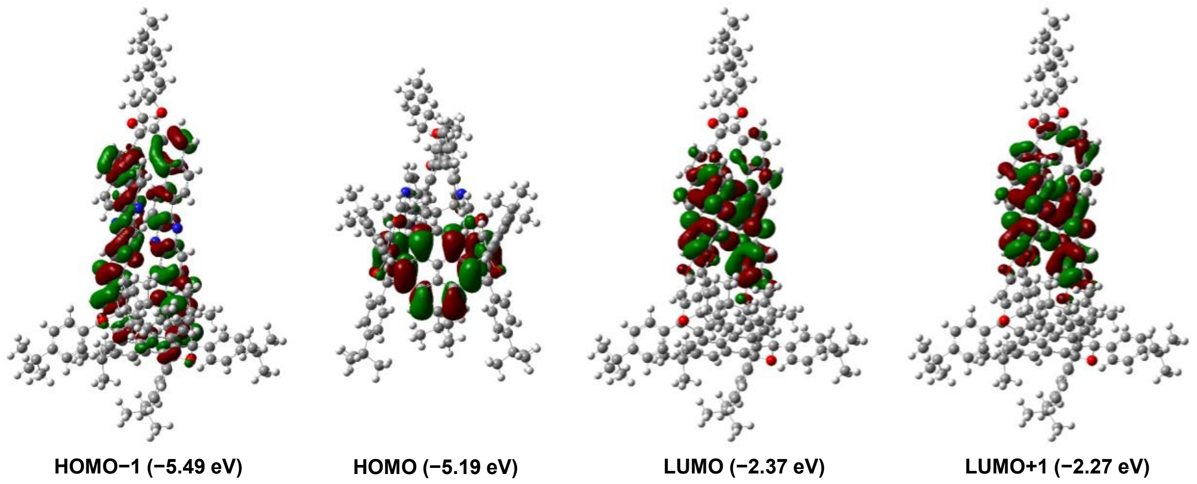

Figure S49. Frontier molecular orbitals of **2** calculated at B3LYP-D3(BJ) level of theory with 6-311g(d,p) basis set.

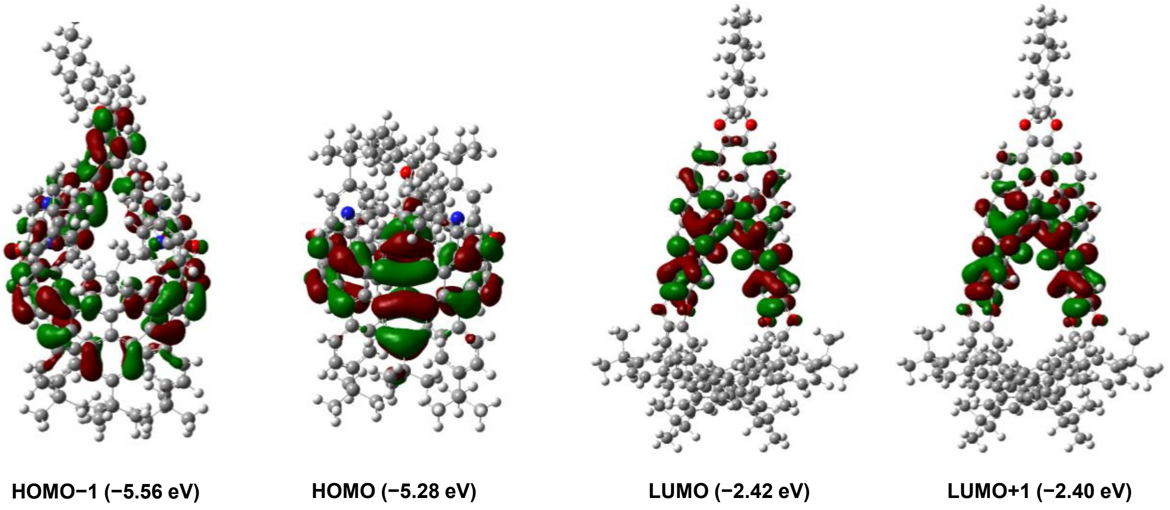

Figure S50. Frontier molecular orbitals of **1** calculated at B3LYP-D3(BJ) level of theory with 6-311g(d,p) basis set.

Estimation of the energy barrier for bond rotation

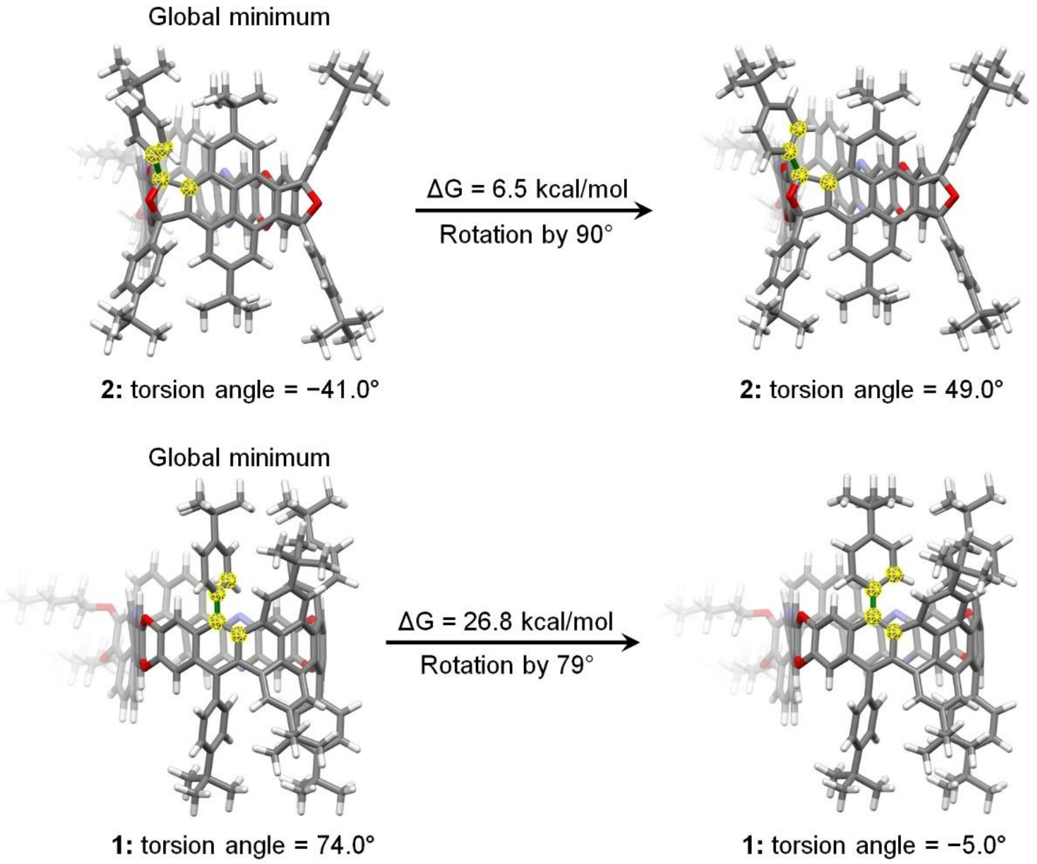

Figure S51. Calculated rotation of the green bond in **1** and **2** with change of Gibbs free energy.

In molecules **1** and **2**, rotating the bond connecting the 4-*tert*-butylphenyl to the macrocycle (shown in green) changes the torsion angles defined by the four highlighted carbon atoms, as shown in Figure S51. For molecule **2**, the energy barrier for this rotation was estimated by optimizing the structure at a maximal torsion angle change of 90 ° and then calculating the

Gibbs free energy change. However, attempts to adjust molecule **1**'s torsion angle by 90 ° resulted in structural optimization failures due to prohibitively short atomic distances. As a result, the structure with a 79 °torsion angle was optimized and its corresponding Gibbs energy was calculated.

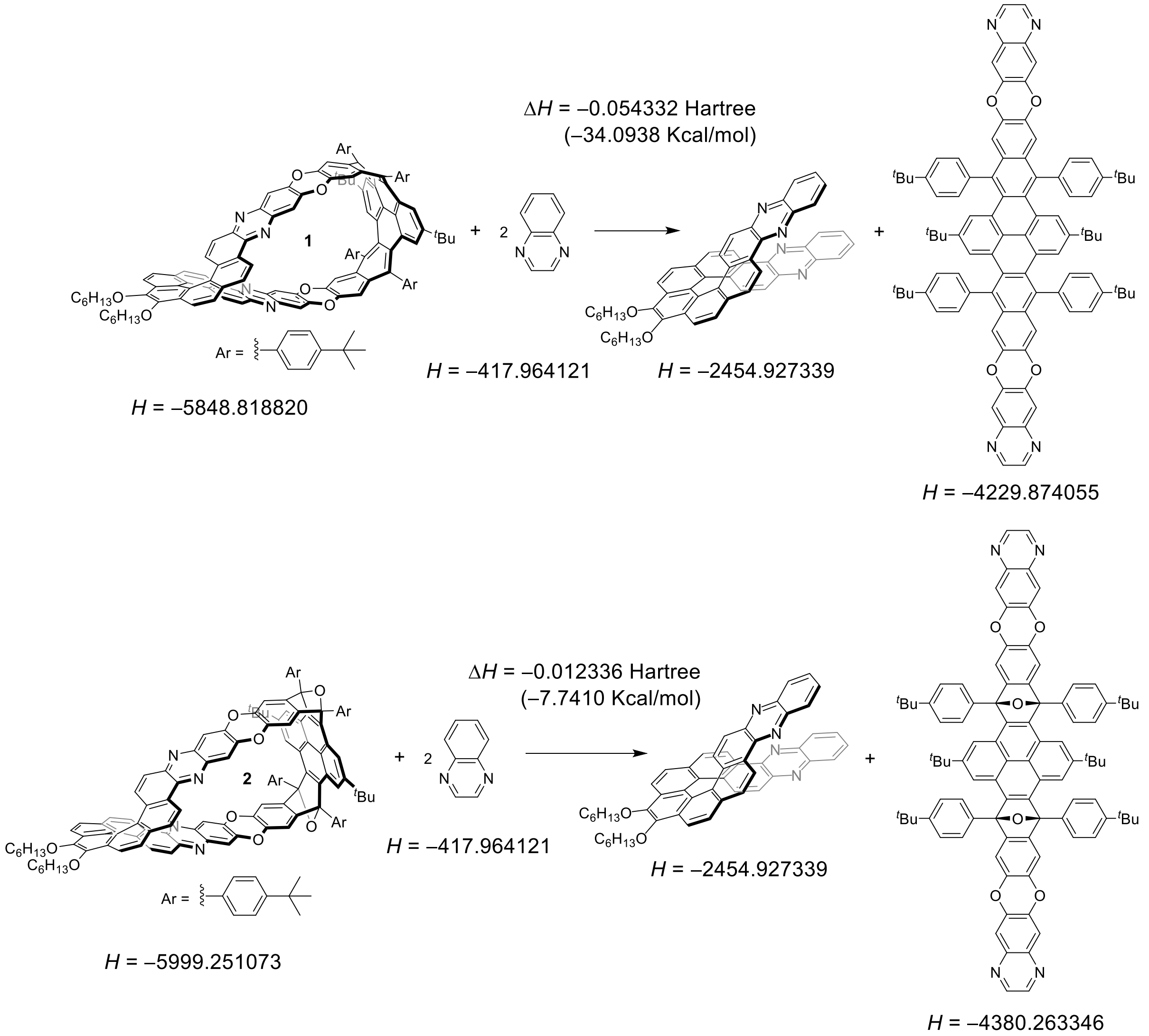

Figure S52. Hypothetical homodesmotic reactions for calculation of strain in compound **1** and **2**.

Calculation of charge separation

The structure of the excited state (S_1) of nanobelt **1** was optimized by time-dependent density functional theory (TD-DFT) at B3LYP-D3(BJ)/6-31g(d, p) level of theory. The charge distribution was calculated this optimized structure at B3LYP-D3(BJ)/6-311g(d, p) level of theory based on in vacuum, DCM and toluene by integral equation formalism polarizable continuum model (IEF-PCM).

Table S3 Accumulated Mulliken charges in the three parts of nanobelt **1** at ground state (S_0) and excited state (S_1).

Fragment		(1)	(2)	(3)
Vacuum	S0	-0.638	0.113	0.525
	S1	-0.011	-0.620	0.503
CH_2Cl_2	S0	-0.638	0.125	0.513
	S1	0.117	-0.548	0.485
Toluene	S0	-0.637	0.142	0.497
	S1	0.063	-0.453	0.463

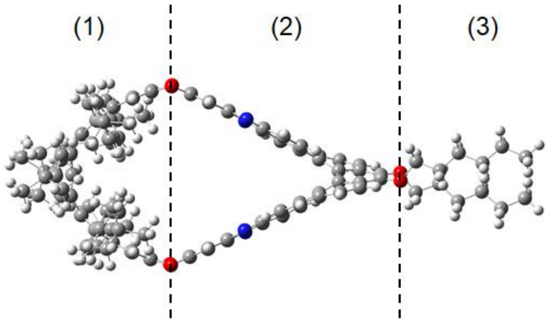

Optimized Cartesian coordinates

Compound **1**:

C	-3.30420	7.00250	-4.43130
C	-4.42280	7.78430	-5.13710
C	-1.96250	7.68630	-4.76100
H	-4.39280	8.83040	-4.82270
H	-4.30670	7.75800	-6.22380
H	-5.41210	7.39390	-4.88650
C	-3.54700	7.06670	-2.90780
C	-3.26810	5.52920	-4.85620
H	-1.12610	7.20610	-4.24980
H	-1.98480	8.73260	-4.44360
H	-1.76300	7.65810	-5.83350
H	-4.49470	6.58920	-2.64660
H	-3.58150	8.10700	-2.57080
H	-2.75290	6.55850	-2.35630
C	-2.23690	4.69420	-4.40190
C	-4.25590	4.94620	-5.65250
C	-2.20640	3.34330	-4.71200
H	-1.44860	5.10010	-3.77920
C	-4.23510	3.58430	-5.95840
H	-5.07040	5.54590	-6.03520
C	-3.21530	2.76270	-5.48840
H	-1.40400	2.71820	-4.33820
H	-5.03090	3.15500	-6.55690
C	-3.24040	1.28210	-5.68330
C	-2.37920	0.62790	-6.55900
C	-3.97570	0.53180	-4.72080
C	-2.33410	-0.81530	-6.58880
C	-1.27300	1.36680	-7.20090
C	-4.53320	1.17980	-3.58300

C	-3.93270	-0.89780	-4.74950
C	-3.16200	-1.55850	-5.74850
C	-1.16780	-1.45310	-7.23360
C	-1.30750	2.65910	-7.71840
C	-0.02560	0.71870	-7.11760
C	-4.91370	0.44730	-2.49870
H	-4.54880	2.25700	-3.51190
C	-4.43770	-1.62660	-3.63330
C	-3.10150	-3.04870	-5.68440
C	-1.09740	-2.73930	-7.77430
C	0.02560	-0.71870	-7.11760
C	-0.13060	3.33050	-8.08080
H	-2.26080	3.14980	-7.84330
C	1.16780	1.45310	-7.23360
C	-4.86200	-0.96270	-2.51920
O	-5.26180	1.07790	-1.31500
H	-4.37640	-2.70470	-3.60820
C	-4.12340	-3.82650	-6.22170
C	-2.04320	-3.70240	-5.04390
C	0.13060	-3.33050	-8.08080
H	-2.01180	-3.28110	-7.94780
C	1.27300	-1.36680	-7.20090
C	-0.22710	4.72130	-8.71740
C	1.09740	2.73930	-7.77430
C	2.33410	0.81530	-6.58880
O	-5.16270	-1.65140	-1.35260
C	-4.76290	0.42920	-0.20670
C	-4.09090	-5.21820	-6.12850
H	-4.95490	-3.33990	-6.71880
C	-2.01350	-5.08630	-4.95650
H	-1.23350	-3.11620	-4.62620
C	0.22710	-4.72130	-8.71740
C	1.30750	-2.65910	-7.71840
C	2.37920	-0.62790	-6.55900
C	1.15530	5.30390	-9.04970
C	-0.93270	5.67390	-7.73180
C	-1.04510	4.63000	-10.02180
H	2.01180	3.28110	-7.94780
C	3.16200	1.55850	-5.74850
C	-4.73000	-0.99920	-0.21700
C	-4.28460	1.13850	0.85330
C	-3.03960	-5.88020	-5.49150
H	-4.90730	-5.78090	-6.55960
H	-1.17340	-5.55570	-4.45870
C	-1.15530	-5.30390	-9.04970
C	0.93270	-5.67390	-7.73180
C	1.04510	-4.63000	-10.02180
H	2.26080	-3.14980	-7.84330
C	3.24040	-1.28210	-5.68330
H	1.70050	4.66950	-9.75350

H	1.03460	6.28710	-9.51210
H	1.76750	5.42840	-8.15450
H	-1.94690	5.34470	-7.50300
H	-0.98850	6.68260	-8.15280
H	-0.38220	5.72360	-6.79180
H	-0.56360	3.95550	-10.73460
H	-1.12960	5.61710	-10.48600
H	-2.05500	4.25800	-9.83730
C	3.93270	0.89780	-4.74950
C	3.10150	3.04870	-5.68440
C	-4.26060	-1.69850	0.85600
C	-3.74860	0.44550	1.97250
H	-4.27800	2.21980	0.84180
C	-2.98080	-7.40670	-5.35070
H	-1.70050	-4.66950	-9.75350
H	-1.03460	-6.28710	-9.51210
H	-1.76750	-5.42840	-8.15450
H	1.94690	-5.34470	-7.50300
H	0.98850	-6.68260	-8.15280
H	0.38220	-5.72360	-6.79180
H	0.56360	-3.95550	-10.73460
H	1.12960	-5.61710	-10.48600
H	2.05500	-4.25800	-9.83730
C	3.97570	-0.53180	-4.72080
C	3.21530	-2.76270	-5.48840
C	4.43770	1.62660	-3.63330
C	4.12340	3.82650	-6.22170
C	2.04320	3.70240	-5.04390
C	-3.76000	-0.99420	1.98770
H	-4.23120	-2.77960	0.84810
N	-3.20440	1.14560	2.97900
C	-4.21300	-8.09490	-5.95760
C	-1.72550	-7.94290	-6.06830
C	-2.90970	-7.77500	-3.85350
C	4.53320	-1.17980	-3.58300
C	4.23510	-3.58430	-5.95840
C	2.20640	-3.34330	-4.71200
C	4.86200	0.96270	-2.51920
H	4.37640	2.70470	-3.60820
C	4.09090	5.21820	-6.12850
H	4.95490	3.33990	-6.71880
C	2.01350	5.08630	-4.95650
H	1.23350	3.11620	-4.62620
N	-3.27210	-1.67780	3.03560
C	-2.68290	0.46530	3.99760
H	-5.13720	-7.77280	-5.47130
H	-4.29970	-7.89660	-7.02890
H	-4.12810	-9.17650	-5.82720
H	-1.75590	-7.70790	-7.13440
H	-1.66200	-9.02940	-5.95790

H	-0.81210	-7.51180	-5.65850
H	-3.78540	-7.39790	-3.31940
H	-2.87500	-8.86160	-3.73250
H	-2.02070	-7.35720	-3.37680
C	4.91370	-0.44730	-2.49870
H	4.54880	-2.25700	-3.51190
C	4.25590	-4.94620	-5.65250
H	5.03090	-3.15500	-6.55690
C	2.23690	-4.69420	-4.40190
H	1.40400	-2.71820	-4.33820
O	5.16270	1.65140	-1.35260
C	3.03960	5.88020	-5.49150
H	4.90730	5.78090	-6.55960
H	1.17340	5.55570	-4.45870
C	-2.74460	-0.96930	4.03700
C	-2.07690	1.19310	5.09840
O	5.26180	-1.07790	-1.31500
C	3.26810	-5.52920	-4.85620
H	5.07040	-5.54590	-6.03520
H	1.44860	-5.10010	-3.77920
C	4.73000	0.99920	-0.21700
C	2.98080	7.40670	-5.35070
C	-2.27380	-1.63640	5.21150
C	-2.08090	2.60750	5.07520
C	-1.55810	0.49160	6.20980
C	4.76290	-0.42920	-0.20670
C	3.30420	-7.00250	-4.43130
C	4.26060	1.69850	0.85600
C	4.21300	8.09490	-5.95760
C	1.72550	7.94290	-6.06830
C	2.90970	7.77500	-3.85350
C	-1.73290	-0.93960	6.24260
H	-2.40070	-2.71040	5.25930
C	-1.69320	3.30640	6.18010
H	-2.46230	3.10100	4.19250
C	-1.00330	1.23380	7.31510
C	4.28460	-1.13850	0.85330
C	4.42280	-7.78430	-5.13710
C	1.96250	-7.68630	-4.76100
C	3.54700	-7.06670	-2.90780
C	3.76000	0.99420	1.98770
H	4.23120	2.77960	0.84810
H	5.13720	7.77280	-5.47130
H	4.29970	7.89660	-7.02890
H	4.12810	9.17650	-5.82720
H	1.75590	7.70790	-7.13440
H	1.66200	9.02940	-5.95790
H	0.81210	7.51180	-5.65850
H	3.78540	7.39790	-3.31940
H	2.87500	8.86160	-3.73250

H	2.02070	7.35720	-3.37680
H	-1.45740	-1.47450	7.13620
C	-1.20590	2.64110	7.33330
H	-1.77620	4.38720	6.20540
C	-0.34380	0.63310	8.46140
C	3.74860	-0.44550	1.97250
H	4.27800	-2.21980	0.84180
H	4.39280	-8.83040	-4.82270
H	4.30670	-7.75800	-6.22380
H	5.41210	-7.39390	-4.88650
H	1.12610	-7.20610	-4.24980
H	1.98480	-8.73260	-4.44360
H	1.76300	-7.65810	-5.83350
H	4.49470	-6.58920	-2.64660
H	3.58150	-8.10700	-2.57080
H	2.75290	-6.55850	-2.35630
N	3.27210	1.67780	3.03560
C	-0.98480	3.37960	8.53200
C	-0.38840	1.34720	9.68990
C	0.34380	-0.63310	8.46140
N	3.20440	-1.14560	2.97900
C	2.74460	0.96930	4.03700
C	-0.68480	2.74080	9.69390
H	-1.14890	4.45110	8.51550
C	-0.12150	0.67830	10.91940
C	0.38840	-1.34720	9.68990
C	1.00330	-1.23380	7.31510
C	2.68290	-0.46530	3.99760
C	2.27380	1.63640	5.21150
H	-0.64140	3.26770	10.63670
C	0.12150	-0.67830	10.91940
O	-0.25210	1.40380	12.07380
C	0.68480	-2.74080	9.69390
C	1.20590	-2.64110	7.33330
C	1.55810	-0.49160	6.20980
C	2.07690	-1.19310	5.09840
C	1.73290	0.93960	6.24260
H	2.40070	2.71040	5.25930
O	0.25210	-1.40380	12.07380
C	0.98480	-3.37960	8.53200
H	0.64140	-3.26770	10.63670
C	1.69320	-3.30640	6.18010
C	2.08090	-2.60750	5.07520
H	1.45740	1.47450	7.13620
H	1.14890	-4.45110	8.51550
H	1.77620	-4.38720	6.20540
H	2.46230	-3.10100	4.19250
C	0.88030	1.42550	12.97150
C	0.36010	1.80400	14.34710
H	1.60850	2.15290	12.59590

H	1.35170	0.44180	12.99230
C	1.39620	1.62150	15.45750
H	-0.00560	2.83570	14.33120
H	-0.50710	1.17280	14.56100
H	1.79840	0.60200	15.41190
H	2.24820	2.29250	15.29610
C	-0.88030	-1.42550	12.97150
C	-0.36010	-1.80400	14.34710
H	-1.60850	-2.15290	12.59590
H	-1.35170	-0.44180	12.99230
C	-1.39620	-1.62150	15.45750
H	0.00560	-2.83570	14.33120
H	0.50710	-1.17280	14.56100
H	-1.79840	-0.60200	15.41190
H	-2.24820	-2.29250	15.29610
C	-0.80720	-1.86230	16.84950
C	-1.78230	-1.56160	17.98920
H	0.08670	-1.23780	16.97140
H	-0.46400	-2.90120	16.92630
C	-1.17530	-1.80880	19.37120
H	-2.10980	-0.51780	17.91340
H	-2.68390	-2.17310	17.86750
H	-1.88410	-1.57280	20.16910
H	-0.87720	-2.85500	19.48850
H	-0.28380	-1.19310	19.52420
C	0.80720	1.86230	16.84950
C	1.78230	1.56160	17.98920
H	-0.08670	1.23780	16.97140
H	0.46400	2.90120	16.92630
C	1.17530	1.80880	19.37120
H	2.10980	0.51780	17.91340
H	2.68390	2.17310	17.86750
H	1.88410	1.57280	20.16910
H	0.87720	2.85500	19.48850
H	0.28380	1.19310	19.52420

Compound **2**:

C	2.51550	3.12560	-5.83420
C	3.94270	3.11250	-6.28440
C	2.13570	3.27660	-4.34620
C	4.22740	2.76660	-7.60940
C	5.00130	3.43920	-5.44490
C	1.57740	1.97170	-6.27450
O	1.81350	4.25860	-6.39730
C	0.84780	3.84020	-4.35810
C	2.66800	2.74910	-3.18890
C	5.53590	2.70930	-8.05920
H	3.41020	2.52380	-8.27810
C	6.31870	3.37400	-5.90110

H	4.80760	3.75310	-4.42800
C	1.80950	0.56400	-6.29400
C	0.32350	2.50670	-6.28130
C	0.50020	3.98850	-5.85410
C	0.08980	3.90580	-3.20770
C	1.91090	2.83740	-2.01640
H	3.62170	2.23970	-3.15250
C	6.61690	2.99630	-7.21030
H	5.71990	2.42620	-9.08860
H	7.11330	3.62450	-5.21190
C	3.08510	-0.00910	-6.16940
C	0.65710	-0.27900	-6.35550
C	-0.85080	1.69660	-6.29940
C	-0.49350	4.99850	-6.33600
C	0.64950	3.41940	-2.02140
H	-0.92460	4.28100	-3.18540
O	2.45170	2.29690	-0.87010
C	8.05080	2.88110	-7.73960
C	3.27100	-1.38770	-6.11140
H	3.93540	0.64910	-6.11410
C	-0.65710	0.27900	-6.35550
C	0.85080	-1.69660	-6.29940
C	-2.14610	2.21190	-6.17500
C	-0.94520	4.92260	-7.65760
C	-0.98720	6.01520	-5.52680
O	-0.10740	3.51510	-0.87230
C	1.69510	2.34370	0.27180
C	9.09650	3.24370	-6.67410
C	8.23190	3.83360	-8.94010
C	8.30580	1.42720	-8.19180
C	4.65990	-2.02460	-5.98290
C	2.14610	-2.21190	-6.17500
C	-1.80950	-0.56400	-6.29400
C	-0.32350	-2.50670	-6.28130
C	-3.27100	1.38770	-6.11140
H	-2.26940	3.28230	-6.12270
C	-1.89670	5.81030	-8.13160
H	-0.55780	4.14390	-8.30360
C	-1.95000	6.90430	-6.00710
H	-0.62180	6.12570	-4.51460
C	0.41410	2.98150	0.27870
C	2.19760	1.78080	1.40740
H	8.98140	4.27430	-6.32930
H	9.03610	2.58120	-5.80700
H	10.09910	3.14530	-7.09750
H	8.04990	4.86930	-8.64270
H	9.25160	3.76210	-9.32930
H	7.54510	3.59330	-9.75420
H	8.18210	0.73490	-7.35550
H	9.32450	1.32320	-8.57630

H	7.61610	1.12410	-8.98220
C	5.77810	-0.97140	-5.93840
C	4.71770	-2.86040	-4.68690
C	4.90390	-2.94740	-7.19630
H	2.26940	-3.28230	-6.12270
C	-3.08510	0.00910	-6.16940
C	-1.57740	-1.97170	-6.27450
C	-0.50020	-3.98850	-5.85410
C	-4.65990	2.02460	-5.98290
C	-2.43660	6.81400	-7.31150
H	-2.23390	5.71260	-9.15640
H	-2.31670	7.67270	-5.34060
C	-0.30960	3.07700	1.43200
C	1.47070	1.86100	2.62430
H	3.16130	1.29020	1.39610
H	5.67640	-0.30650	-5.07690
H	6.74730	-1.47060	-5.86010
H	5.79170	-0.35460	-6.83990
H	4.52710	-2.23310	-3.81250
H	5.70590	-3.31610	-4.57390
H	3.97740	-3.66320	-4.69430
H	4.86310	-2.37760	-8.12830
H	5.89040	-3.41490	-7.12390
H	4.15860	-3.74280	-7.25560
H	-3.93540	-0.64910	-6.11410
C	-2.51550	-3.12560	-5.83420
C	0.49350	-4.99850	-6.33600
C	-0.84780	-3.84020	-4.35810
O	-1.81350	-4.25860	-6.39730
C	-5.77810	0.97140	-5.93840
C	-4.71770	2.86040	-4.68690
C	-4.90390	2.94740	-7.19630
C	-3.52030	7.74660	-7.86440
C	0.20380	2.54180	2.64510
H	-1.27010	3.57410	1.43730
N	1.98950	1.32670	3.73820
C	-3.94270	-3.11250	-6.28440
C	-2.13570	-3.27660	-4.34620
C	0.94520	-4.92260	-7.65760
C	0.98720	-6.01520	-5.52680
C	-0.08980	-3.90580	-3.20770
H	-5.67640	0.30650	-5.07690
H	-6.74730	1.47060	-5.86010
H	-5.79170	0.35460	-6.83990
H	-4.52710	2.23310	-3.81250
H	-5.70590	3.31610	-4.57390
H	-3.97740	3.66320	-4.69430
H	-4.86310	2.37760	-8.12830
H	-5.89040	3.41490	-7.12390
H	-4.15860	3.74280	-7.25560

C	-3.97930	8.78510	-6.82960
C	-2.97050	8.49870	-9.09460
C	-4.74490	6.90350	-8.28010
N	-0.47940	2.70280	3.78840
C	1.30170	1.47010	4.87000
C	-4.22740	-2.76660	-7.60940
C	-5.00130	-3.43920	-5.44490
C	-2.66800	-2.74910	-3.18890
C	1.89670	-5.81030	-8.13160
H	0.55780	-4.14390	-8.30360
C	1.95000	-6.90430	-6.00710
H	0.62180	-6.12570	-4.51460
C	-0.64950	-3.41940	-2.02140
H	0.92460	-4.28100	-3.18540
H	-3.15620	9.43010	-6.51190
H	-4.40720	8.31030	-5.94300
H	-4.75030	9.42280	-7.26890
H	-2.09720	9.09700	-8.82340
H	-3.73380	9.16910	-9.50000
H	-2.67240	7.81150	-9.88890
H	-5.14980	6.36010	-7.42280
H	-5.53170	7.54980	-8.67970
H	-4.48890	6.17120	-9.04840
C	0.05940	2.19120	4.89910
C	1.82670	0.89380	6.09140
C	-5.53590	-2.70930	-8.05920
H	-3.41020	-2.52380	-8.27810
C	-6.31870	-3.37400	-5.90110
H	-4.80760	-3.75310	-4.42800
C	-1.91090	-2.83740	-2.01640
H	-3.62170	-2.23970	-3.15250
C	2.43660	-6.81400	-7.31150
H	2.23390	-5.71260	-9.15640
H	2.31670	-7.67270	-5.34060
O	0.10740	-3.51510	-0.87230
C	-0.57520	2.40850	6.16290
C	1.08550	1.00000	7.28480
C	3.08800	0.25060	6.08530
C	-6.61690	-2.99630	-7.21030
H	-5.71990	-2.42620	-9.08860
H	-7.11330	-3.62450	-5.21190
O	-2.45170	-2.29690	-0.87010
C	3.52030	-7.74660	-7.86440
C	-0.41410	-2.98150	0.27870
C	-0.07410	1.85410	7.29520
H	-1.45610	3.03700	6.17790
C	1.53970	0.29450	8.45390
C	3.62670	-0.19990	7.25560
H	3.62100	0.18510	5.14720
C	-8.05080	-2.88110	-7.73960

C	-1.69510	-2.34370	0.27180
C	3.97930	-8.78510	-6.82960
C	2.97050	-8.49870	-9.09460
C	4.74490	-6.90350	-8.28010
C	0.30960	-3.07700	1.43200
H	-0.54820	2.06480	8.24180
C	2.88160	-0.16950	8.46340
C	0.72030	0.03430	9.62240
H	4.63150	-0.60700	7.27950
C	-9.09650	-3.24370	-6.67410
C	-8.23190	-3.83360	-8.94010
C	-8.30580	-1.42720	-8.19180
C	-2.19760	-1.78080	1.40740
H	3.15620	-9.43010	-6.51190
H	4.40720	-8.31030	-5.94300
H	4.75030	-9.42280	-7.26890
H	2.09720	-9.09700	-8.82340
H	3.73380	-9.16910	-9.50000
H	2.67240	-7.81150	-9.88890
H	5.14980	-6.36010	-7.42280
H	5.53170	-7.54980	-8.67970
H	4.48890	-6.17120	-9.04840
C	-0.20380	-2.54180	2.64510
H	1.27010	-3.57410	1.43730
C	3.47480	-0.58700	9.68990
C	1.39080	-0.20220	10.85250
C	-0.72030	-0.03430	9.62240
H	-8.98140	-4.27430	-6.32930
H	-9.03610	-2.58120	-5.80700
H	-10.09910	-3.14530	-7.09750
H	-8.04990	-4.86930	-8.64270
H	-9.25160	-3.76210	-9.32930
H	-7.54510	-3.59330	-9.75420
H	-8.18210	-0.73490	-7.35550
H	-9.32450	-1.32320	-8.57630
H	-7.61610	-1.12410	-8.98220
C	-1.47070	-1.86100	2.62430
H	-3.16130	-1.29020	1.39610
N	0.47940	-2.70280	3.78840
C	2.78170	-0.51490	10.85840
H	4.51070	-0.90720	9.68020
C	0.67040	-0.15850	12.08180
C	-1.39080	0.20220	10.85250
C	-1.53970	-0.29450	8.45390
N	-1.98950	-1.32670	3.73820
C	-0.05940	-2.19120	4.89910
H	3.24990	-0.73010	11.80860
C	-0.67040	0.15850	12.08180
O	1.38620	-0.32150	13.23900
C	-2.78170	0.51490	10.85840

C	-2.88160	0.16950	8.46340
C	-1.08550	-1.00000	7.28480
C	-1.30170	-1.47010	4.87000
C	0.57520	-2.40850	6.16290
O	-1.38620	0.32150	13.23900
C	-3.47480	0.58700	9.68990
H	-3.24990	0.73010	11.80860
C	-3.62670	0.19990	7.25560
C	-1.82670	-0.89380	6.09140
C	0.07410	-1.85410	7.29520
H	1.45610	-3.03700	6.17790
H	-4.51070	0.90720	9.68020
C	-3.08800	-0.25060	6.08530
H	-4.63150	0.60700	7.27950
H	0.54820	-2.06480	8.24180
H	-3.62100	-0.18510	5.14720
C	0.96250	-1.37360	14.13400
C	1.51400	-1.04580	15.51060
H	1.34660	-2.32800	13.75700
H	-0.12730	-1.42180	14.15450
C	0.93710	-1.92680	16.62000
H	2.60640	-1.11610	15.49580
H	1.27520	-0.00020	15.72490
C	1.39040	-1.48220	18.01260
H	-0.15850	-1.89350	16.57410
H	1.21690	-2.97440	16.45750
H	2.48040	-1.57750	18.08980
H	1.17000	-0.41430	18.13530
C	-0.96250	1.37360	14.13400
C	-1.51400	1.04580	15.51060
H	-1.34660	2.32800	13.75700
H	0.12730	1.42180	14.15450
C	-0.93710	1.92680	16.62000
H	-2.60640	1.11610	15.49580
H	-1.27520	0.00020	15.72490
C	-1.39040	1.48220	18.01260
H	0.15850	1.89350	16.57410
H	-1.21690	2.97440	16.45750
H	-2.48040	1.57750	18.08980
H	-1.17000	0.41430	18.13530
C	0.72840	-2.26000	19.15130
C	1.19380	-1.80080	20.53400
H	0.93450	-3.32980	19.02910
H	-0.35990	-2.14900	19.07470
H	0.69690	-2.35960	21.33110
H	2.27280	-1.93950	20.65200
H	0.97960	-0.73890	20.68760
C	-0.72840	2.26000	19.15130
C	-1.19380	1.80080	20.53400
H	-0.93450	3.32980	19.02910

H	0.35990	2.14900	19.07470
H	-0.69690	2.35960	21.33110
H	-2.27280	1.93950	20.65200
H	-0.97960	0.73890	20.68760

7. References

(1) He, Z., Xu, X., Zheng, X., Ming, T., Miao, Q. Conjugated macrocycles of phenanthrene: a new segment of [6,6]-carbon nanotube and solution-processed organic semiconductors. *Chem. Sci.* **4**, 4525-4531 (2013).

(2) Demidoff, F. C., de Souza, F. P., Netto, C. D. Synthesis of stilbene-quinone hybrids through heck reactions in PEG-400. *Synthesis* **49**, 5217-5223 (2017).

(3) Ye, L., Chen, H., Miao, Q. An oxanorbornene-fused nanocage consisting of hexaazatrinaphthylene and pyrene units. *Org. Chem. Front.* **10**, 1003-1008 (2023).

(4) Gaussian 16, Revision C.01, Frisch, M. J.; Trucks, G. W.; Schlegel, H. B.; Scuseria, G. E.; Robb, M. A.; Cheeseman, J. R.; Scalmani, G.; Barone, V.; Petersson, G. A.; Nakatsuji, H.; Li, X.; Caricato, M.; Marenich, A. V.; Bloino, J.; Janesko, B. G.; Gomperts, R.; Mennucci, B.; Hratchian, H. P.; Ortiz, J. V.; Izmaylov, A. F.; Sonnenberg, J. L.; Williams-Young, D.; Ding, F.; Lipparini, F.; Egidi, F.; Goings, J.; Peng, B.; Petrone, A.; Henderson, T.; Ranasinghe, D.; Zakrzewski, V. G.; Gao, J.; Rega, N.; Zheng, G.; Liang, W.; Hada, M.; Ehara, M.; Toyota, K.; Fukuda, R.; Hasegawa, J.; Ishida, M.; Nakajima, T.; Honda, Y.; Kitao, O.; Nakai, H.; Vreven, T.; Throssell, K.; Montgomery, J. A., Jr.; Peralta, J. E.; Ogliaro, F.; Bearpark, M. J.; Heyd, J. J.; Brothers, E. N.; Kudin, K. N.; Staroverov, V. N.; Keith, T. A.; Kobayashi, R.; Normand, J.; Raghavachari, K.; Rendell, A. P.; Burant, J. C.; Iyengar, S. S.; Tomasi, J.; Cossi, M.; Millam, J. M.; Klene, M.; Adamo, C.; Cammi, R.; Ochterski, J. W.; Martin, R. L.; Morokuma, K.; Farkas, O.; Foresman, J. B.; Fox, D. J. Gaussian, Inc., Wallingford CT, 2016.

(5) Geuenich, D., Hess, K., Köhler, F., Herges, R. Anisotropy of the Induced Current Density (ACID), a General Method to Quantify and Visualize Electronic Delocalization. *Chem. Rev.* **105**, 3758-3772 (2005)
